# Targeting Neuraminidase 4 Attenuates Kidney Fibrosis in Mice

**DOI:** 10.1002/advs.202406936

**Published:** 2024-08-13

**Authors:** Ping‐Ting Xiao, Jin‐Hua Hao, Yu‐Jia Kuang, Cai‐Xia Dai, Xiao‐Ling Rong, Li‐Long Jiang, Zhi‐Shen Xie, Lei Zhang, Qian‐Qian Chen, E‐Hu Liu

**Affiliations:** ^1^ School of Pharmacy Nanjing University of Chinese Medicine Nanjing 210023 China; ^2^ State Key Laboratory of Natural Medicines China Pharmaceutical University Nanjing 210009 China; ^3^ PolyU Academy for Interdisciplinary Research The Hong Kong Polytechnic University Hong Kong 999077 China; ^4^ Academy of Chinese Medical Sciences Henan University of Chinese Medicine Zhengzhou 450000 China; ^5^ Hunan Key Laboratory of Kidney Disease and Blood Purification Department of Nephrology The Second Xiangya Hospital Central South University Changsha 410000 China

**Keywords:** 3,5,6,7,8,3ʹ,4ʹ‐Heptamethoxyflavone, NEU4, renal fibrosis, YAP

## Abstract

Despite significant progress in therapy, there remains a lack of substantial evidence regarding the molecular factors that lead to renal fibrosis. Neuraminidase 4 (NEU4), an enzyme that removes sialic acids from glycoconjugates, has an unclear role in chronic progressive fibrosis. Here, this study finds that NEU4 expression is markedly upregulated in mouse fibrotic kidneys induced by folic acid or unilateral ureter obstruction, and this elevation is observed in patients with renal fibrosis. NEU4 knockdown specifically in the kidney attenuates the epithelial‐to‐mesenchymal transition, reduces the production of pro‐fibrotic cytokines, and decreases cellular senescence in male mice. Conversely, NEU4 overexpression exacerbates the progression of renal fibrosis. Mechanistically, NEU4_254‐388aa_ interacts with Yes‐associated protein (YAP) at WW2 domain (231‐263aa), promoting its nucleus translocation and activation of target genes, thereby contributing to renal fibrosis. 3,5,6,7,8,3ʹ,4ʹ‐Heptamethoxyflavone, a natural compound, is identified as a novel NEU4 inhibitor, effectively protecting mice from renal fibrosis in a NEU4‐dependent manner. Collectively, the findings suggest that NEU4 may represent a promising therapeutic target for kidney fibrosis.

## Graphical abstract

The diagram depicts the protective mechanism of HMF on renal fibrosis. Briefly, NEU4 is highly expressed in fibrotic kidneys. Mechanistically, NEU4_254‐388aa_ interacts with Yap at WW2 domain (231‐263aa), preventing its phosphorylation, promoting its nucleus translocation and activation of target genes (*CTGF* et al), thereby promoting renal fibrosis. HMF is screened as a novel NEU4 inhibitor, effectively attenuating renal fibrosis in a NEU4‐dependent manner.

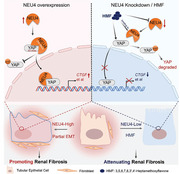

## 1 Introduction

Fibrotic disease is a complex and dynamic disorder characterized by an excessive accumulation of extracellular matrix, subsequently resulting in the formation of a fibrous scar, destruction of organ parenchyma and loss of organ function.^[^ ^1^ ^]^ Currently, there is no treatment available for this condition. However, elucidation of the intricate cellular and molecular pathways involved in organ fibrosis could lead to the development of effective therapeutic strategies and delay of disease progression.^[^ ^2^ ^]^

Renal fibrosis is the end stage of nearly all chronic progressive kidney diseases. The pathogenesis of renal fibrosis involves multiple molecular pathways and various renal and infiltrating cell types.^[^ ^3^ ^]^ Tubular epithelial cells (TECs), the major component of the kidney and the important target in progression of renal fibrosis, possess a restricted capacity for repair.^[^ ^4^, ^5^, ^6^ ^]^ Following injury, residual TECs may undergo alterations such as partial epithelial‐mesenchymal transition (EMT), cellular senescence, cell cycle progression, cell apoptosis, or the secretion of pro‐inflammatory and pro‐fibrotic cytokines, ultimately leading to the onset of kidney fibrosis.^[^ ^7^, ^8^, ^9^, ^10^ ^]^ Notably, accumulating evidence shows that TECs undergo EMT after injury and contribute to production of profibrogenic growth factors, such as transforming growth factor beta 1 (TGF*β*1) and connective tissue growth factor (CTGF).^[^ ^11^, ^12^, ^13^, ^14^ ^]^ These profibrotic factors not only stimulate proliferation and activation of fibroblasts through paracrine effects, leading to increased production and accumulation of extracellular matrix (ECM), but also accelerate the loss of epithelial phenotype in TECs.^[^ ^9^, ^15^, ^16^ ^]^

Neuraminidases, also known as sialidases, are glycohydrolytic enzymes that remove terminal sialic acid residues from sialylated glycoproteins, oligosaccharides, and glycolipids.^[^ ^17^ ^]^ The functions of viral NEUs have been well documented in the influenza virus replication.^[^ ^18^, ^19^ ^]^ The biological effects of mammalian NEUs, however, tend to be underestimated and are less characterized. There are four isoenzymes of mammalian NEUs, namely NEU1, NEU2, NEU3, NEU4, each exhibiting unique subcellular and tissue expression patterns as well as specific substrate preferences. Among them, NEU1 is typically located in lysosomes,^[^ ^4^ ^]^ NEU2 is a cytosolic enzyme, while NEU3 is located in endosomes and plasma membrane.*^[^* ^20^, ^21^ ^]^ The neuraminidase NEU4, is widely distributed within cells, such as cytoplasm, lysosomes, mitochondria, endoplasmic reticulum, and the nucleus.^[^ ^22^, ^23^, ^24^, ^25^ ^]^ Researches have indicated that NEU1 is linked to pulmonary fibrosis, myocardial fibrosis, and renal fibrosis^[^ ^4^ ^]^ and NEU2 is associated with myoblast differentiation,^[^ ^26^ ^]^ while NEU3 is associated with pulmonary fibrosis.^[^ ^27^ ^]^ It is evident that there is a close association between neuraminidases and fibrosis. Of these, the role of NEU4 in diseases, especially chronic kidney disease, however, remain largely unexplored.

Here, to address the existing scientific gray areas and loopholes in respect of the neuraminidases, this work aimed to study the role of NEU4 in renal fibrosis. We detected the NEU4 expression in patients with renal fibrosis, and in male mice subjected to unilateral ureteral obstruction (UUO) or administered folic acid (FA). Kidney‐specific NEU4 knockdown and overexpression mice were generated by adeno‐associated virus (AAV) to characterize the role of NEU4 on the progression of renal fibrosis. Co‐immunoprecipitation and liquid chromatography‐tandem mass spectrometry, bimolecular fluorescence complementation (BiFC), and surface plasmon resonance (SPR) were employed to investigate the underlying mechanisms by which NEU4 promotes renal fibrosis. In addition, natural compounds were screened to bind to mammalian NEU4 and protect kidneys from injury in mice.

## 2 Results

### 2.1 NEU4 was Significantly Increased in Human and Mouse Fibrotic Kidneys

**Figure 1:**
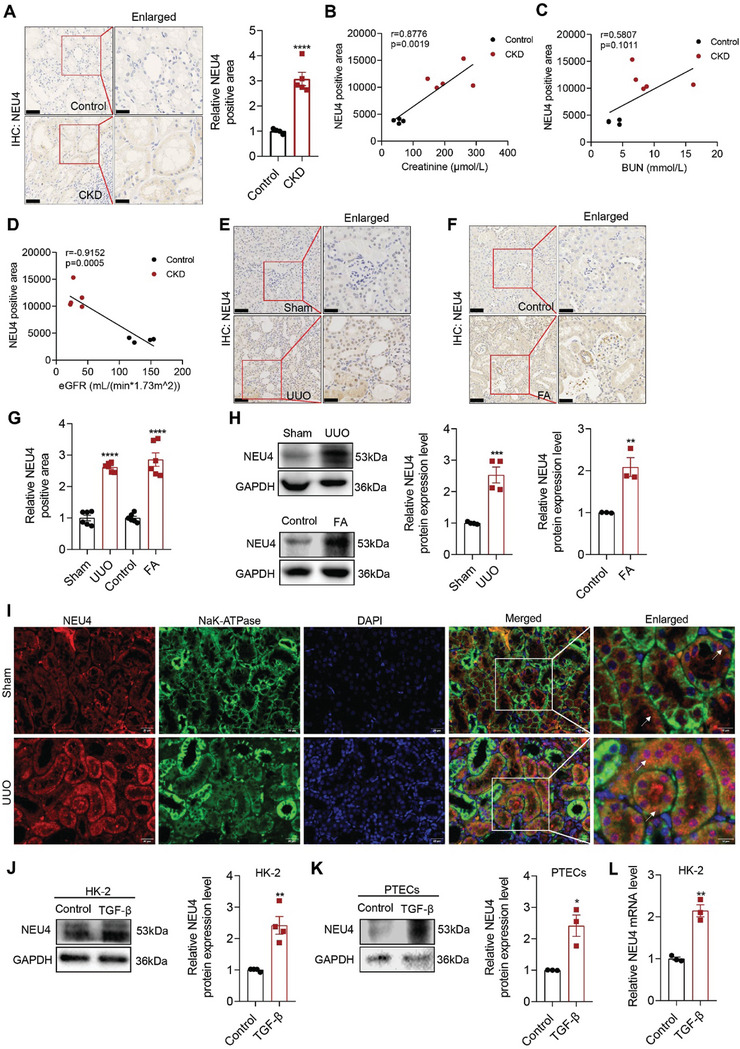
NEU4 was significantly increased in human and mouse fibrotic kidneys. A) Representative immunohistochemical micrographs and quantification of NEU4 expression in kidney from patients with CKD. Scale bar, 50 µm. patients, *n* = 4–5 samples. B–D) Pearson's correlation of NEU4 with serum creatinine level (B), blood urea nitrogen (BUN) (C), and estimated glomerular filtration rate (eGFR) (D) (*n* = 9, Pearson χ2 test). E,F) Representative immunohistochemical micrographs of NEU4 in kidney from mice subjected to UUO (E) and mice subjected to folic acid (F). Scale bar, 50 µm. *n* = 6 mice. G) Quantification of NEU4 expression in Figure 1E,F. H) Western blot (left panel) and quantification (right panel) of the protein expression of NEU4 in left kidneys from mice subjected to UUO or folic acid. GAPDH served as loading control, *n* = 3–4 mice. I) Immunofluorescence images of NEU4 and Na^+^/K^+^‐ATPase in kidney from mice subjected to UUO. Na^+^/K^+^‐ATPase was used as tubular epithelial cell marker. Scale bar, 50 µm. *n* = 3 mice. J) Western blot (left panel) and quantification (right panel) of the protein expression of NEU4 in HK‐2 cells treatment with TGF‐*β* 24 h. GAPDH served as loading control, *n* = 4 samples. K) Western blot (top panel) and quantification (bottom panel) of the protein expression of NEU4 in PTECs treatment with TGF‐*β* 24 h. GAPDH served as loading control, *n* = 3 samples. L) *NEU4* mRNA level in HK‐2 cells treatment with TGF‐*β* 24 h. *n* = 3 samples. Error bars represent mean ± SEM. Comparisons between two groups were analyzed by using a two‐tailed Studentʹs *t* test. *p < 0.05, **p < 0.01, ***p < 0.001, ****p < 0.0001 versus the Sham or Control group.

To clarify the expression of NEU4 in kidney fibrosis, we first analyzed human kidney biopsies samples collected from patients with renal fibrosis (*n* = 5) and without renal fibrosis (noncancerous nephrectomy tissues, *n* = 4). The diagnosis and demographic data of the patients are presented in Table S1 (Supporting Information). Immunohistochemistry (IHC) revealed that NEU4 protein levels were significantly higher in the tubules of kidney sections of patients with renal fibrosis than without renal fibrosis (**Figure** **1**A). Moreover, the highly expressed NEU4 was distributed in both the nucleus and cytoplasm in the renal tubules (Figure 1A). The level of NEU4 showed a strong positive correlation with the level of serum creatinine (Figure 1B), and the blood urea nitrogen (Figure 1C), but showed a negative correlation with the estimated glomerular filtration rate (Figure 1D). The increased level of NEU4 was replicated in two mice models of UUO‐ and FA‐induced renal fibrosis by western blots and immunohistochemistry (Figure 1E‐H). We further performed double‐immunofluorescent staining of NEU4 and markers of tubular epithelial cell (Na^+^K^+^‐ATPase). The results showed increased NEU4 was localized in TECs of fibrotic kidneys (Figure 1I). Next, we determined the levels of NEU4 in TECs. As expected, NEU4 protein (Figure 1J,K) and mRNA (Figure 1L) were found to be significantly increased in human and mouse TECs in response to TGF‐*β*, a primary factor that drives fibrosis.

### 2.2 NEU4 Promoted Epithelial‐Mesenchymal Transition, Programmed Cell Death, and Cellular Senescence in TGF‐*β*‐Induced TECs

**Figure 2:**
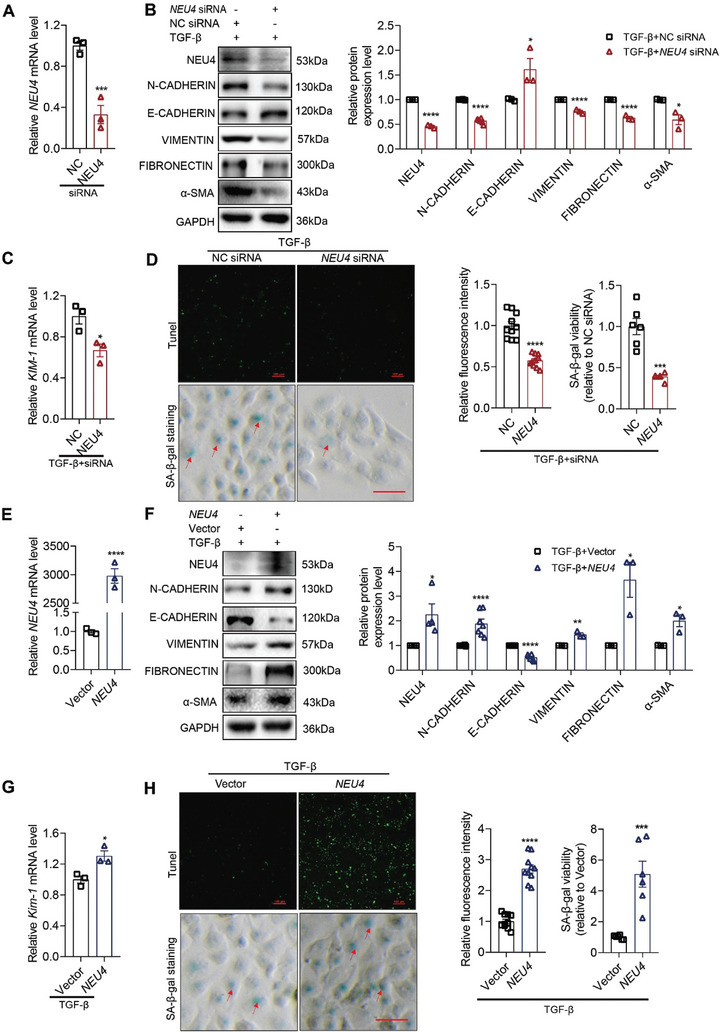
NEU4 promoted epithelial‐mesenchymal transition, programmed cell death, and cellular senescence in TGF‐*β*‐induced HK‐2. A) *NEU4* mRNA level. *n* = 3 samples. B) Western blot (left panel) and quantification (right panel) of the protein expression of NEU4, *α*‐SMA, N‐CADHERIN, E‐CADHERIN, VIMENTIN and FIBRONECTIN in HK‐2 cells. GAPDH served as loading control, *n* = 3–6 samples. C) *Kim‐1* mRNA level. *n* = 3 samples. D) Measurement (left panel) and the quantification (right panel) of apoptosis by TUNEL staining and SA‐*β*‐gal activity by SA‐*β*‐gal staining in HK‐2 cells. *n* = 6–10 samples. Scale bar, 100 µm. E) *NEU4* mRNA level. *n* = 3 samples. F) Western blot (left panel) and quantification (right panel) of the protein expression of NEU4, *α*‐SMA, N‐CADHERIN, E‐CADHERIN, VIMENTIN and FIBRONECTIN in HK‐2 cells. GAPDH served as loading control, *n* = 3–7 samples. G) *Kim‐1* mRNA level. *n* = 3 samples. H) Measurement (left panel) and the quantification (right panel) of apoptosis by TUNEL staining and SA‐*β*‐gal activity by SA‐*β*‐gal staining in HK‐2 cells. *n* = 6–10 samples. Scale bar, 100 µm. (A–D) HK‐2 cells treatment with TGF‐*β* 24 h after transfection with *NEU4* siRNA. (E–H) HK‐2 cells treatment with TGF‐*β* 24 h after transfection with *NEU4*‐overexpression plasmids. Error bars represent mean ± SEM. Comparisons between two groups were analyzed by using a two‐tailed Studentʹs *t* test. *p < 0.05, **p < 0.01, ***p < 0.001, ****p < 0.0001 versus the NC siRNA or Vector group.

Next, we evaluated the detailed function of NEU4 in human renal tubular epithelial cells (HK‐2) in response to TGF‐*β*. Knockdown and overexpression of NEU4 by transfection of siRNA or overexpression plasmid, respectively, were verified in HK‐2 cells by RT‐qPCR (**Figure** **2**A,E) and western blot (Figure 2B,F). E‐Cadherin, a marker of epithelium cells, was upregulated in NEU4‐knockdown HK‐2 cells in the presence of TGF‐*β* (Figure 2B). Vimentin, Fibronectin and *α*‐SMA, the marker of mesenchymal cells, and N‐Cadherin were significantly downregulated when NEU4 was knocked down (Figure 2B). We also observed significant decreases of the mRNA levels of kidney injury molecule (KIM‐1) (Figure 2C), EMT (Figure S1A, Supporting Information), ECM (Figure S1B, Supporting Information) associated genes, *MMP9* (Figure S1C, Supporting Information) and chemokine associated genes (Figure S1D, Supporting Information) by *NEU4* knockdown. As determined by a TUNEL assay, knockdown of NEU4 suppressed cell apoptosis in response to TGF‐*β* (Figure 2D). Moreover, SA‐*β*‐gal‐positive senescent cells in TGF‐*β*‐induced proximal tubules were significantly downregulated when NEU4 was knocked down (Figures 2D; S1E, Supporting Information). RT‐qPCR showed that NEU4 knockdown reduced senescence associated genes (*P16*, *P21*, *P53*, and *IL8*) and DNA damage marker gene (*γH2AX*) mRNA level in TGF‐*β*‐ or H_2_O_2_‐induced HK‐2 cell (Figure S1F‐H, Supporting Information). Additionally, NEU4 knockdown resulted in an increase in proliferation marker *KI67* mRNA level in TGF‐*β*‐induced HK‐2 cell (Figure S1I, Supporting Information). Immunofluorescence assay showed that NEU4 knockdown increased KI67 and decreased *γ*H2AX expression in TGF‐*β*‐induced HK‐2 cell (Figure S1J, Supporting Information). Conversely, NEU4 overexpression promoted TGF‐*β*‐induced EMT and ECM associated genes transcription and protein expression (Figures 2F; S1K,L, Supporting Information), *KIM‐1* mRNA expression (Figure 2G), programmed cell death (Figure 2H) and inflammatory cytokines expression (Figure S1M, Supporting Information). Moreover, NEU4 overexpression enhanced cellular senescence and DNA damage in TGF‐*β* or H_2_O_2_‐induced HK‐2 cells (Figure 2H and, Figure S1N‐P, Supporting Information), down‐regulated *KI67* mRNA expression in TGF‐*β*‐induced HK‐2 cells (Figure S1P, Supporting Information). Immunofluorescence assay showed that NEU4 overexpression decreased KI67 and increased *γ*H2AX expression in TGF‐*β*‐induced HK‐2 cell (Figure S1Q, Supporting Information).

We confirmed these findings in primary TECs (PTECs) in TGF‐*β* induced cell injury. In particular, knockdown of NEU4 prevented (Figure S2A‐D, Supporting Information), and overexpression of NEU4 (Figure S2E‐G, Supporting Information) promoted TGF‐*β*‐induced PTECs EMT, ECM and cellular senescence associated genes transcription and protein expression.

### 2.3 NEU4 Knockdown by AAV9 Protected Against UUO‐Induced Renal Fibrosis in Mice

**Figure 3:**
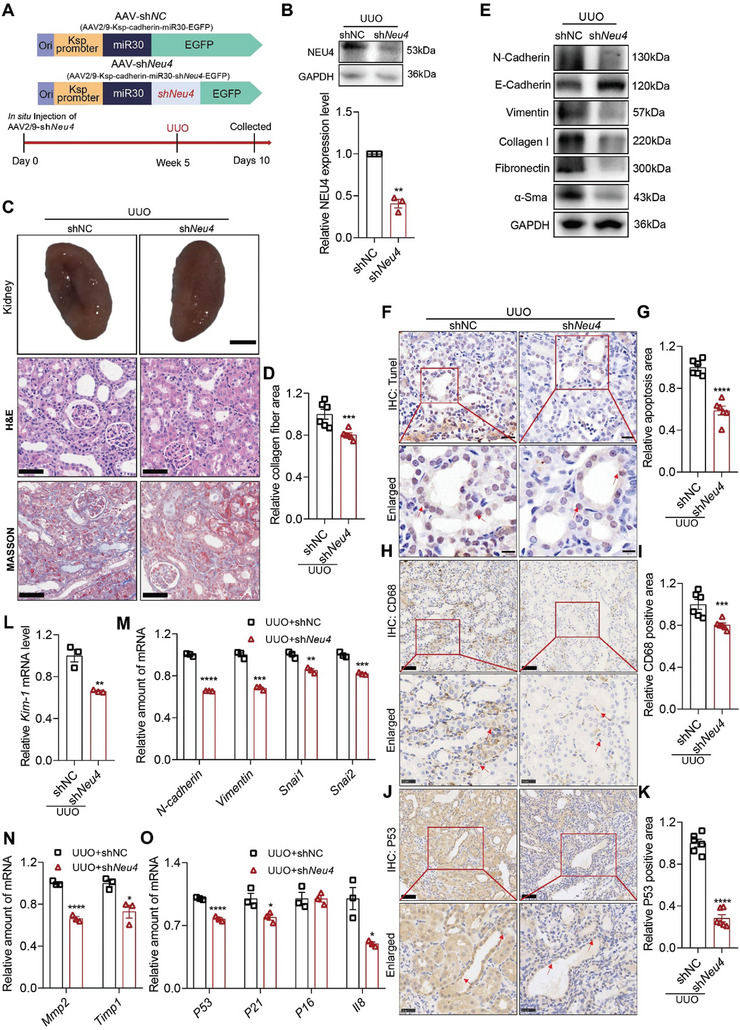
*Neu4* knockdown alleviated UUO‐induced renal fibrosis in mice. A) Scheme of the experimental approach. Mice were in situ injected with adeno‐associated virus (AAV2/9) carrying a coding sequence of mouse sh*Neu4* under kidney‐specific cadherin promoter (AAV2/9‐cadherin‐miR30‐sh*Neu4*‐EGFP, referred as sh*Neu4*) or shNC (AAV2/9‐cadherin‐miR30‐EGFP). Five weeks after injection, the mice were subjected to UUO surgery for 10 days. UUO, unilateral ureteral obstruction. B) Western blot (top panel) and quantification (bottom panel) of the protein expression of NEU4 in left kidney. GAPDH served as loading control, *n* = 3 mice. C,D) The gross appearance of kidneys (*n* = 3 mice. Scale bar, 2 mm), H&E staining and Masson's trichrome staining from left kidneys, and renal interstitial fibrosis scores based on Masson's trichrome staining (D). *n* = 6 mice. Scale bar, 50 µm. E) Western blot of the expression of N‐Cadherin, E‐Cadherin, Vimentin, Collagen I, Fibronectin and *α*‐Sma. GAPDH served as loading control. *n* = 3–5 mice. F,G) Measurement (F) and quantification (G) of apoptosis by TUNEL staining in left kidney. *n* = 6 mice. Scale bar, 20 µm. H–K) Immunohistochemistry staining analysis and quantification of CD68 (H and I) and P53 (J and K) in kidney tissues. *n* = 6 mice. Scale bar, 50 µm. L) *Kim‐1* mRNA level. *n* = 3 mice. M–O) Relative mRNA level of EMT‐associated genes (M), extracellular matrix‐associated genes (N) and senescence‐associated genes (O) were determined by RT‐qPCR, *n* = 3 mice. Error bars represent mean ± SEM. Comparisons between two groups were analyzed by using a two‐tailed Studentʹs *t* test. *p < 0.05, **p < 0.01, ***p < 0.001, ****p < 0.0001 versus the shNC.

To further examine the function of NEU4 in vivo, we deleted *Neu4* in the left kidney of mouse by administration of AAV9‐miR30‐shRNA in situ, followed by a challenge of UUO for 10 days to induce renal fibrosis (**Figure** **3**A,B). *Neu4* knockdown significantly improved renal morphology of the UUO mice (Figure 3C). H&E and Masson staining showed that tubular dilatation, tubular atrophy, and the collagen deposition were attenuated by kidney‐specific knockdown of *Neu4* (Figure 3C,D). The protein expression of EMT markers (N‐cadherin, E‐cadherin, Vimentin) and pro‐fibrotic markers (*α*‐Sma, Fibronectin, Collagen I) were noticeably reversed due to NEU4 knockdown in kidney of UUO‐induced mice (Figure 3E; Figure S3A, Supporting Information). TUNEL staining showed that NEU4 knockdown suppressed apoptosis in TECs (Figure 3F,G). IHC indicated that NEU4 knockdown suppressed macrophage infiltration (Figure 3H,I) and cellular senescence in TECs (Figure 3J,K). Further RT‐qPCR confirmed that NEU4 knockdown dramatically inhibited UUO‐induced gene expressions of *Kim‐1* (Figure 3L), EMT markers (*N‐cadherin*, *Vimentin*, *Snai1*, *Snai2*) (Figure 3M), fibrogenic factors (*Mmp2*, *Timp1*) (Figure 3N), ECM (*Fn1*, *Col1a1*, *Col3a1*, *Col4a1*, *Acta2*) (Figure S3B, Supporting Information), inflammation factor (*Il1β*, *Tgf‐β*) (Figure S3C, Supporting Information) and senescence markers (*P53*, *P21* and *Il8*) (Figure 3O).

### 2.4 NEU4 Overexpression by AAV9 Aggravated UUO‐Induced Renal Fibrosis in Mice

**Figure 4:**
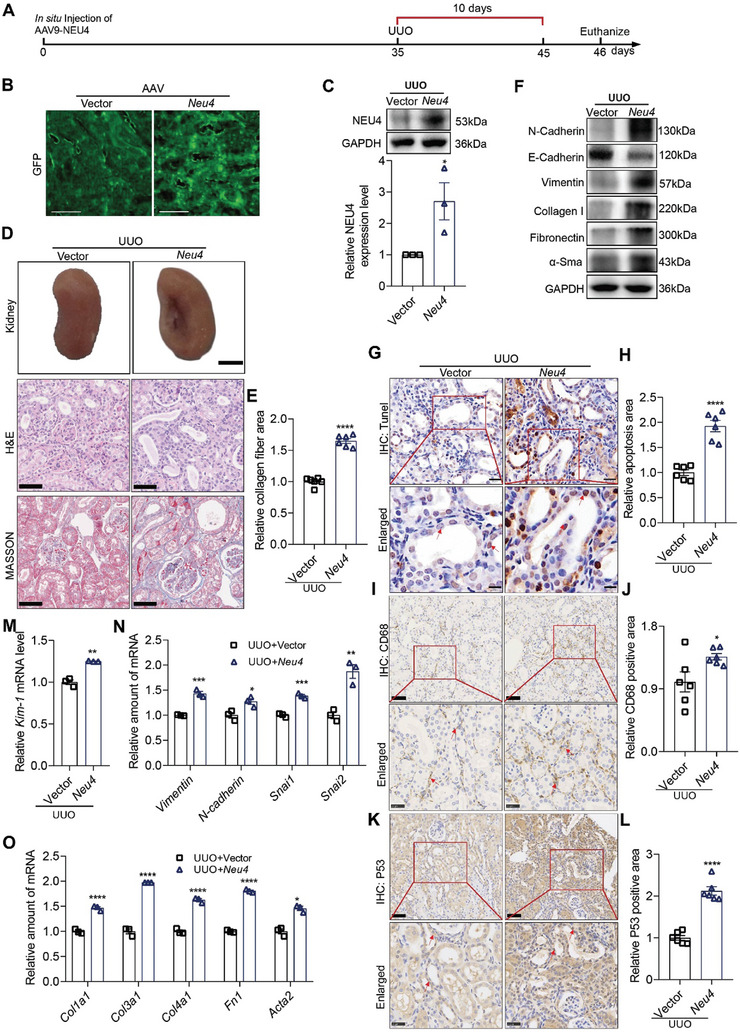
*Neu4* overexpression aggravated UUO‐induced renal fibrosis. A) Schematic diagram of *Neu4* overexpression in mice. The cortex of the kidney in mice was in situ injected with AAV9 encoding GFP‐*Neu4* or scramble. After the injection for 5 weeks, the mice were subjected to UUO surgery for 10 days. B) Immunofluorescence images of GFP in kidney from Vector or *Neu4* overexpression mice. Scale bar, 500 µm. C) Western blot (top panel) and quantification (bottom panel) of the protein expression of NEU4. GAPDH served as loading control, *n* = 3 mice. D,E) The gross appearance of kidneys. *n* = 3 mice. Scale bar, 2 mm. H&E staining and Masson's trichrome staining from left kidneys of UUO mice, and quantification of fibrosis area (D). *n* = 6 mice. H&E staining, scale bar, 50 µm. Masson's trichrome staining, scale bar, 100 µm. F) Western blot of the protein expression of *α*‐Sma, N‐Cadherin, E‐Cadherin, Vimentin, Fibronectin and Collagen I in kidneys. GAPDH served as loading control, *n* = 3–5 mice. G,H) Measurement and quantification of apoptosis by TUNEL staining in left kidney. *n* = 6 mice. Scale bar, 20 µm. I–L) Immunohistochemistry staining analysis and quantification of CD68 (I and J) and P53 (K and L) in kidney tissues. *n* = 6 mice. Scale bar, 50 µm. M) *Kim‐1* mRNA level. *n* = 3 mice. N,O) Relative EMT associated gene (N), and ECM associated gene (O) mRNA level in left kidney. *n* = 3 mice. Error bars represent mean ± SEM. Comparisons between two groups were analyzed by using a two‐tailed Studentʹs *t* test. *p < 0.05, **p < 0.01, ***p < 0.001, ****p < 0.0001 versus the Vector group.

Besides loss‐of‐function, a gain‐of‐function approach was performed using AAV9 encoding NEU4 (NEU4) and AAV9‐vector (Vector). The virus was injected in situ in the cortex of the kidney in mice. The protein expression of NEU4 was significantly increased in the kidneys after injection of AAV9‐NEU4 for 5 weeks (**Figure** **4**A‐C). As expected, NEU4 overexpression exacerbated the UUO‐induced renal shriveled (Figure 4D), tubular dilatation, tubular atrophy, and collagen deposition of the kidneys (Figure 4D,E). The protein expression of EMT markers and pro‐fibrotic markers were aggravated by NEU4 overexpression in kidney of UUO‐induced mice (Figure 4F; Figure S4A, Supporting Information). TUNEL staining showed that NEU4 overexpression aggravated UUO‐induced TECs apoptosis (Figure 4G,H). IHC indicated that NEU4 overexpression aggravated UUO‐induced macrophage infiltration (Figure 4I,J) and cellular senescence (Figure 4K,L) in TECs. Further RT‐qPCR indicated that NEU4 overexpression also augmented UUO‐induced gene expressions of *Kim‐1* (Figure 4M), EMT markers (*N‐cadherin*, *Snai1*, *Snai2*, *Vimentin*) (Figure 4N), fibrogenic factors (*Timp1*, *Mmp2*) (Figure S4B, Supporting Information), inflammation chemokines (*Tgf‐β*, *Ccl2*) (Figure S4C, Supporting Information), ECM (*Fn1*, *Col1a1*, *Col3a1*, *Col4a1*, *Acta2*) (Figure 4O) and senescence markers (*P53* and *P21*) (Figure S4D, Supporting Information).

### 2.5 NEU4 Interacted with Yes‐Associated Protein (YAP)

**Figure 5:**
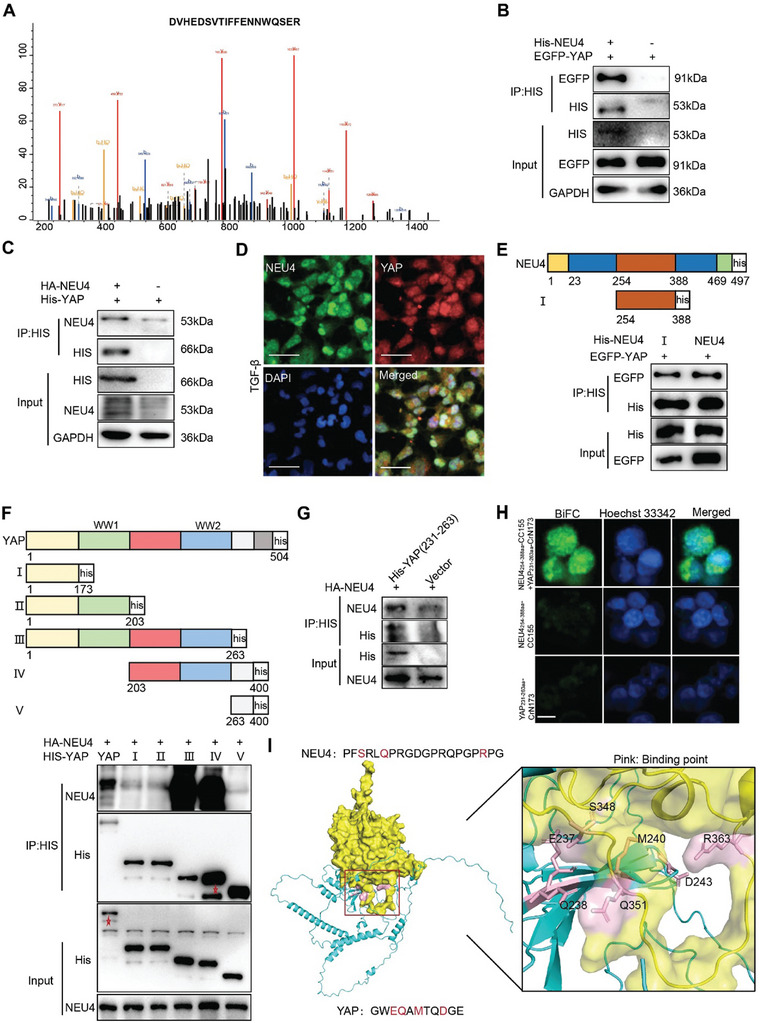
NEU4 interacted with Yes‐associated protein (YAP). A) YAP peptide fragment was precipitated with NEU4 by mass spectrometry (MS). B,C) Western blotting of CoIP of NEU4 and YAP in HK‐2 cells treated with TGF‐*β*. Two independent experiments were performed. D) Colocalization of NEU4 and YAP was analyzed by immunofluorescence in HK‐2 cells stimulated with TGF‐*β*. Scale bar, 100 µm. E) HEK293T cells were co‐transfected with indicated EGFP‐YAP and His‐NEU4 deletion mutants’ plasmid. Cell lysates were IP with His antibody. F) HEK293T cells were co‐transfected with indicated HA‐NEU4 and His‐YAP deletion mutants plasmid. Cell lysates were IP with His antibody. G) Coimmunoprecipitation of NEU4 and His‐YAP _231–263aa_ in HEK293T cells. H) BiFC signals were detected in 293T cells. Representative fluorescence images of 293T cells co‐expression of pBiFC‐NEU4_254‐388aa_‐CC155 and pBiFC‐YAP_231‐263aa_‐CrN173. For the control group, cells were transfected with pBiFC‐NEU4_254‐388aa_‐CC155 or pBiFC‐YAP_231‐263aa_‐CrN173 plasmids. Scale bar, 10 µm. I) Molecular docking of 3D structures shows the interaction of NEU4 domain (yellow) with YAP domain (blue).

To investigate the underlying mechanism by which NEU4 promotes renal fibrosis, we performed immunoprecipitation combined with mass spectrometry analysis (IP‐MS) in protein lysates of TGF‐*β*‐induced HK‐2 cells. We identified YAP, a member of the hippo signaling pathway,^[^ ^28^ ^]^ which has been shown to play an essential role in kidney fibrosis, as a potential NEU4‐interacting protein (**Figure** **5**A). Co‐immunoprecipitation (Co‐IP) assay showed that NEU4 binds to YAP in the HK‐2 cells (Figure 5B,C). NEU4 and YAP were co‐localized in the nucleus of TGF‐*β*‐stimulated HK‐2 cells (Figure 5D). We further identified the interacting domains of NEU4 and YAP using truncation and deletion mutants. The results showed that the domain of NEU4_254‐388aa_ and the WW2 domain of YAP_231‐263aa_ were the interacting region (Figure 5E,F). Furthermore, we constructed a His‐Tag plasmid containing 231–263 amino acid sequences, and observed that NEU4 interacted with this domain of YAP by CoIP (Figure 5G). To verify the co‐localization of these interactions, we used bimolecular fluorescence complementation (BiFC) to detect the direct interaction between NEU4 and YAP in 293T cells. The results showed that fluorescent signals of NEU4_254‐388aa_ and YAP_231‐263aa_ interaction were mainly co‐localized with nuclear (Figure 5H). The docking simulation data demonstrated that amino acids in the WW2 domain of YAP and the domain of NEU4 are responsible for their interaction (Figure 5I).

### 2.6 NEU4 Inhibited Activation of YAP

**Figure 6:**
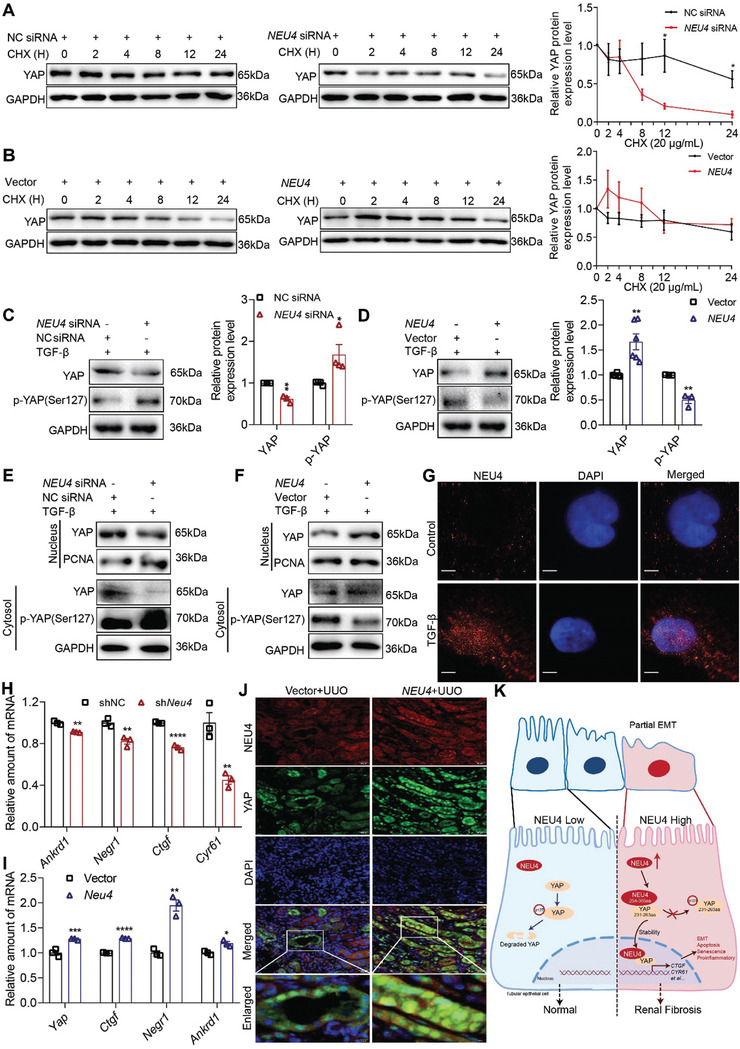
NEU4 inhibited activation of YAP. A,B) Western blot (left panel) and quantification (right panel) of the protein expression of YAP in HK‐2 cells treatment with TGF‐*β* for 24 h, and then with cycloheximide (CHX, 20 µg mL^−1^) for the indicated periods of time (0, 2, 4, 8, 12, 24 h) after transfection either with *NEU4* siRNA (A) or *NEU4* overexpression plasmid (B). GAPDH served as loading control. *n* = 3 biologically independent samples. C,D) Western blot (left panel) and quantification (right panel) of the protein expression of YAP and phosphorylation of YAP in HK‐2 cells treatment with TGF‐*β* 24 h after transfection either *NEU4* siRNA (C) or *NEU4* overexpression plasmid (D). GAPDH served as loading control. *n* = 3–6 samples. E,F) Western blot of YAP and phosphorylation of YAP in nuclear and cytosol of HK‐2 cells following 24 h after treatment with TGF‐*β* subsequent to transfection with either *NEU4* siRNA (E) or *NEU4* overexpression plasmid (F). G) Localization of NEU4 was analyzed by immunofluorescence in HK‐2 cells stimulated with TGF‐*β* for 24 h. Scale bar, 5 µm. H,I) mRNA abundance of Yap target genes in left kidney tissue of UUO‐mice treated with sh*Neu4* (H) or *Neu4* overexpression plasmid (I). *n* = 3 mice. J) Colocalization of NEU4 and YAP was analyzed by immunofluorescence in left kidneys. Scale bar, 50 µm. K) The proposed mechanisms of NEU4‐mediated renal fibrosis. Error bars represent mean ± SEM. Comparisons between two groups were analyzed by using a two‐tailed Studentʹs t test. *p < 0.05, **p < 0.01, ***p < 0.001, ****p < 0.0001 versus the NC siRNA, shNC or Vector group.

We next determined whether there was a regulatory relationship between NEU4 and YAP. To investigate the effects of NEU4‐YAP interaction on YAP, we measured the stability of YAP in the presence or absence of NEU4. NEU4 knockdown promoted YAP degradation, whereas NEU4 overexpression inhibited YAP degradation (**Figure** **6**A,B), suggesting that NEU4 interacted with and stabilized YAP. YAP is capable of translocating into the nucleus to facilitate the transcription of downstream targets, while phosphorylated YAP at ser127 can disrupt this process and prevent its entry into the nucleus.^[^ ^29^, ^30^, ^31^ ^]^

To this end, we examined the expression of YAP and phosphorylation of YAP in HK‐2, PTEC cells and mice through both gain‐of‐function and loss‐of‐function experiments involving NEU4. We observed that knockdown of NEU4 resulted in an elevation of phosphorylation of YAP levels but a decrease in total YAP level, suggesting that YAP was activated (Figure 6C; Figure S5A‐D, Supporting Information). Conversely, overexpression of NEU4 led to a reduction in phosphorylation of YAP level, while increasing the total YAP protein level (Figure 6D; Figure S6A–D, Supporting Information).

Next, the nuclear localization of YAP was determined in response to NEU4 gain or loss of function. Our findings demonstrated that NEU4 knockdown resulted in a downregulation of YAP levels within the nuclei of HK‐2 (Figure 6E; Figure S5A, Supporting Information) and PTEC cells (Figure S5C, Supporting Information), an upregulation of phosphorylation of YAP within the cytosol of HK‐2 cells (Figure 6E), whereas overexpression of NEU4 led to an augmentation in nuclear YAP levels and a reduction in cytoplasmic phosphorylation of YAP (Figure 6F; Figure S6A,C, Supporting Information). The immunofluorescence results showed that NEU4 was able to translocate into the nucleus in response to TGF‐*β* stimulation (Figure 6G).

We next determined the effect of NEU4 on YAP target genes (*Cyr61*, *Ankrd1*, *Ctgf* and *Negr1*). RT‐qPCR showed that NEU4 knockdown inhibited YAP target genes expression (Figure 6H) in UUO mice. In contrast, NEU4 overexpression promoted YAP target genes expression in *vivo* (Figure 6I). The luciferase reporter assays found that NEU4 knockdown suppressed the activated effect of YAP on *CTGF*, *NEGR1*, *CYR61* and *ANKRD1* relative luciferase activity in 293T (Figure S6E‐H, Supporting Information), whereas NEU4 overexpression reversed the effect of YAP (Figure S6I‐L, Supporting Information). The immunofluorescence results confirmed that NEU4 was able to translocate into the nucleus and co‐localize with YAP in kidney of UUO mice (Figure 6J). Collectively, these results indicate that NEU4 interacts with YAP, inhibits YAP phosphorylation, promotes YAP transfer to the nucleus, and then activates YAP target genes expression, thereby contributing to renal fibrosis (Figure 6K).

### 2.7 3,5,6,7,8,3ʹ,4ʹ‐Heptamethoxyflavone (HMF) was Screened as a Novel Inhibitor of NEU4

**Figure 7:**
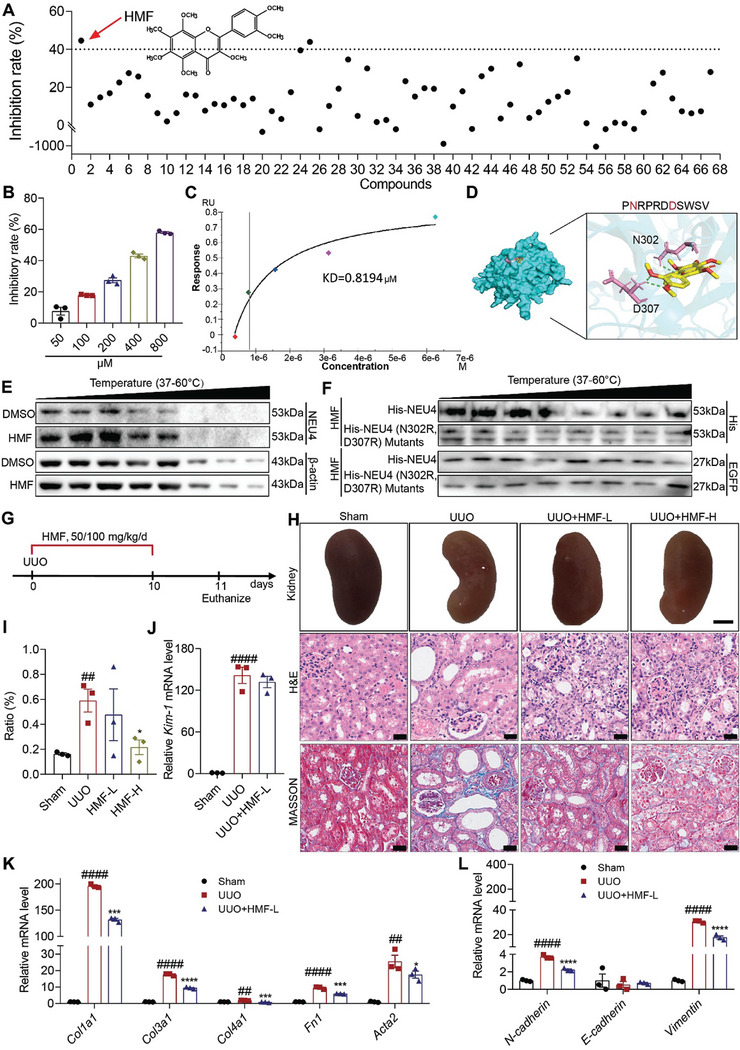
3,5,6,7,8,3ʹ,4ʹ‐Heptamethoxyflavone (HMF) was screened as a novel inhibitor of NEU4. A) Inhibitory rate of 67 compounds on NEU4 enzyme activity. The dashed line represents a 40% inhibitory rate. B) The inhibitory rate of HMF on NEU4 enzyme activity at different concentrations. C) SPR assay showed the interaction of HMF with NEU4. D) Molecular docking analysis of the interaction between HMF and NEU4. E) CETSA assays confirmed the binding of HMF to NEU4 in HK‐2 cells. *β*‐actin was used as the internal control. F) CETSA assays confirmed the binding of HMF to NEU4 or NEU4 (N302R, D307R) Mutants in HK‐2 cells treatment with TGF‐*β*. HK‐2 cells treated with HMF 24 h after transfection with EGFP and NEU4 or NEU4 mutants. EGFP was used as the internal control. G) The schematic of experimental design. Vehicle, or HMF (50 or 100 mg kg^−1^/day) was administrated to UUO mice by gastric irrigation once daily for 10 days. H,I) The gross appearance of kidneys (*n* = 3 mice. Scale bar, 2 mm), H&E staining and Masson's trichrome staining from left kidneys of UUO mice, and renal interstitial fibrosis scores based on Masson's trichrome staining (I), *n* = 3 mice. Scale bar, 50 µm. J) *Kim‐1* mRNA level. *n =* 3 mice. K,L) Relative mRNA levels of ECM associated genes (K) and EMT‐associated genes (L) were determined by RT‐qPCR. *n* = 3 mice. Error bars represent mean ± SEM. Comparisons those among three or more groups by using one‐way analysis of variance (ANOVA) followed by Dunnettʹs post hoc tests. *p < 0.05, ***p < 0.001, ****p < 0.0001 versus the UUO group, ^##^ *p* < 0.01, ^####^ *p* < 0.0001 versus the Sham group.

To screen potential compounds that can inhibit mammalian NEU4, the enzyme activity of 67 natural products derived from medicinal plants was assessed (**Figure** **7**A; Table S2, Supporting Information). HMF emerged as the most potent inhibitor of NEU4 enzyme activity, demonstrating a dose‐dependent suppression, as illustrated in Figure 7B. Meanwhile, we detected the inhibitory rate of DANA (2‐deoxy‐2,3‐dehydro‐N‐acetylneuraminic acid), a NEU4 inhibitor,^[^ ^32^ ^]^ on NEU4 enzyme activity, which was 53.3 (Table S2, Supporting Information). The results showed that the inhibitory rate of HMF was very close to that of DANA. To further investigate the interaction between HMF and NEU4, a surface plasmon resonance assay (SPR) was conducted to evaluate the binding affinity of HMF with human recombinant NEU4. The results indicated a strong binding affinity of HMF with human recombinant NEU4, with an estimated equilibrium dissociation constant of 0.819 µm (Figure 7C). The interaction between NEU4 and HMF was subsequently confirmed using SPR in conjunction with mass spectrometry (Figure S7A,B, Supporting Information).

Following this, we performed molecular docking utilizing the crystal structure of NEU4 (Figure 7D) to explore the potential binding modes of HMF with NEU4. The findings indicate that HMF forms favorable hydrophobic interactions with NEU4 residues, particularly D307 and N302, displaying the lowest binding energy of −8.51 kcal mol^−1^ (Figure 7D). Substituting the HMF binding sites on NEU4 (D307R, N302R) resulted in that HMF had no impact on the thermal stability of NEU4, providing additional evidence for NEU4 as a direct binding target of HMF (Figure 7E,F). CoIP and immunofluorescence experiments (Figure S8A‐C, Supporting Information) revealed that HMF notably hindered the interaction between NEU4 and YAP. HMF and DANA inhibited the expression of NEU4 (Figure S9, A and B supporting information), with HMF showing a stronger effect. Furthermore, HMF inhibited YAP expression, increased phosphorylation of YAP, and impeded YAP translocation to the nucleus (Figure S9C‐E, Supporting Information) in TGF‐*β*‐induced HK‐2 cells.

Furthermore, without discernible toxicity, HMF demonstrated protective effects on HK‐2 cells (Figure S10A,B, Supporting Information). The treatment with HMF resulted in a significant reversal of expression of genes related to EMT, kidney injury molecule, fibrogenic factors (Figure S10C,D, Supporting Information), cell apoptosis (Figure S11A,B, Supporting Information), senescence (Figure S11C,D Supporting Information), and pro‐inflammatory cytokines (Figure S12, Supporting Information) in HK‐2 cells. These observations were further validated in PTECs (Figures S13 and S14, Supporting Information).

Subsequently, we investigated the protective effects of HMF against renal injury in mouse models. Of note, oral administration of HMF (50 or 100 mg kg^−1^/d) alleviated UUO‐induced renal fibrosis (Figure 7G), with significantly improved kidney morphology (Figure 7H). H&E and Masson staining demonstrated that HMF attenuated UUO‐induced renal injury and renal fibrosis in dose‐dependent manner (Figure 7I). Western blots showed that HMF markedly reversed UUO‐induced expression of renal fibrosis‐associated proteins (Figure S15A, Supporting Information). Although low doses of HMF treatment did not significantly inhibited *Kim‐1* expression (Figure 7J), it markedly suppressed ECM‐associated genes (*Fn1*, *Acta2*, *Col1a1*, *Col3a1* and *Col4a1*) (Figure 7K), EMT progression‐related genes (*N‐cadherin*, *Vimentin*) (Figure 7L), chemokines (*Tgf‐β*, *Ccl2*) (Figure S15B, Supporting Information), matrix metalloproteinase (*Fsp1*, *Mmp2*, *Mmp7*, *Mmp9*, *Mmp13* and *Timp1*) (Figure S15C, Supporting Information), proinflammatory cytokine production (*Tnfα*, *Il6*, and *Il1β*) (Figure S15D, Supporting Information), senescence markers (*P21*, *P16* and *P53*) (Figure S15E, Supporting Information) and macrophage infiltration (Figure S15F, Supporting Information). The protective effects of HMF from renal injury were replicated in FA induced mouse model (Figures S16 and S17, Supporting Information). These results suggest that HMF exerts a significant renal protective effect.

Moreover, HMF inhibited the expression of NEU4 and YAP, promoted the expression of phosphorylation of YAP in kidneys from UUO (Figure S18A,B, Supporting Information) and FA mice (Figure S18C,D, Supporting Information). HMF inhibited the interaction between NEU4 and YAP in UUO mice (Figure S18E, Supporting Information).

### 2.8 Targeting NEU4 by HMF Attenuated Kidney Fibrosis

To determine whether the effect of HMF on renal fibrosis is depended on NEU4, we employed a NEU4 knockdown model. In NEU4‐knockdown HK‐2 and PTEC cells, the administration of HMF did not result in additional reduction in cell senescent (Figure S19A, Supporting Information), expression of EMT progression‐related proteins (Figures S19B and S20, Supporting Information), fibrogenic factors (Figures S19B and S20, Supporting Information) as well as YAP and phosphorylation of YAP (Figure S21, Supporting Information) in response to TGF‐*β* stimulation. On the contrary, in NEU4‐overexpressing HK‐2 cells, HMF reduced the expression of EMT progression‐related proteins (Figure S22A, Supporting Information), cell senescent (Figure S22B, Supporting Information), and YAP (Figure S22C, Supporting Information) in response to TGF‐*β* stimulation.

**Figure 8:**
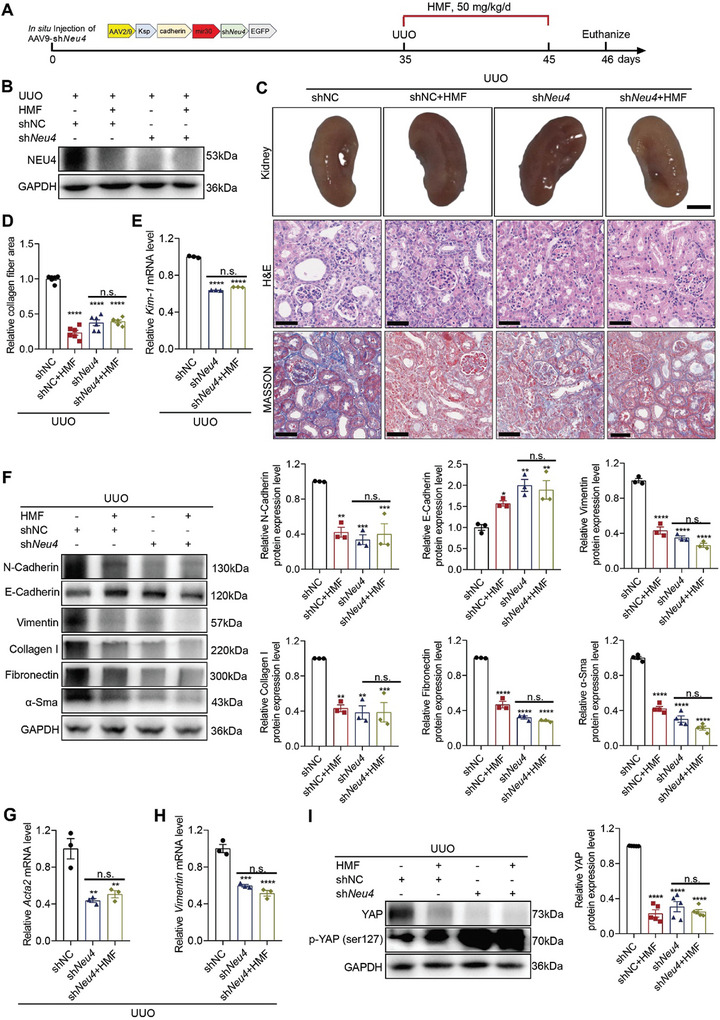
*Neu4* knockdown relieved the anti‐fibrotic effects of HMF in UUO model. A) Schematic diagram of experimental approach. Mice were in situ injected with AAV2/9 encoding shNC or sh*Neu4*. Five weeks after injection, the mice were subjected to UUO surgery, then vehicle or HMF (50 mg kg^−1^/day) was administrated to mice by gastric irrigation once daily for 10 days. B) Western blot of NEU4 in kidney. GAPDH served as loading control. C,D) Picture of left kidneys of mice with different treatments, H&E staining and Masson's trichrome staining from left kidneys of UUO mice and renal interstitial fibrosis scores based on Masson's trichrome staining (D). *n* = 6 mice. Scale bar, 50 µm. E) *Kim‐1* mRNA level. *n* = 3 mice. F) Western blot (left panel) and quantification (right panel) of the protein expression of N‐Cadherin, E‐Cadherin, Vimentin, Collagen I, Fibronectin and *α*‐Sma. GAPDH served as loading control. *n* = 3–4 mice. G,H) *Acta2* (G) and *Vimentin* (H) mRNA level. *n* = 3 mice. I) Western blot (left panel) and quantification (right panel) of the protein expression of YAP and phosphorylation of YAP in kidneys. GAPDH served as loading control. *n* = 5 mice. Error bars represent mean ± SEM. Comparisons those among three or more groups by using one‐way analysis of variance (ANOVA) followed by Dunnettʹs post hoc tests. *p < 0.05, **p < 0.01, ***p < 0.001, ****p < 0.0001 versus the shNC group. n.s.: no significance.

In NEU4‐knockdown mice (**Figure** **8**A,B), treatment of HMF (50 mg kg^−1^) failed to further reduce kidney morphology (Figure 8C), renal injury and renal fibrosis following UUO stimulation (Figure 8C,D). Consistently, HMF did not exhibit additional inhibitory effects on UUO‐induced *Kim‐1* expression (Figure 8E), EMT (Figure 8F‐H; Figure S23, Supporting Information), and collagen production (Figure 8F‐H) in NEU4‐knockdown mice. Furthermore, the inactivation effects on YAP, and the activation effects on phosphorylation of YAP were not further enhanced by HMF treatment after NEU4 knockdown in vivo (Figure 8I). Conversely, in NEU4‐overexpressing mice, HMF effectively restored the capacity to mitigate UUO‐induced renal injury and renal fibrosis (Figure S24A‐C, Supporting Information). HMF also decreased the expression of *α*‐Sma, N‐Cadherin, Vimentin, Fibronectin, Collagen I and YAP, increased the expression of E‐Cadherin and phosphorylation of YAP (Figure S24D,I, Supporting Information).

Meanwhile, HMF suppressed the expression of *Kim‐1*, EMT progression‐related genes (*N‐cadherin*, *Vimentin*, *Tgf‐β*, *Snai1*, *Snai2*), ECM‐associated genes (*Fn1*, *Col1a1*, *Col3a1*, and *Col4a1*), *Yap* and its targets *Negr1* and matrix metalloproteinase (*Mmp2*, *Mmp9*) after NEU4 overexpression in vivo (Figure S24E‐H,J, Supporting Information). These findings suggest that NEU4 is essential for the renal protective effects of HMF.

## 3 Discussion

In this study, we described the presence of NEU4 in TECs and revealed the significant involvement of NEU4 in renal fibrosis through the findings of in vivo, in vitro, and pharmacological investigations. The major findings of this work include: i) NEU4 was significantly elevated in TECs of fibrotic kidneys from human and mice; ii) NEU4 was identified as a promotor of renal fibrosis using adeno‐associated virus; iii) mechanistically, The 254–388 amino acid of NEU4 was found to interact with Yap within the amino acid region 231–263, resulting in the translocation of Yap into the nucleus and subsequent upregulation of yap‐targeted genes (*CTGF)*; and iv) HMF was screened as a novel inhibitor of NEU4 and effectively alleviated renal fibrosis.

The expression of NEU4 in various diseases remains poorly understood, especially in renal fibrosis. In this work, we shown that NEU4 was significantly increased in patients with renal fibrosis, in mice undergoing unilateral ureteral obstruction (UUO) or receiving folic acid, and in human tubular epithelial HK‐2 cells or primary tubular epithelial cells (PTECs) stimulated with TGF‐*β*. Our findings aligned with prior research that showed an increase in NEU4 levels in the lungs of patients with pulmonary fibrosis.^[^ ^33^ ^]^

To investigate the role of NEU4 in the development of renal fibrosis, we used AAV to generate kidney‐specific NEU4 knockdown/overexpression mice by in situ injection. Kidney‐specific NEU4 knockdown was sufficient to protect kidney from UUO‐ or folic acid‐induced renal fibrosis in mice and TGF‐*β*‐induced injury in TECs. These altered phenotypes and molecular markers included EMT, apoptosis, cell senescence, and fibrosis‐associated genes. Conversely, NEU4 overexpression aggravated fibrosis‐associated phenotypes in vitro and in vivo. Although the influence of neuraminidases in other renal cell types such as glomerular podocytes and fibroblasts cannot be totally excluded in renal fibrosis, this study illuminates the significant role of tubular NEU4 in promoting renal fibrosis.

Over the last decades, NEU4 has attracted much attention in cancer, metabolic disease, neurogenesis, and apoptosis.^[^ ^34^, ^35^, ^36^, ^37^, ^38^ ^]^ In this work, we explored the role of NEU4 in renal fibrosis. Apart from NEU4, the human neuraminidase family comprised three other isoforms, namely NEU1, NEU2, and NEU3. Previous studies on NEU2 and NEU3 have primarily concentrated on their implications in cancers, intestinal and pulmonary disorders, as well as neurological conditions.^[^ ^39^, ^40^, ^41^ ^]^ We and others have demonstrated the contribution of NEU1 in cardiovascular diseases and renal injury.^[^ ^4^, ^42^, ^43^ ^]^ It is noteworthy that NEU4 and NEU1 exhibit similar functionality in the context of renal fibrosis, despite disparities in their protein sequences, subcellular localization, and enzymatic substrates. The similar functions of NEU4 and NEU1 in kidney injury necessitate deeper investigation in future research endeavors.

NEU4, like NEU1, was thought to be localized in lysosomes, where it plays a role in the degradation of the sialoglycoconjugate by eliminating the terminal sialic acid.^[^ ^34^, ^44^ ^]^ Besides lysosomes, recent findings have indicated the localization of NEU4 within mitochondria, endoplasmic reticulum, and the nucleus.^[^ ^24^, ^25^, ^45^ ^]^ In this work, we have demonstrated that NEU4 was activated in cytoplasm and translocated into the nucleus in renal fibrosis. Co‐immunoprecipitation and liquid chromatography‐tandem mass spectrometry identified a transcriptional coactivator factor YAP that strongly binds to NEU4. YAP, an important coactivator of the Hippo pathway, regulates the transcription of targeted genes including CTGF, and is closely associated with the development of CKD.^[^ ^46^, ^47^, ^48^, ^49^ ^]^ Two critical domains that regulate the activity of YAP are WW1 and WW2.^[^ ^50^, ^51^ ^]^ Our work showed that 254–388 amino acid region of NEU4 was directly bound to the WW2 domain located at 231–263 region. It is thus presumed that the binding of NEU4 with the WW2 domain inhibits the phosphorylation of YAP, and promotes YAP translocation from the cytoplasm to the nucleus. However, the exact molecular basis remains unknown and depends on the characterization of the crystal structure of NEU4‐YAP complex.

The development of compounds that interact with and inhibit NEU4 remains a challenge due to limited research and the lack of a crystal structure. We employed a “Enzyme activity screening‐Virtual docking‐SPR” strategy to discover potential NEU4‐inhibiting compounds derived from medicinal plants. HMF, isolated from the peel of Citrus plants, exhibited the strongest inhibitory activity of NEU4. HMF inhibited NEU4 protein expression and suppressed the interaction between NEU4 and YAP. HMF demonstrated renal protective potential in mouse models with UUO‐induced and FA‐induced kidney damage. To our knowledge, this is the first discovery that HMF can ameliorate renal fibrosis. HMF has been reported to display a broad range of biological and pharmacological effects, such as potent anti‐inflammatory, antitumor and neuroprotective effects.^[^ ^52^, ^53^, ^54^ ^]^ Our previous research suggested that HMF can target FKBP38 and mTOR/P70S6K/SREBPs pathway to alleviate HFD‐induced hyperlipidemia.^[^ ^55^ ^]^ Small‐molecule drugs have the potential to interact with multiple targets and exhibit diverse pharmacological activities. It is probable that HMF suppressed renal fibrosis by affecting complex, distinct, and interconnected signaling pathways. Here, we provide evidence that NEU4 is the direct interacting target of HMF, and NEU4 is essential for the renal protective effects of this compound.

This study has some limitations. First, we have shown that 254–388 amino acid of NEU4 interacted with the WW2 domain of YAP, but the specific binding sites for NEU4 were not characterized because of the limited availability of information on this protein. Second, in addition to activating the YAP signaling pathway, NEU4 may also be participated in other signaling pathways that remain to be explored. Thirdly, additional investigation is required to elucidate the potential involvement of alternative signaling pathways or targets in the therapeutic effects of HMF against renal fibrosis, as well as to unravel the intricate interplay of mechanisms.

In conclusion, this study identifies a promotor role for NEU4 in renal fibrosis and proposes a potential therapeutic strategy involving the targeting NEU4 for the treatment of chronic kidney disease.

## 4 Experimental Section

### Human Kidney Samples

This study complied with the ethical guidelines of the 1975 Declaration of Helsinki. Human kidney biopsy samples were obtained from Xiangya Hospital. This study was approved by the Ethical Review Committees of Xiangya Hospital (LYEC2024‐0138). Detailed information on participants is provided in Table S1 (Supporting Information).

### Mice

Mice were maintained in the center for Experimental Animals at China Pharmaceutical University, Nanjing, China. All procedures involving experimental animals were performed following protocols approved by the Committee for Animal Research of China Pharmaceutical University (permit number: 2023‐01‐007) and conformed to the Guide for the Care and Use of Laboratory Animals. C57BL/6J male mice aged 8–10 weeks were used. All animals were maintained under constant humidity and temperature at standard facilities under specific pathogen‐free conditions with free access to water and chow (Xietong Organism, China). Mice were euthanized by cervical dislocation.

### Animal Models

For UUO experiments, was performed by permanent ligation of the left ureter with 4‐0 silk. Ureter‐ligated kidneys and contralateral kidneys (CLs), were collected 10 days after surgery. For HMF experiments, mice were once daily oral gavage with vehicle, 50 or 100 mg kg^−1^ HMF for 10 days.

FA–induced renal fibrosis was conducted by single intraperitoneal injection of 250 mg kg^−1^ folic acid (Sigma–Aldrich, 7876, USA) dissolved in 0.3 m sodium bicarbonate, and mice were sacrificed 28 days after FA treatment. Control mice injected with sodium bicarbonate (i.p). For HMF experiments, mice were once daily oral gavage with vehicle, 50 or 100 mg kg^−1^ HMF for 28 days.

### Cell Culture and Treatments

Human proximal tubular epithelial cells (HK‐2) were obtained from the Cell Bank of the Chinese Academy of Sciences (Shanghai, China) and cultured in DMEM/F12 (KeyGEN BioTECH, China) supplemented with 10% fetal bovine serum (FBS, Gibco) and 1% penicillin/streptomycin (KeyGEN BioTECH, China).

HK‐2 cells were treated with 10 ng mL^−1^ recombinant human TGF‐*β*1 protein (PeproTech, USA), or 10 µg mL^−1^ LPS (L2630, Sigma, USA) with or without HMF for 24 h.

HK‐2 cells were treated with or without HMF for 24 h, then treated with 400 µm H_2_O_2_ for 6 h.

293T cells were obtained from the Cell Bank of the Chinese Academy of Sciences and cultured in DMEM (KeyGEN BioTECH, China) supplemented with 10% fetal bovine serum and 1% penicillin/streptomycin.

### Isolation of Primary Mouse Renal Tubular Epithelial Cells (PTECs)

Kidneys from mice (3‐ to 5‐week‐old males) were collected after euthanization and minced into pieces of ≈1 mm^3^. These pieces were digested with 10 mL PBS containing 2 mg mL^−1^ collagenase II (Thermo Fisher Scientific, USA) for 15 min at 37 °C with gentle stirring, and supernatants were sieved through a 100 and 40 µm nylon mesh. After centrifugation for 3 min at 500 g, the pellet was resuspended in DMEM/F12 and seeded in 10 cm culture dishes. Cells were cultured in DMEM/F12 supplemented with 10% FBS, 50 units per mL penicillin, and 50 µg mL^−1^ streptomycin at 37 °C and 5% CO_2_. Cells were used between days 7 and 10 of culture. PTECs were treated with 10 ng mL^−1^ recombinant human TGF‐*β*1 protein.

### Senescence‐Associated *β*‐Galactosidase (SA‐*β*‐Galactosidase) Detection

SA‐*β*‐galactosidase activity in cultured cells was detected histochemically using the Senescence Detection Kit (Beyotime Biotechnology, China). In brief, culture medium was removed, the cultured cells were washed with phosphate‐buffered saline (PBS), and then fixed with Fixative Solution at 25 °C for 15 min. After rinsing with PBS, the cells were stained overnight with the staining solution at 37 °C. After incubation, the stained cells were observed under a fluorescence microscopy (Nikon, Japan), and quantified by Image J (National Institutes of Health, USA).

### Tunel Staining

TUNEL staining was employed to determine apoptotic cells. Cells were fixed with 4% paraformaldehyde (PFA) for 30 min, stained with TUNEL BrightGreen Apoptosis Detection Kit (A112, Vazyme, China) after wash for 30 min at 37 °C. Images were captured with fluorescence microscopy, and quantified by Image J.

### Histological Analysis

Mouse kidneys were fixed 4% PFA, embedded in paraffin, and sectioned in 5 µm thickness for Masson's trichrome and haematoxylin and eosin (H&E). Sections were examined under digital pathological section scanner (NanoZoomer 2.0 RS, Hamamatsu, Japan). Measurement of the fibrotic area was quantified with Image J software.

### Assessment of Kidney Function

Animal kidney function was determined by analyzing the indicators serum creatinine by the Creatinine (Cr) Assay kit (Nanjing Jiancheng Bioengineering Institute, China) according to the manufacturers instructions.

### Microinjection of Adeno‐Associated Virus into Mouse Kidney

To overexpress NEU4 in vivo, it designed an adeno‐associated virus (AAV) serotype 9 vector encoding a green fluorescent protein reporter together with either plasmid targeting *Neu4* (AAV‐*Neu4*) or an empty vector (AAV‐Plasmid) in the kidney. AAV encoding *Neu4* (1 × 10^12^ vector genomes/mL; 10 µL) or empty vector were administered to mice at least 6 distributed points in the cortex of the kidney. Kidneys were collected 35 days after infection, and immunoblotting was used to measure overexpressed efficiency.

The cortex of the kidney in mice was injected in situ with one single dose of 60 µL of AAV2/9‐Ksp‐cadherin‐mir30‐m‐EGFP‐*Neu4* short hairpin RNA (shRNA) virus suspension (virus titer>10^12^) or AAV2/9‐Ksp‐cadherin‐mir30‐m–EGFP‐control shRNA (AAV2/9‐NC shRNA) (Hanheng Biotechnology, China). Kidneys were collected 35 days after infection, and immunoblotting was used to measure knockdown efficiency. The shRNA oligo sequences were as follows: NC shRNA: 5ʹ‐UUCUCCGAACGUGUCACGUTT‐3ʹ, 3ʹ‐ACGUGACACGUUCGGAGAAT T‐5ʹ; *Neu4* shRNA: 5ʹ CAGAGGTCTTCTTGAACGT‐3ʹ, 3ʹ‐ACGTTCAAGAAGACCTCTG‐5ʹ.

### Western Blots

Cells or a quarter piece of each kidney sample were homogenized and lysed in RIPA lysis buffer (P0013B, P0013D, Beyotime Biotechnology, China). containing complete Protease Inhibitor Cocktail (Roche Diagnostics, Switzerland) and phosphatase inhibitor (Roche, Switzerland). The nuclear and cytoplasmic proteins were isolated with a commercial kit (P0027, Beyotime Biotechnology, China). Protein extracts were separated on 8–10% SDS‐polyacrylamide gels and transferred onto nitrocellulose blotting membranes. Membranes were blocked by incubation for 1 h with 5% nonfat milk and blotted overnight at 4 °C with the primary antibodies. After incubation with the corresponding secondary antibodies, membranes were imaged with Tanon‐5200 Chemiluminescent Imaging System (Tanon, Shanghai, China). The following antibodies were used: PCNA (1:2000, 10205‐2‐AP), E‐cadherin (1:1000, 20874‐1‐AP), NEU4 (1:1000, 12995‐1‐AP) and GAPDH (1:5000, 60004‐1‐Ig) (Proteintech, China); COL1A1 (1:1000, sc‐293182), N‐cadherin (1:1000, sc‐59987), YAP (1:1000, sc‐376830), *β*‐actin (1:1000, sc‐81178) and Vimentin (1:1000, sc‐6260) (Santa Cruz Biotechnology, USA); Phospho‐YAP (1:1000, Ser127) (D9W2I), TGF‐*β* (1:1000, 3709S) and *α*‐SMA (1:1000, 19245S) (Cell Signaling Technology, USA); YAP (1:1000, A21216), Phospho‐YAP (1:1000, Ser127, AP0489) and Fibronectin (1:1000, A12932) (Abclonal, China); anti‐rabbit or mouse secondary antibodies (1:2500, ZB‐2301, ZB‐2305, ZSGB‐BIO, Beijing, China).

### Immunohistochemistry Analysis

Embedded kidney samples were sliced in 5 µm thickness, and then were deparaffinized and rehydrated. Slides were then incubated the tissue sections with 5% BSA and treated with the primary antibodies. After washing, slides were incubated with goat anti‐rabbit HRP secondary antibody. The nuclei were counter‐stained with hematoxylin. Images were captured with digital pathological section scanner (NanoZoomer 2.0 RS, Hamamatsu, Japan), and quantified by Image Pro Plus 6.0 (Media Cybernetics, USA). The following antibodies were used: NEU4 (1:100, 12995‐1‐AP, Proteintech, China); NEU4 (1:100, F40623‐0.08 mL, NSJ Bio, USA); CD68 (1:200, 97778, Cell Signaling Technology, USA); P53 (TA0879F, abmart, China).

For the kidney tissues Tunel staining, sections were incubated at TUNEL Apoptosis Detection Kit (KGA702, KeyGEN BioTECH, China). The nuclei were counter‐stained with hematoxylin. Samples were analyzed, and pictures were taken using digital pathological section scanner (NanoZoomer 2.0 RS, Hamamatsu, Japan), and quantified by Image Pro Plus 6.0 (Media Cybernetics, USA).

### Immunoprecipitation

Cells were collected and lysed in IP‐lysis buffer (P0013J, Beyotime Biotechnology, China) supplemented with protease inhibitor cocktail (Roche, Switzerland). Antibody or IgG was added to the lysate and incubated with rotation overnight at 4 °C, then Protein A/G Magnetic Beads (HY‐K0202, MCE) was added to the lysate and incubated with rotation for 2 h at 4 °C, followed by three washes with PBST. Immunoprecipitation complexes were eluted with loading buffer and detected by Western blotting. The following antibodies were used: NEU4 (1:100, 12995‐1‐AP, Proteintech, China); YAP (1:100, sc‐376830, Santa Cruz Biotechnology, USA); HA‐Tag (1:100, AE105, Abclonal, China); IgG (A7017, A7028, Beyotime Biotechnology, China); IgG light chain (HRP) (1:1000, A25022, A25012, Abbkine, China).

To verify the binding protein of NEU4 or YAP, immunoprecipitation experiments were performed, followed by Western blotting. The cells were transfected with NEU4 or YAP plasmids for 24 h and then further treated with TGF‐*β*, with or without HMF, for 24 h.

### IP‐MS

To identify the binding protein of NEU4, immunoprecipitation experiments were performed, followed by in situ Western blotting and LC‐MS/MS (SpecAlly, CN). The cells were treated with TGF‐*β* for 24 h, and then were subjected to immunoprecipitation using NEU4 antibody or anti‐IgG antibody conjugated to Protein A/G Magnetic Beads. Subsequently, the beads were washed with 1% SDS in PBS, 0.1% SDS in PBS, and 6 m urea in PBS, respectively. Beads were enriched with protein and then separated using SDS‐PAGE, followed by detection using western blotting. For the identification of target proteins using LC‐MS/MS (Thermo Fisher Scientific, USA), the specific molecular weight bands were excised and washed, and the samples were reduced and alkylated using dithiothreitol and iodoacetamide, respectively. Subsequently, proteins were digested into peptides using trypsin. Finally, the peptides were analyzed using LC‐MS/MS. The MS data were retrieved using MaxQuant software (V1.6.6) with a MaxLFQ database retrieval algorithm and searched against the Uniport Human database. Proteins in modified form and common contaminating proteins were excluded, and the remaining identification and quantitative information were used for subsequent analysis. The IP‐NEU4 group was compared with the IP‐IgG group, and the quantitative difference ratio of each protein was calculated.

### Immunofluorescence Assay

For immunofluorescent analysis, HK‐2 cells or PTECs in a 96‐well plate were treated with or without TGF‐*β* and HMF (10, 20 µm), and then fixed with 4% paraformaldehyde (PFA) for 30 min, incubated with mouse primary antibody or rabbit primary antibody, followed by adding Alexa Fluor 594–labeled (red) anti mouse, Alexa Fluor 594–labeled (red) anti rabbit, Alexa Fluor 488–labeled (green) anti‐rabbit, Alexa Fluor 488–labeled (green) anti‐mouse antibodies, the cells were imaged by high content screening (Opera phenix, PerkinElmer, USA).

For NEU4 immunofluorescent analysis, HK‐2 cells in a glass plate were treated with TGF‐*β* for 24 h. The cells were imaged by STEDYCON (Abberior Instruments, Goettingen, Germany).

For the NEU4‐YAP interaction assay, the HK‐2 cells were treated with or without TGF‐*β* and HMF for 24 h, and then fixed with 4% paraformaldehyde (PFA) for 30 min, incubated with mouse primary antibody and rabbit primary antibody, followed by adding Alexa Fluor 594–labeled (red) anti‐mouse and Alexa Fluor 488–labeled (green) anti‐rabbit secondary antibodies. After staining with DAPI, the cells were imaged by high content screening (Opera phenix, PerkinElmer, USA).

For the kidney tissues immunofluorescence staining, sections were permeabilized with 0.1% Triton X‐100 and blocked with 5% BSA in PBS for 20 min at room temperature. Then, sections were incubated at 4 °C overnight with the primary antibodies. Fluorescently labeled secondary antibodies were used. Slides were counterstained with DAPI. Samples were analyzed, and pictures were taken using FV3000 confocal scanning microscope (Olympus Corporation, Japan).

The following antibodies were used: YAP (1:100, A21216, abclonal, China), Cytokeratin 18 (1:100, A19778, abclonal, China); E‐cadherin (1:100, 20874‐1‐AP, Proteintech, China), NEU4 (1:100, 12995‐1‐AP, Proteintech, China); N‐cadherin (1:100, sc‐59987), YAP (1:100, sc‐376830), Fibronectin (1:100, sc‐8422) and Vimentin (1:100, sc‐6260) (Santa Cruz Biotechnology, USA); *α*‐SMA (1:100, 19245S) (Cell Signaling Technology, USA); Anti‐alpha 1 Sodium Potassium ATPase (1:100, ab7671, Abcam, Britain). Alexa Fluor 594–labeled (red) anti mouse, Alexa Fluor 594–labeled (red) anti rabbit, Alexa Fluor 488–labeled (green) anti‐rabbit, Alexa Fluor 488–labeled (green) anti‐mouse antibodies (KGC6211‐0.1, KGC6210‐0.1, KGC6213‐0.1, KGC6214‐0.1, KeyGEN BioTEC, China), Ki67 (1:100, 14‐5698‐82, ThermoFisher, USA), *γ*H2AX (phospho S139) (C2036S, Beyotime Biotechnology, China).

### Plasmid Constructs and In Vitro Transfection

Mammalian expression plasmids for His‐ and HA‐tagged NEU4, His‐ and EGFP‐tagged YAP, His‐tagged YAP and NEU4 deletion mutants were constructed using the standard molecular cloning method from cDNA templates. Plasmids encoding NEU4 point mutation were constructed by site‐directed mutagenesis (MiaoLingBio, China). All constructs were confirmed by DNA sequencing.

Cells were transfected with small interfering (siRNA) or plasmid using Lipofectamine 2000 (ThermoFisher, USA), and the gene expression level was measured 24 h after transfection. The siRNA oligo sequences were as follows: negative control (NC) siRNA: 5ʹ‐UUCUCCGAACGUGUCACGUTT‐3ʹ, 3ʹ‐ACGUGACACGUUCGGAGAATT‐5ʹ; *NEU4* siRNA: 5ʹ‐ CCGUCUUCCUCUUCUUCAUTT‐3ʹ, 3ʹ‐AUGAAGAAGAGGAAGACGGTT‐5ʹ; *Neu4* siRNA: 5ʹ‐CAGAGGUCUUCUUGAACGUTT‐3ʹ, 3ʹ‐ ACGUUCAAGAAGACCUCUGTT‐5ʹ.

### RNAs Extraction, cDNA Synthesis, and Quantitative Real‐Time Reverse Transcription PCR (RT‐qPCR)

Total RNAs were extracted from cells and kidney tissue using TRIzol (Vazyme, China) and cDNA synthesis was carried out using high capacity cDNA reverse transcription kit (Vazyme, China) according to the manufacturer's instructions. Gene expressions were measured by the Roche LightCycler 96 System (Roche, Switzerland) using SYBR‐green as previously described.^[^ ^55^ ^]^ The mRNA expressions of respective genes were normalized to the level of *Gadph* or *β‐Actin* (*GADPH* or *β‐ACTIN*) mRNA and quantified by the 2^−ΔΔ^ *^C^* ^t^ method. Primer sequences were described in Table S3 (Supporting Information).

### In Vitro Kinase Assay

Neuraminidase enzyme activity was measured by a fluorometric assay with substrate 2′‐(4‐methylumbelliferyl)‐*α*‐d‐N‐acetylneuraminic acid (4‐MU‐NANA) (Abcam, ab138888, Britain). 293T cells were lysed with IP lysate after transfected with NEU4 plasmid 24 h. After centrifugation at 12000 × g at 4 °C for 15 min, the supernatants were incubated with the compound for 30 min respectively, then subjected to a neuraminidase assay at 37 °C for 1 h. Reaction products were measured at 320 nm for excitation and 450 nm for emission with microplate reader (GE Healthcare, USA).

### Bimolecular Fluorescence Complementation (BiFC)

293T cells were plated on glass bottom dish. Transfections were carried out using the lip2000 reagent, with *pBiFC‐NEU4(human)(254‐388aa)‐CC155* and *pBiFC‐YAP1(human)(231‐263aa)‐CrN173* plasmid (MiaoLingBio, China). For the control group, cells were transfected with *pBiFC‐NEU4(human)(254‐388aa)‐CC155* or *pBiFC‐YAP1(human)(231‐263aa)‐CrN173* plasmids. Fluorescence signal amplification was observed using the high content screening (Opera phenix, PerkinElmer, USA).

### Molecular Docking

The structures of NEU4 (UniProt Entry: Q8WWR8) was created by homology modeling at the RoseTTAFold website (https://github.com/RosettaCommons/RoseTT‐AFold). YAP (UniProt Entry: P46937) was obtained from Hermite (https://www.dp.tech/product/hermite). The 3D structures of the NEU4 and the YAP were uploaded for ZDOCK calculation. The resulting protein poses were ranked by ZDOCK scoring, and the protein pose was further processed using PyMOL Molecular Graphics System v2.5.2 (DeLano Scientific LLC, San Carlos, CA, USA).

The X‐ray crystal structure of NEU4 was used for the docking studies. Auto‐dock 4.2 (The Scripps Research Institute, California, USA) and PyRx 0.5 programs (The Scripps Research Institute, La Jolla, CA, USA) were employed for virtual screening, and the docked models were analyzed using PyMOL 2.5.2.

### Luciferase Reporter Assay

293T Cells were plated in 96‐well plates then transfected with *YAP*, *Luc‐CTGF*, *Luc‐CYR61*, *Luc‐NEGR1*, *Luc‐ANKRD1* and si*NEU4* or *NEU4* plasmid using Lipofectamine 2000 for 48 h. Cells were cleaved by cell lysis using 100 µL of Passive Lysis Buffer (PLB) per well for 96‐well plates. The luciferase activity was measured using luciferase assay kit (E1500, Promega, USA). The luciferase emission was measured with microplate reader (GE Healthcare, USA).

### Cell Viability Assay

After seeded for 24 h, the HK‐2 cells were administrated with HMF at the concentrations of 5, 10, 20 and 40 µm, respectively, for 24 h. 3‐(4,5‐Dimethylthiazol‐2‐yl)‐2,5‐diphenyltetrazoliumbromide (MTT) was added for 4 h, and detected the absorbance at 490 nm.

### Cellular Thermal Shift Assay

HK‐2 cells were treated with HMF (20 µm) or DMSO for 24 h, then aliquoted, and heated at different temperatures (37° and 60 °C) for 3 min. After cooling to room temperature, cells were lysed by freeze thawing in liquid nitrogen. Soluble proteins were collected by centrifugation at 12 000 g for 20 min at 4 °C and then detected by Western blotting.

HK‐2 cells were treated with HMF (20 µm) 24 h after transfection with EGFP and NEU4 or NEU4 (N302R, D307R) mutants.

### Flow Cytometric Analysis

The apoptotic cells were analyzed using flow cytometry. HK‐2 cells after treatment were digested with trypsin, washed twice with cold PBS, and centrifuged at 1000 r min^−1^ for 3 min at room temperature. Then, the cells were resuspended in 500 µL binding buffer containing 5 µL FITC‐annexin V and 5 µL propidium iodide (PI) (Keygen, Nanjing, China). The cells were subjected to gentle vortexing and incubated for 15 min at 25 °C in the dark. The degree of cell apoptosis was assessed by flow cytometry (BD, Franklin Lakes, NJ, USA).

### Surface Plasmon Resonance (SPR) Assay

Recombinant human NEU4 proteins were immobilized onto a CM5 chip via an EDC/NHS‐mediated crosslinking reaction, and the SPR analysis was performed on a Biacore TM T200 instrument (GE Healthcare, USA) according to manufacturer's instruction. The affinity fitting was carried out with Biacore T200 evaluation software by global fitting using a steady‐state affinity model to obtain the affinity constant *K_D_*.

### HMF Molecular Capture Experiments

To identify and quantify HMF target proteins, surface plasmon resonance (SPR) and high‐performance liquid chromatography (HPLC) mass spectrometry (MS) experiments were performed. HMF was printed on a chip surface by auto‐spotting three times using a BioDot‐1520 array printer (CA, USA).

For cell lysis dosage calibration, HK‐2 cells were lysed and calibrated the protein concentration using a bicinchoninic acid (BCA) protein assay kit (Thermo Scientific, USA).

In the SPR assay, HMF was immobilized on the surface of the chip, with HK‐2 cell lysate used as the liquid phase. Subsequently, the chip was collected and subjected to in situ enzymatic hydrolysis with trypsin, and the enriched protein on the chip surface was subsequently identified through nanoElute UHPLC system (Bruker Daltonics, USA). The peptides were subjected to Capillary source followed by the timsTOF Pro (Bruker Daltonics, USA) mass spectrometry.

The resulting MS/MS data were processed using MaxQuant search engine (version1.6.15.0). Tandem mass spectra were searched against human SwissProt database (Home‐sapiens‐9606‐SP‐20201214.fasta, 20395 entries) concatenated with reverse decoy database.

### Statistical Analysis

All data were expressed as mean ± standard error of the mean (SEM). Comparisons between two groups were analyzed by using a two‐tailed Student's *t* test, and those among three or more groups by using one‐way analysis of variance (ANOVA) followed by Dunnett's post hoc tests. Differences were considered significant at *p* < 0.05. The data from Western blot, RT‐qPCR, as well as the quantification of IHC and IF, were normalized. Statistical significance analyses were performed using GraphPad Prism version 8.0 (GraphPad Software, San Diego, CA, USA).

## Conflict of Interest

The authors declare no conflict of interest.

## Author Contributions

E‐H. L., Q.‐Q. C., L. Z., and P.‐T. X. designed the study protocol and supervised all parts of the project. P.‐T. X., J.‐H. H., Y.‐J. K., C.‐X.D., X.‐L.R., L.‐L. J. and Z.‐S. X. conducted animal experiments and the molecular biology experiments. P.‐T. X. and Q.‐Q. C. drafted the first versions. E‐H. L. and Q.‐Q. C. contributed to text revision. All authors read and approved the submitted version. P.‐T. X. and J.‐H. H. contributed equally to this work.

## Supporting information

Supporting Information

## Data Availability

All data needed to evaluate the conclusions in the paper are present in the paper and/or the Supplementary Materials. The mass spectrometry proteomics data were submitted to the ProteomeXchange Consortium, via the PRIDE partner repository (PXD051726).
