# Supplementary material for: Targeting Neuraminidase 4 Attenuates Kidney Fibrosis in Mice
Source: Adv Sci (Weinh). 2024 Aug 13;11(39):2406936. doi: 10.1002/advs.202406936 (PMC11497051; doi:10.1002/advs.202406936)
Supplement: Supplementary file 1 — Supporting Information [file ADVS-11-2406936-s001.pdf]

ADVANCED SCIENCE
Open Access

Supporting Information

for *Adv. Sci.*, DOI 10.1002/advs.202406936

Targeting Neuraminidase 4 Attenuates Kidney Fibrosis in Mice

Ping-Ting Xiao, Jin-Hua Hao, Yu-Jia Kuang, Cai-Xia Dai, Xiao-Ling Rong, Li-Long Jiang, Zhi-Shen Xie, Lei Zhang, Qian-Qian Chen* and E-Hu Liu**

Supporting Information

Targeting Neuraminidase 4 Attenuates Kidney Fibrosis in Mice

Ping-Ting Xiao, Jin-Hua Hao, Yu-Jia Kuang, Cai-Xia Dai, Xiao-Ling Rong, Li-Long Jiang, Zhi-Shen Xie, Lei Zhang *, *Qian-Qian Chen* *, *E-Hu Liu* *

Table S1 Clinical information of the subjects.

**Characteristics**	**Renal Fibrosis (*n* = 5)**	**Non-Renal Fibrosis (*n* = 4)**	***p* value**
Age (years)	41.6±12.7	23.25±10.3	0.05272
Male (n, %)	3 (20%)	1 (25%)	0.35582
SCr (µmol/L)	212.96±60.03	54.5±12.71	0.001368
BUN (mmol/L)	9.38±3.92	3.65±0.98	0.025749
eGFR (mL/(min*1.73m^2))	30.79±9.26	129.34±17.21	3.71E-05
Comorbidities/Medical history			
Hypertension	3 (60%)	0	
Diabetes mellitus	0	0	
COPD or asthma	1 (25%)	0	
Coronary disease	0	0	
Morbid obesity	0	0	
Neurologic	1 (25%)	0	
Neoplasms (extra-renal)	0	0	

Unpaired two-tailed *t*-test. SCr: serum creatinine; BUN: Blood urea nitrogen. eGFR: Estimated glomerular filtration rate; COPD: chronic obstructive pulmonary disease.

Table S2 The inhibitory rate of 67 compounds on NEU4 enzyme activity.

ID	Natural compound	Inhibition ratio±SD	Soureces	Structure
1	3,5,6,7,8,3′,4′-Heptamethoxyflavone	44.6%±0.016	Biopurify Phytochemicals	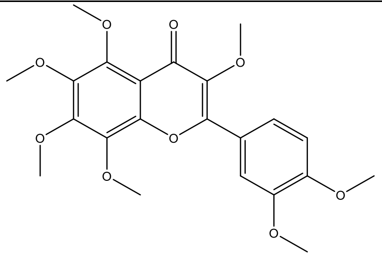
2	Didymin	10.9%±0.01	Biopurify Phytochemicals	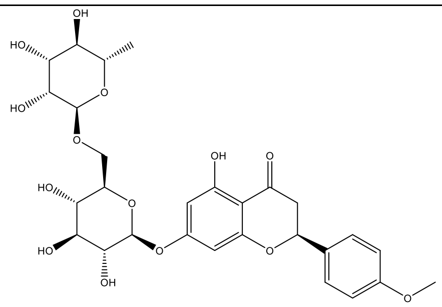
3	Narirutin	14.7%±0.023	Biopurify Phytochemicals	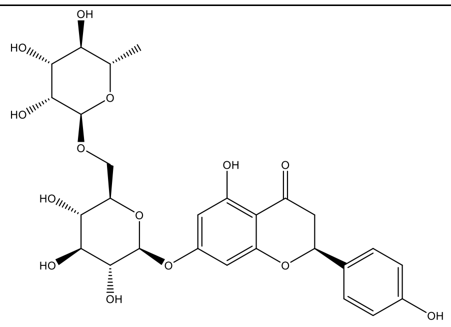
4	Naringin	16.9%+0.03	Must bio-technology	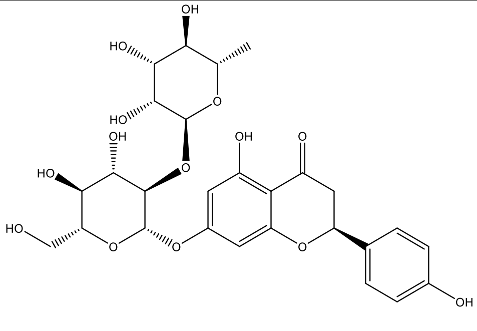

5	Synephrine	22.5%±0.074	Must bio-technology	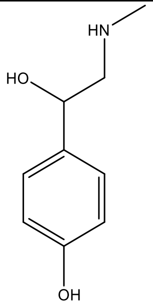
6	Neohesperidin	27.5%±0.009	Prepared in our laboratory	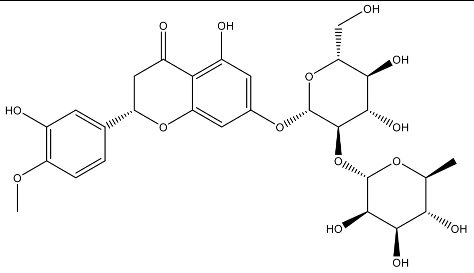
7	Hesperidin	25.7%±0.013	Must bio-technology	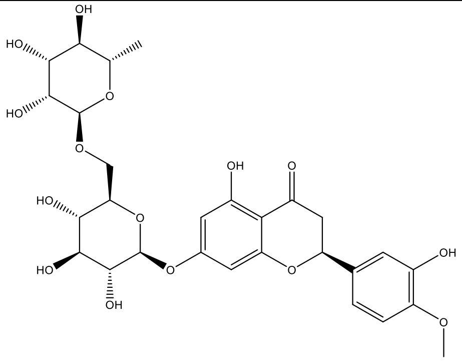
8	Berberine	15.6%±0.024	Must bio-technology	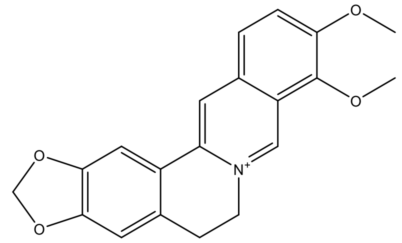

9	Macranthoidin B	6.4%±0.033	Must bio-technology	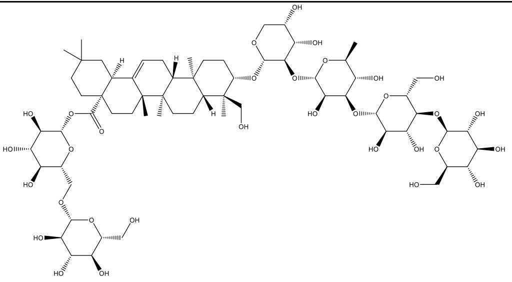
10	Ginsenoside Rg1	2.1%±0.014	Must bio-technology	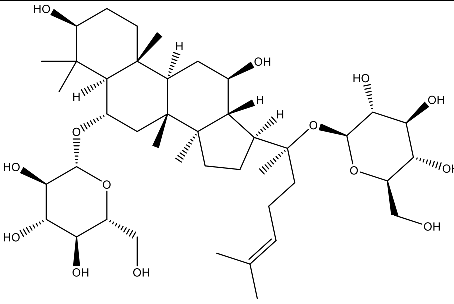
11	Cryptotanshinone	6.4%±0.032	Must bio-technology	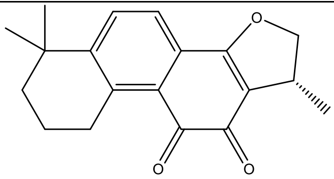
12	Catechin	16.2%±0.008	Must bio-technology	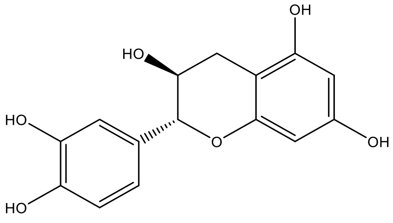

13	Jatrorrhizine	15.6%±0.04	Must bio-technology	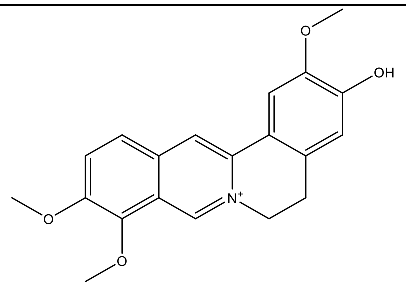
14	Ginsenoside Re	7.6%±0.012	Must bio-technology	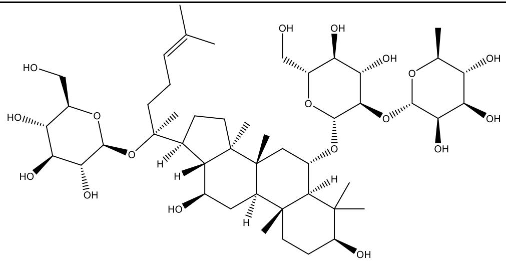
15	Ginsenoside Rd	11.3%±0.009	Must bio-technology	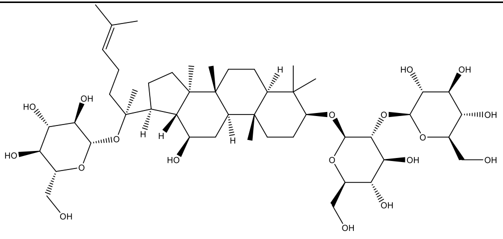
16	Astragaloside A	10.5%±0.02	Must bio-technology	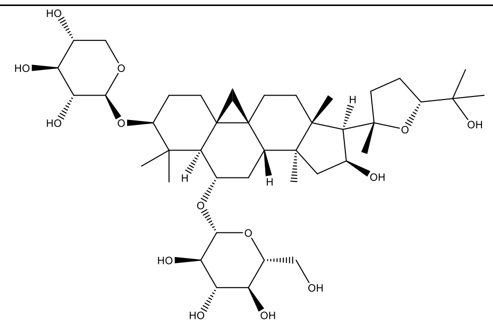

17	Sesamin	13.9%±0.018	Must bio-technology	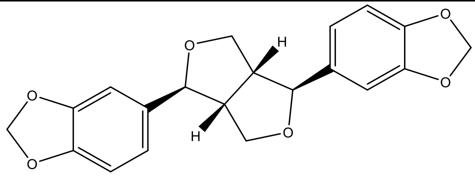
18	Limonin	10.6%±30.011	Must bio-technology	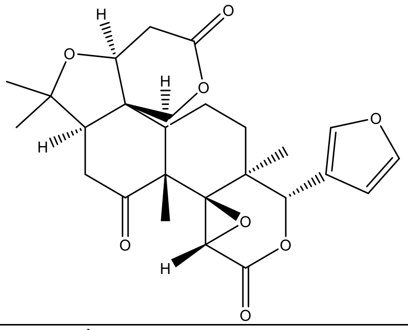
19	Alpinetin	14.1%±0.04	Must bio-technology	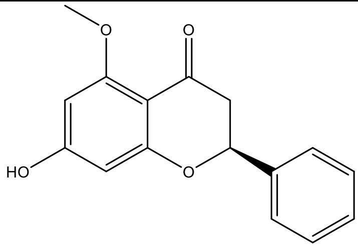
20	Magnoflorine	-182.4%±0.198	Must bio-technology	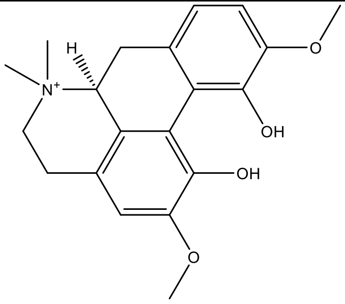

21	Schisandrin	7.4%±0.013	Must bio-technology	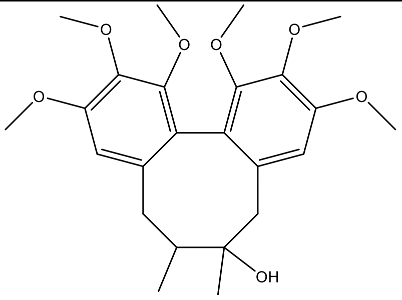
22	Tanshinone II A	3.3%±0.013	Must bio-technology	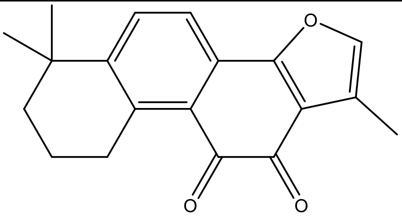
23	Dihydrotanshinone I	17.5%±0.02	Must bio-technology	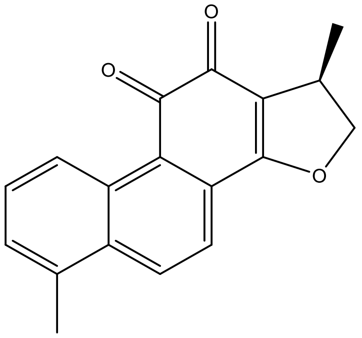
24	Isochlorogenic acid A	39.5%±0.103	Must bio-technology	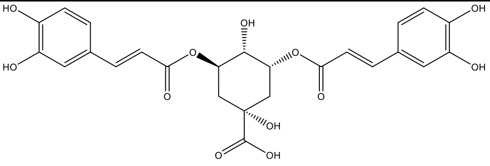

25	Daidzin	43.9%±0.036	Must bio-technology	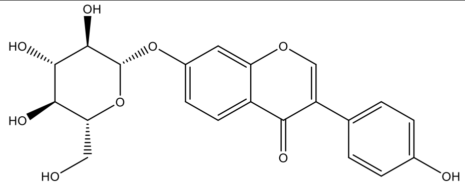
26	Tannic acid	-25.0%±0.03	Must bio-technology	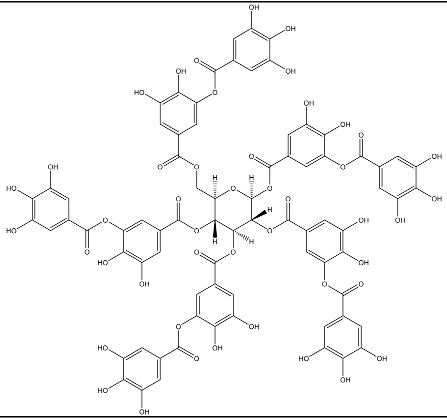
27	Glabridin	10.2%±0.038	Must bio-technology	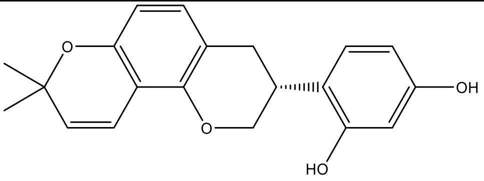
28	Genistin	19.3%±0.04	Must bio-technology	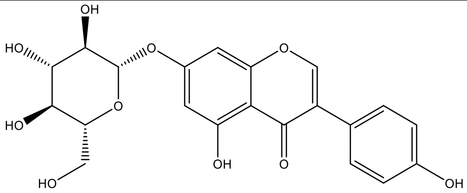

29	Daidzein	34.6%±0.053	Must bio-technology	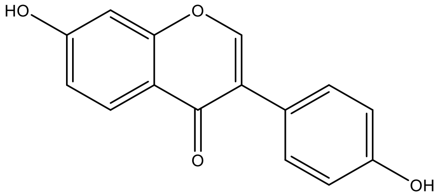
30	Stachydrine hydrochloride	4.9%±0.047	Must bio-technology	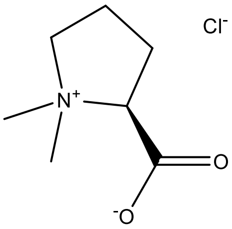
31	Dauricine	29.9%±0.088	Must bio-technology	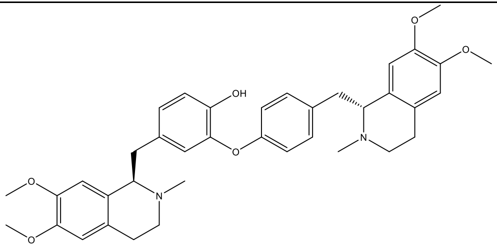
32	Shionone	1.7%±0.059	Must bio-technology	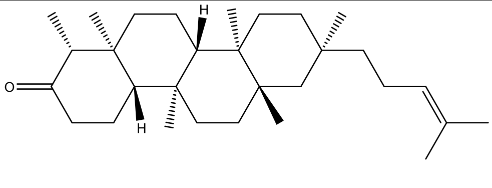

33	Betaine	2.8%±0.059	Must bio-technology	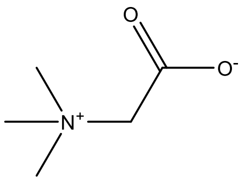
34	Cimifugin	-24.9%±0.026	Must bio-technology	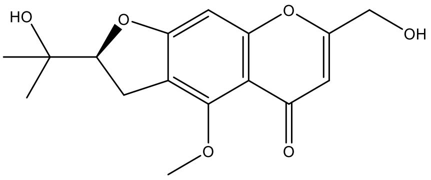
35	Bergapten	23.3%±0.024	Must bio-technology	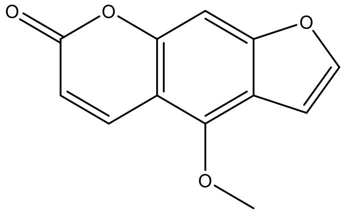
36	Costunolide	15.2%±0.014	Must bio-technology	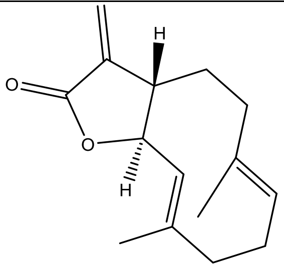

37	Icaritin	19.6%±0.022	Must bio-technology	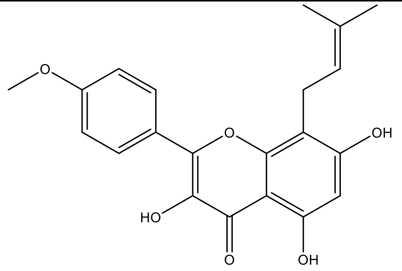
38	Veratramine	19.2%±0.011	Must bio-technology	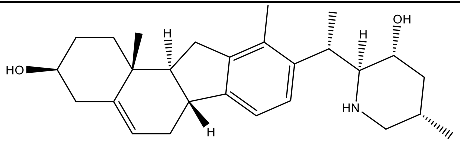
39	Scopoletin	-891.1%±0.242	Must bio-technology	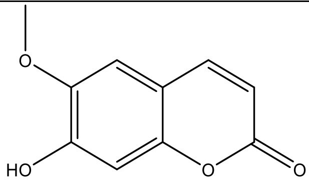
40	6-Gingerol	9.9%±0.032	Must bio-technology	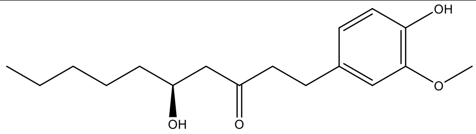

41	Dehydrocostus lactone	17.9%±0.019	Must bio-technology	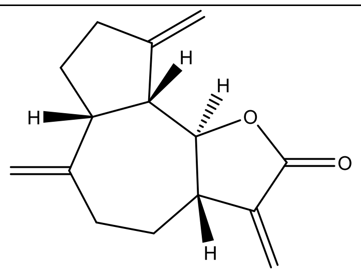
42	Loganic acid	-5.2%±0.012	Must bio-technology	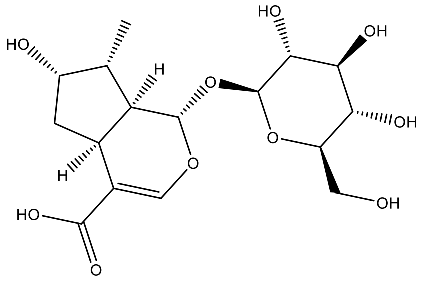
43	Aloe emodin	25.9%±0.208	Must bio-technology	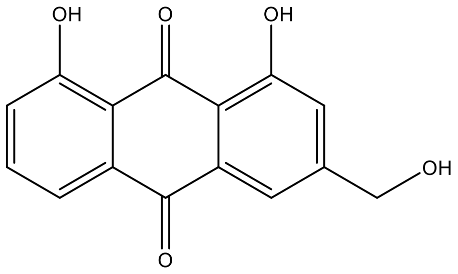
44	Psoralen	29.8%±0.012	Must bio-technology	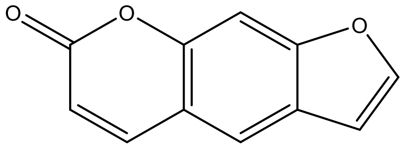

45	Gastrodin	3.6%±0.023	Must bio-technology	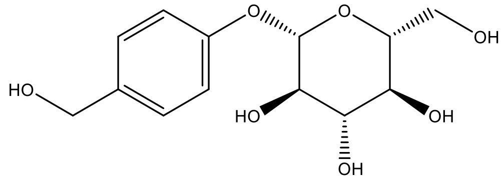
46	Macranthoside B	10.9%±0.033	Must bio-technology	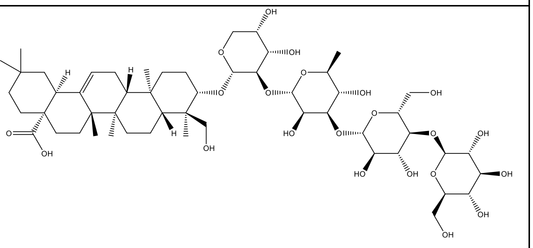
47	Baicalin	32.1%±0.033	Must bio-technology	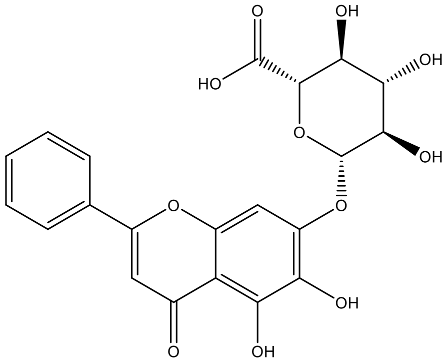
48	Saikosaponin	3.9%±0.045	Must bio-technology	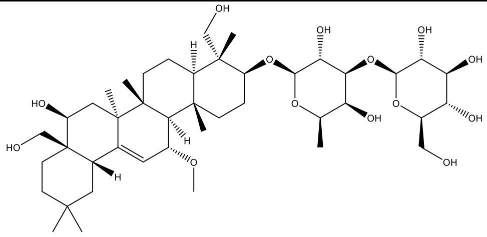

49	Curcumol	6.9%±0.004	Must bio-technology	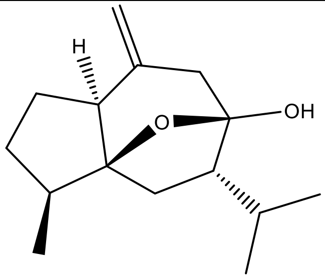
50	Alantolactone	12.4%±0.021	Must bio-technology	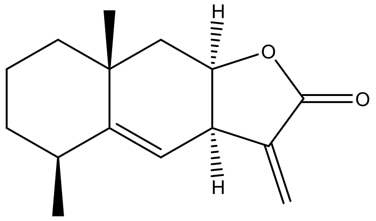
51	Parthenolide	15.1%±0.014	Must bio-technology	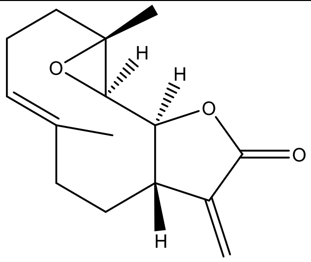
52	Diosmetin	17.5%±0.025	Must bio-technology	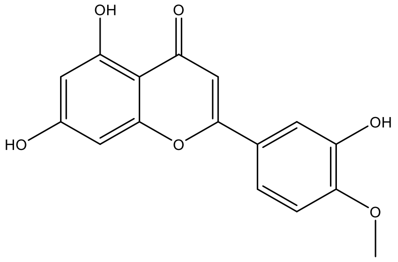

53	Carnosic acid	35.2%±0.004	Must bio-technology	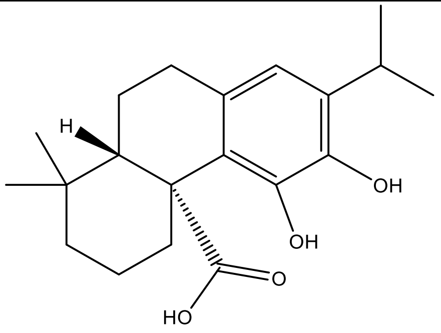
54	Salidroside	1.1%±0.011	Must bio-technology	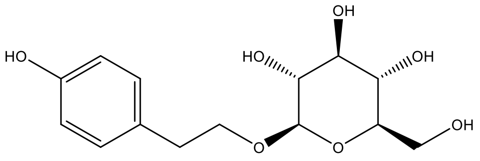
55	Esculin	-1054.69%±12.193	Must bio-technology	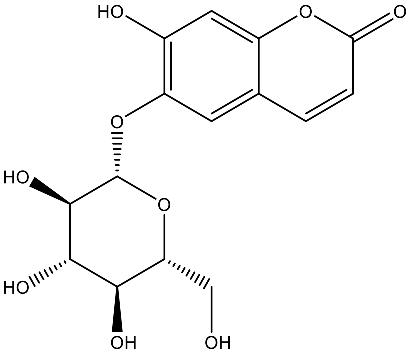
56	Evodiamine	-18.3%±0.087	Must bio-technology	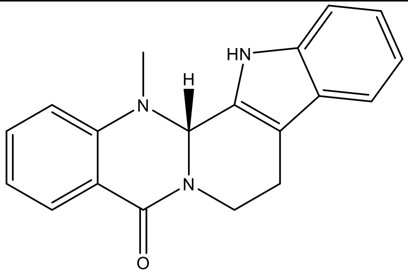

57	Deoxyschizandrin	1.4%±0.039	Must bio-technology	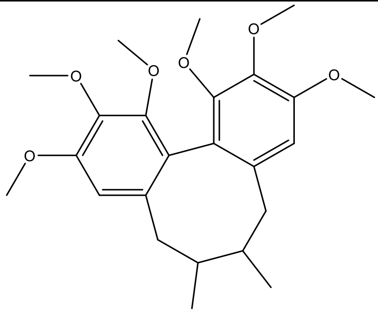
58	Sipeimine	1%±0.01	Must bio-technology	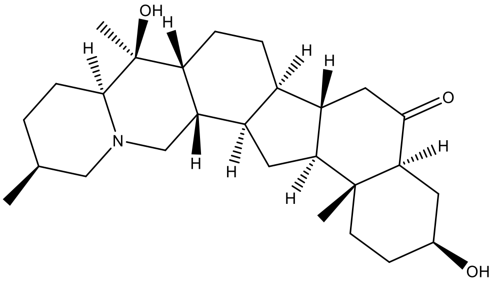
59	Notoginsenoside R1	-1.5%±0.008	Must bio-technology	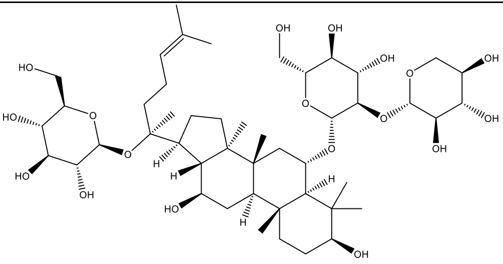
60	Glycyrrhizic acid	6.8%±0.019	Must bio-technology	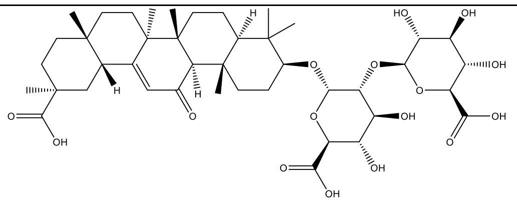

61	Isorhamnetin	22%±0.022	Must bio-technology	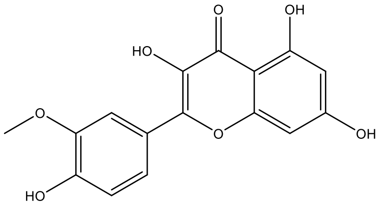
62	Apigenin	27.8%±0.010	Must bio-technology	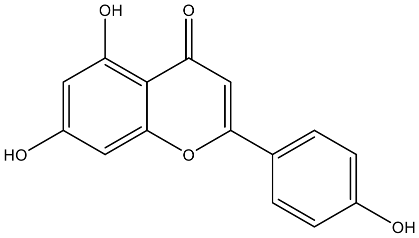
63	Nuciferine	14.1%±0.018	Must bio-technology	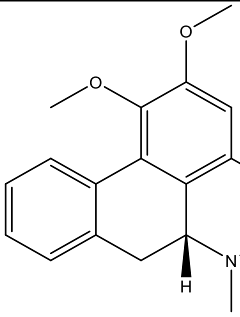
64	N-Nornuciferine	7.4%±0.042	Must bio-technology	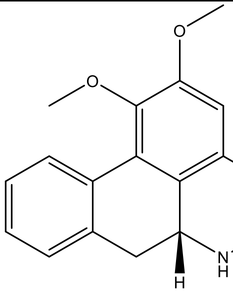

65	Cyasterone	6.3%±0.035	Must bio-technology	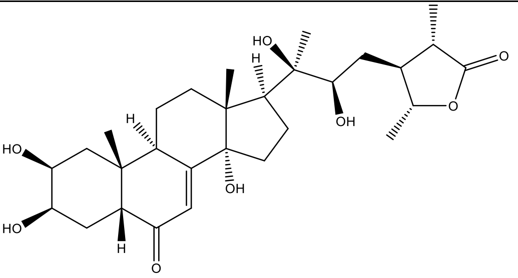
66	Stevioside	7.4%±0.014	Must bio-technology	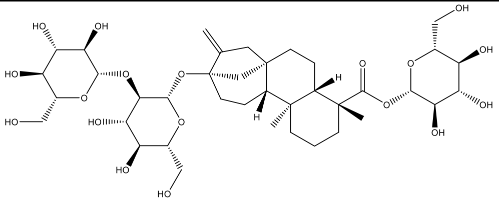
67	Epicatechin	28.1%±0.027	Must bio-technology	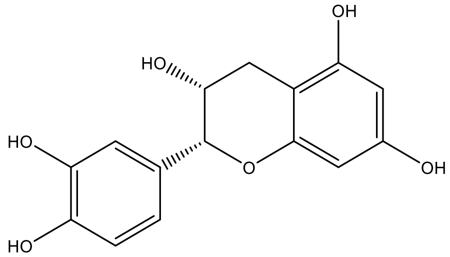
68	2-deoxy-2,3-didehydro-N-acetylneuraminic acid	53.3%±0.017	TCI	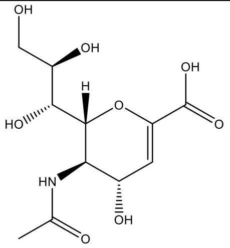

11

Table S3 Nucleotide sequences of gene-specific primers used for quantitative real-time reverse transcription PCR.

**Gene name**	**Primer**	**Sequence of primers (5′ to 3′)**
Human *α-SMA*	Forward	GCTGCCCAGAGACCCTGTT
	Reversed	TTTCATGGATGCCAGCAGACT
Human *VIMENTIN*	Forward	CAGAGAGAG GAAGCCGAAAG
	Reversed	ATGCTGTTCCTGAATCTGGG
Human *N-CADHERIN*	Forward	AAGAGAGACTGGGTCATCC
	Reversed	TGAGATGGGGTTGATAATG
Human *E-CADHERIN*	Forward	TAACAGGAACACAGGAGTCATCA
	Reversed	GTGGTGGGATTGAAGATCGG
Human *FN1*	Forward	CCACAGTGGAGTATGTGGTTAG
	Reversed	CAGTCCTTTAGGGCGATCAAT
Human *COL1A1*	Forward	TGTGCCACTCTGACTGGAAG
	Reversed	CGCCATACTCGAACTGGAATC
Human *COL3A1*	Forward	CGCCCTCCTAATGGTCAAGG
	Reversed	TTCTGAGGACCAGTAGGGCA
Human *COL4A1*	Forward	TGTTGACGGCTTACCTGGAGAC
	Reversed	GGTAGACCAACTCCAGGCTCTC
Human *CCL2*	Forward	CAGGTCCCTGTCATGCTTCT
	Reversed	GTCAGCACAGACCTCTCTCT
Human *PAI1*	Forward	GCACCACAGACGCGATCTT
	Reversed	ACCTCTGAAAAGTCCACTTGC
Human *TGF-β*	Forward	CTAATGGTGGAAACCCACAACG
	Reversed	TATCGCCAGGAATTGTTGCTG
Human *ARG1*	Forward	TCAGAGCATGAGCGCCAAGT
	Reversed	CCTCGTGGCTGTCCCTTTGA
Human *NEU4*	Forward	GGCCACGGGATGACAGTTG
	Reversed	CAGGCGGATACCCATGTGTAG
Human *SNAI1*	Forward	TCGGAAGCCTAACTACAGCG
	Reversed	CAGATGAGCATTGGCAGCGA
Human *KIM-1*	Forward	CCCACGTCACCTATCGGAAG

	Reversed	GTGCTCAACACGGCAACAAT
Human *TNFα*	Forward	CCTCTCTCTAATCAGCCCTCTG
	Reversed	GAGGACCTGGGAGTAGATGAG
Human *IL6*	Forward	ACTCACCTCTTCAGAACGAATTG
	Reversed	CCATCTTTGGAAGGTTCAGGTTG
Human *IL1β*	Forward	AGCTACGAATCTCCGACCAC
	Reversed	CGTTATCCCATGTGTCGAAGAA
Human *IL10*	Forward	ACCTGCCTAACATGCTTCGAG
	Reversed	GGCATCACCTCCTCCAGGTA
Human *P21*	Forward	TCTTGTACCCTTGTGCCTCG
	Reversed	ATCTGTCATGCTGGTCTGCC
Human *P16*	Forward	TGAAGCTCCCAGAATGCCAG
	Reversed	GCTGCCCTGGTAGGTTTTCT
Human *P53*	Forward	ACTTCAGGGGTGCCACATTC
	Reversed	CGACCCTGTCCCTCACCTCC
Human *IL8*	Forward	ACTCCAAACCTTTCCACCCC
	Reversed	CCTCTGCACCCAGTTTTCCT
Human *KI67*	Forward	GGAAGCTGGACGCAGAAGAT
	Reversed	CAGCACCATTTGCCAGTTCC
Human *γH2AX*	Forward	AGAAGACGCGAATCATCCCC
	Reversed	TGGTCTTCTTGGGCAGCAG
Human *GAPDH*	Forward	GGAGCGAGATCCCTCCAAAAT
	Reversed	GGCTGTTGTCATACTTCTCATGG
Human *β-actin*	Forward	GATCATTGCTCCTCCTGAGC
	Reversed	ACTCCTGCTTGCTGATCCAC
Mouse *α-Sma*	Forward	GGACTTTGAAAATGAGATGG
	Reversed	TGATGCTGTTATAGGTGGTT
Mouse *Vimentin*	Forward	CAGAGAGAG GAAGCCGAAAG
	Reversed	ATGCTGTTCCTGAATCTGGG
Mouse *N-cadherin*	Forward	AAGAGAGACTGGGTCATCC
	Reversed	TGAGATGGGGTTGATAATG
Mouse *Fibronectin*	Forward	CCACAGTGGAGTATGTGGTTAG

	Reversed	CAGTCCTTTAGGGCGATCAAT
Mouse *Kim-1*	Forward	CTATGTTGGCATCTGCATCG
	Reversed	AAGGCAACCACGCTTAGAGA
Mouse *Neu4*	Forward	GAACAGCGACTTAGCCCTGATG
	Reversed	TAGACCTGTGCTCCTCCAGTAC
Mouse *Snai1*	Forward	CTCCAAACCCACTCGGATGT
	Reversed	AGCCAGACTCTTGGTGCTTG
Mouse *Snai2*	Forward	GCCTCCAAGAAGCCCAACTA
	Reversed	GCCGACGATGTCCATACAGT
Mouse *E-cadherin*	Forward	TAACAGGAACACAGGAGTCATCA
	Reversed	GTGGTGGGATTGAAGATCGG
Mouse *Col1a1*	Forward	TTGGAGAGAGCATGACCG
	Reversed	TACGCTGTTCTTGCAGTG
Mouse *Col3a1*	Forward	GTCTGGTGGCTTTTCACCCT
	Reversed	AGTTCGGGGTGGCAGAATTT
Mouse *Col4a1*	Forward	AACAACGTCTGCAACTTCGC
	Reversed	CTTCACAAACCGCACACCTG
Mouse *Ccl2*	Forward	CAGGTCCCTGTCATGCTTCT
	Reversed	GTCAGCACAGACCTCTCTCT
Mouse *Tgf-β*	Forward	GACCGCAACAACGCCATCTA
	Reversed	GGCGTATCAGTGGGGGTCAG
Mouse *Mmp2*	Forward	ACCTGAACACTTTCTATGGCTG
	Reversed	CTTCCGCATGGTCTCGATG
Mouse *Mmp7*	Forward	TAGGCGGAGATGCTCACTTT
	Reversed	TTCTGAATGCCTGCAATGTC
Mouse *Mmp9*	Forward	CTGGACAGCCAGACACTAAAG
	Reversed	CTCGCGGCAAGTCTTCAGAG
Mouse *Mmp13*	Forward	CTGGTCTTCTGGCACACGCT
	Reversed	GCAGCGCTCAGTCTCTTCAC
Mouse *Fsp1*	Forward	TGAGCAACTTGGACAGCAACA
	Reversed	TTCCGGGGTTCCTTATCTGGG
Mouse *Timp1*	Forward	GCAACTCGGACCTGGTCATAA

	Reversed	CGGCCCGTGATGAGAAACT
Mouse *Tnfα*	Forward	TCTCATGCACCACCATCAAGGACT
	Reversed	ACCACTCTCCCTTTGCAGAATCA
Mouse *Il6*	Forward	ATCCAGTTGCCTTCTTGGGACTGA
	Reversed	TAAGCCTCCGACTTGTGAAGTGGT
Mouse *Il1β*	Forward	GCAACTGTTCCTGAACTCAACT
	Reversed	ATCTTTTGGGGTCCGTCAACT
Mouse *Yap*	Forward	CCAGACGACTTCCTCAACAGTG
	Reversed	GCATCTCCTTCCAGTGTGCCAA
Mouse *Negr1*	Forward	GCCTTCGAGTGGTACAAAGGA
	Reversed	CTGTACTTGGAGGGTTGAGGG
Mouse *Ankrd1*	Forward	ATAAACGGACGGCACTCCAC
	Reversed	CATCTGCGTTTCCTCCACGA
Mouse *Ctgf*	Forward	GCCTACCGACTGGAAGACAC
	Reversed	GTAACTCGGGTGGAGATGCC
Mouse *Cyr61*	Forward	ATGACCTCCTCGGACTCGAT
	Reversed	GGGTTGAAAAGAACTCGCGG
Mouse *P21*	Forward	TTGTCGCTGTCTTGCACTCT
	Reversed	TTTCGGCCCTGAGATGTTCC
Mouse *P16*	Forward	GAACTCTTTCGGTCGTACCCC
	Reversed	TAGTGGGGTCCTCGCAGTT
Mouse *P53*	Forward	TGGAGGAGTCACAGTCGGAT
	Reversed	CGTCCATGCAGTGAGGTGAT
Mouse *Il8*	Forward	TGGGTGAAGGCTACTGTTGG
	Reversed	AGCTTCATTGCCGGTGGAAA
Mouse *GAPDH*	Forward	TGTGTCCGTCGTGGATCTGA
	Reversed	CCTGCTTCACCACCTTCTTGAT
Mouse *β-actin*	Forward	GCTCTGGCTCCTAGCACC
	Reversed	CCACTATCCACACAGAGTACTTG

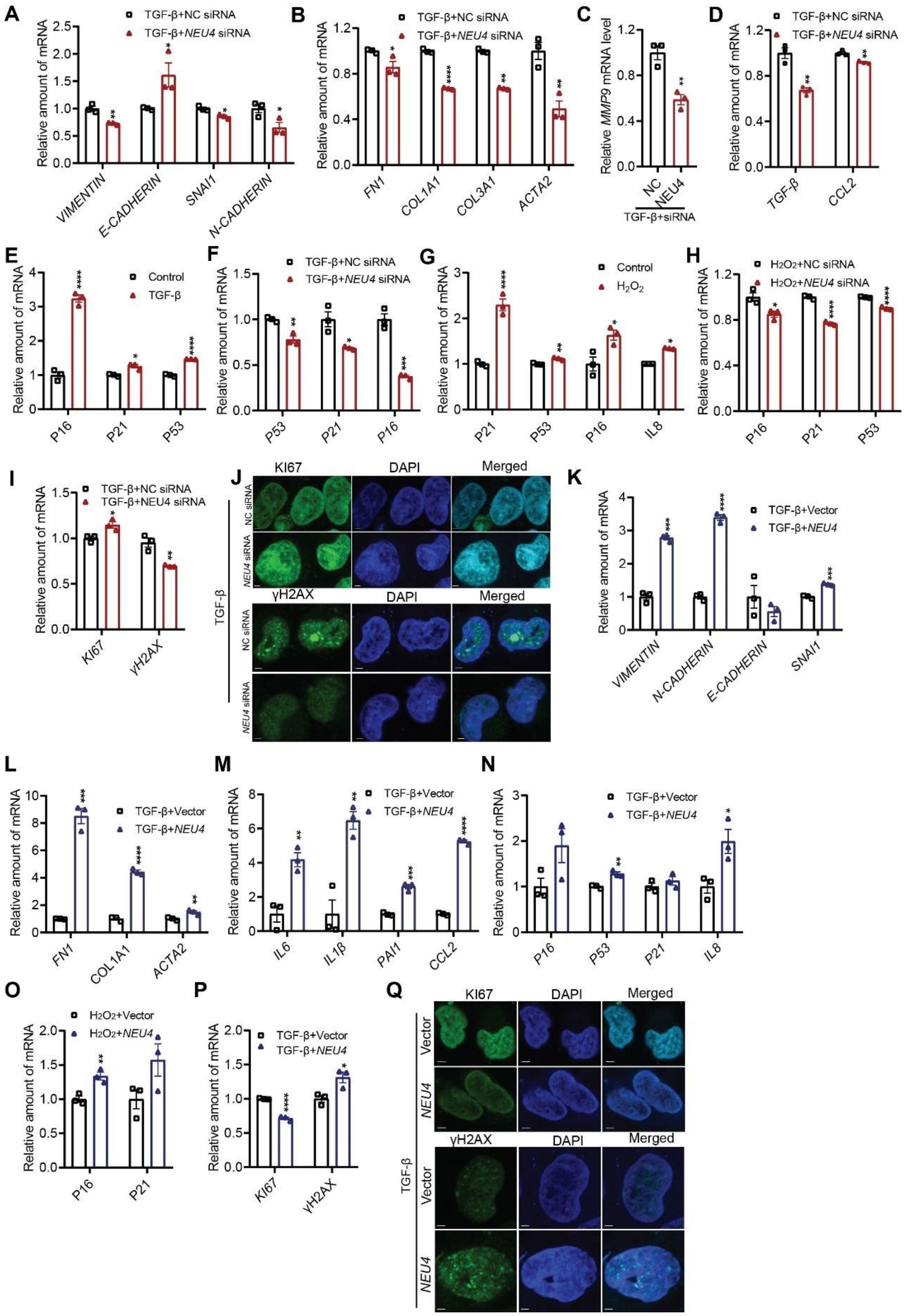

Figure S1. NEU4 promoted epithelial-mesenchymal transition (EMT) and cellular senescence in TGF-β-induced HK-2 cells. (**A-F, I**) Relative EMT associated gene (**A**), ECM associated gene (**B**), *MMP9* (**C**), chemokine associated (**D**), senescence-

associated gene (**E, F**) and DNA damage marker gene (I) mRNA level in HK-2 cells. *n* = 3 samples. (**G, H**) Relative senescence-associated gene mRNA level in HK-2 cells. *n* = 3 samples. (**J, Q**) Immunofluorescent staining represents KI67 or γH2AX in HK-2 cells. Scale bar, 10 μm, *n* = 3 samples. (**K-P**) Relative EMT associated gene (**K**), ECM associated gene (**L**), inflammation associated gene (**M**), senescence-associated gene (**N, O**) and DNA damage marker gene (**P**) mRNA level in HK-2 cell. *n* = 3 samples. (**A-D, F, I, J**) HK-2 cells treatment with TGF-β 24 h after transfection with *NEU4* siRNA. (**E, G**) HK-2 cells treatment with TGF-β 24 h or H_2O_2 6 h. (**K-Q**) HK-2 cells treatment with TGF-β 24 h after transfection with *NEU4*-overexpression plasmids. (**H**, **O**) HK-2 cells treatment with H_2O_2 6 h after transfection with *NEU4* siRNA (**H**) or *NEU4*-overexpression plasmids (**O**). Data are presented as mean ± SEM. Comparisons between two groups were analyzed by using a two-tailed Student's *t* test. *$p<0.05$, **$p<0.01$, ***$p<0.001$, ****$p<0.0001$ versus the NC siRNA or Vector group.

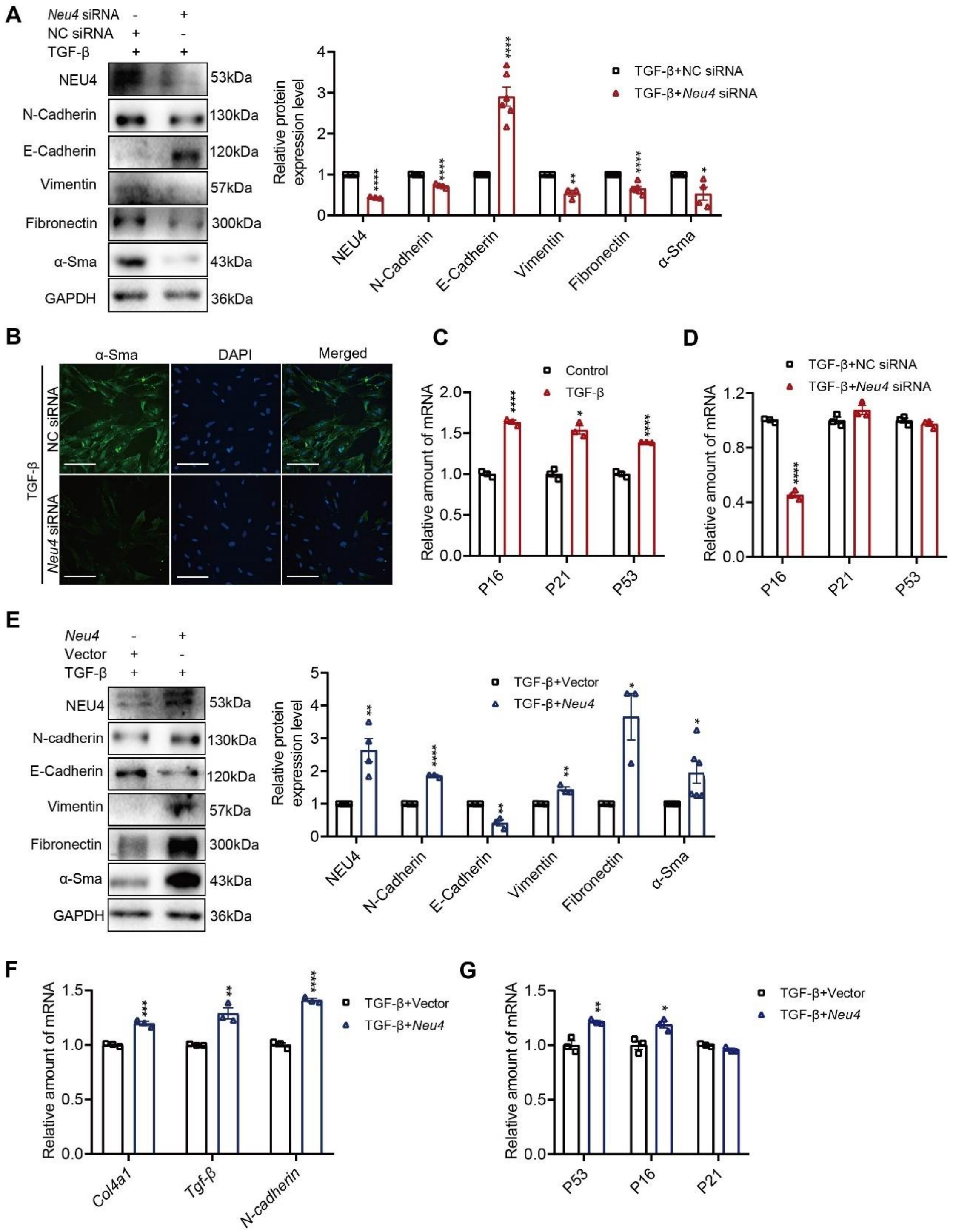

Figure S2. NEU4 promoted EMT and cell senescence in TGF-β-induced PTECs. (**A**) Western blot (left panel) and quantification (right panel) of the protein expression of NEU4, N-Cadherin, E-Cadherin, Vimentin, Fibronectin and α-Sma in PTECs. GAPDH served as loading control, n = 3-6 samples. (**B**) Immunofluorescent staining represents α-Sma in PTECs. Scale bar, 100 μm, n = 3 samples. (**C**, **D**) Relative senescence associated gene mRNA level in PTECs, n = 3 samples. (**E**) Western blot

(left panel) and quantification (right panel) of the protein expression of NEU4, N-Cadherin, E-Cadherin, Vimentin, Fibronectin and α-Sma in PTECs. GAPDH served as loading control, $n = 3$-6 samples. (**F, G**) Relative EMT associated gene, ECM associated gene (**F**) and senescence associated gene (**G**) mRNA level in PTECs, $n = 3$ samples. (**C**) PTECs treatment with TGF-β 24 h. (**A**-**B, D**) PTECs treatment with TGF-β 24 h after transfection with *Neu4* siRNA. (**E-G**) PTECs treatment with TGF-β 24 h after transfection with *Neu4*-overexpression plasmids. Error bars represent mean ± SEM. Comparisons between two groups were analyzed by using a two-tailed Student′s *t* test. *$p<0.05$, **$p<0.01$, ***$p<0.001$, ****$p<0.0001$ versus the NC siRNA or Vector group.

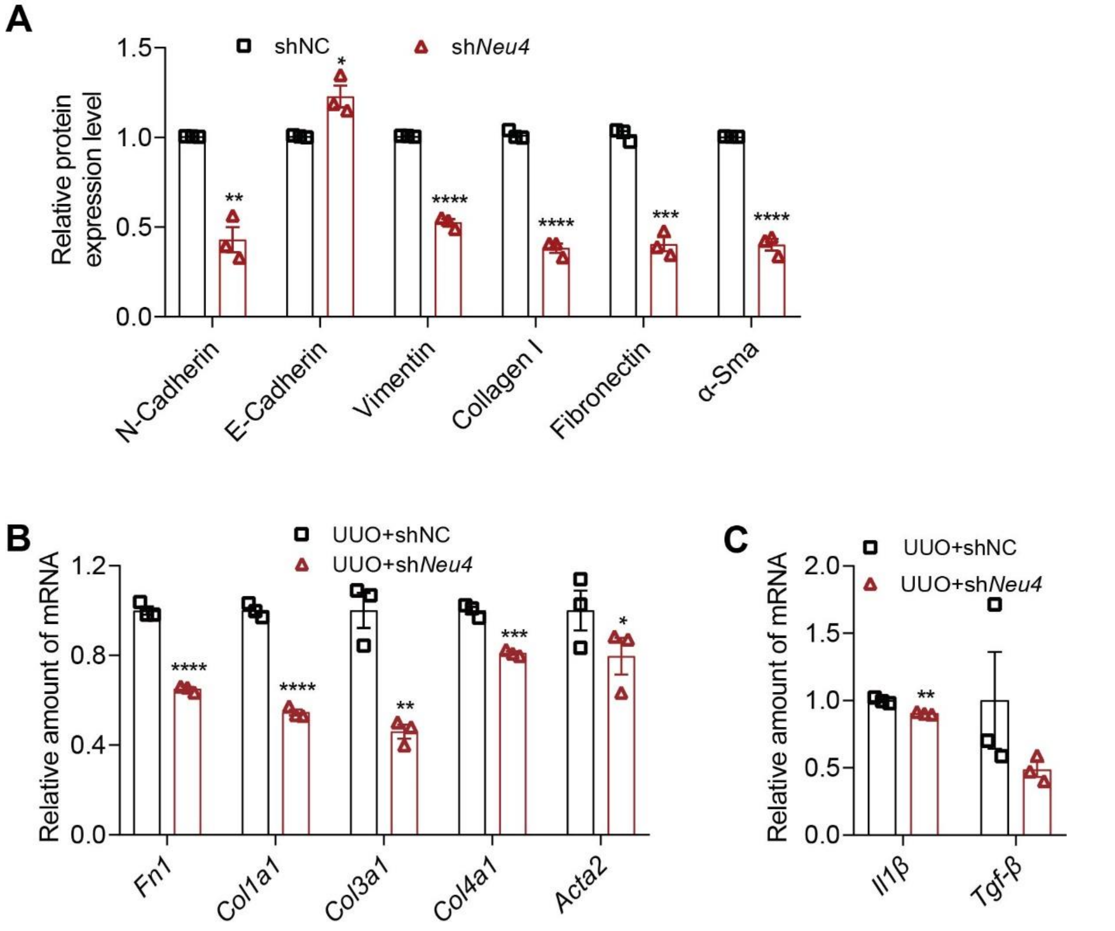

Figure S3. *Neu4* knockdown alleviated UUO-induced EMT, ECM and inflammation in mice. (**A**) Quantification of the expression of N-Cadherin, E-Cadherin, Vimentin, Collagen I, Fibronectin and α-Sma as shown in Figure 3E. GAPDH served as loading control. n = 3-5 mice. (**B**, **C**) Relative mRNA level of ECM associated genes (**B**) and inflammation associated genes (**C**) were determined by RT-qPCR, n = 3 mice. Error bars represent mean ± SEM. Comparisons between two groups were analyzed by using a two-tailed Student′s t test. *p<0.05, ****p<0.0001 versus the shNC.

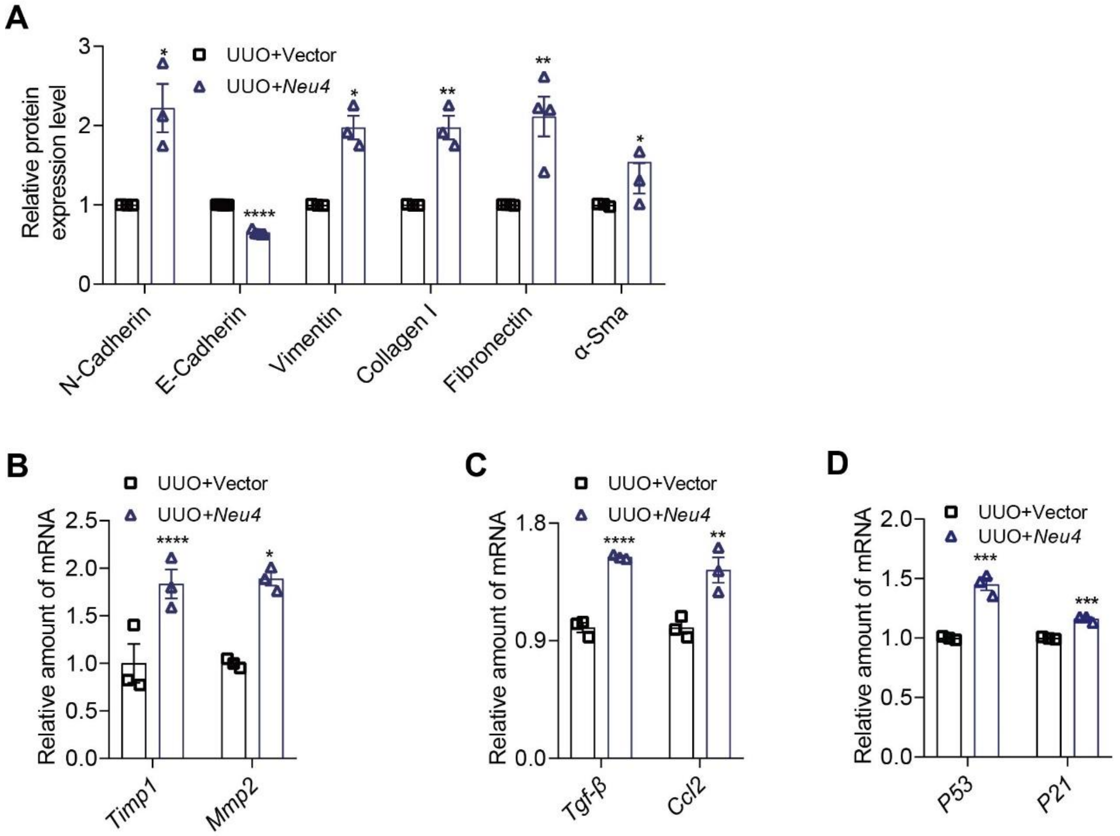

Figure S4. *Neu4* overexpression aggravated UUO-induced EMT and cellular senescence in mice. (**A**) Quantification of the protein expression of α-Sma, N-Cadherin, E-Cadherin, Vimentin, Fibronectin and Collagen I in kidneys as shown in Figure 4E. GAPDH served as loading control, n = 3-5 mice. (**B-D**) Relative extracellular matrix associated gene (**B**), chemokine associated (**C**) and senescence associated gene (**D**) mRNA level in left kidney. n = 3 mice. Error bars represent mean ± SEM. Comparisons between two groups were analyzed by using a two-tailed Student′s t test. *p<0.05, **p<0.01, ***p<0.001, ****p<0.0001 versus the Vector group.

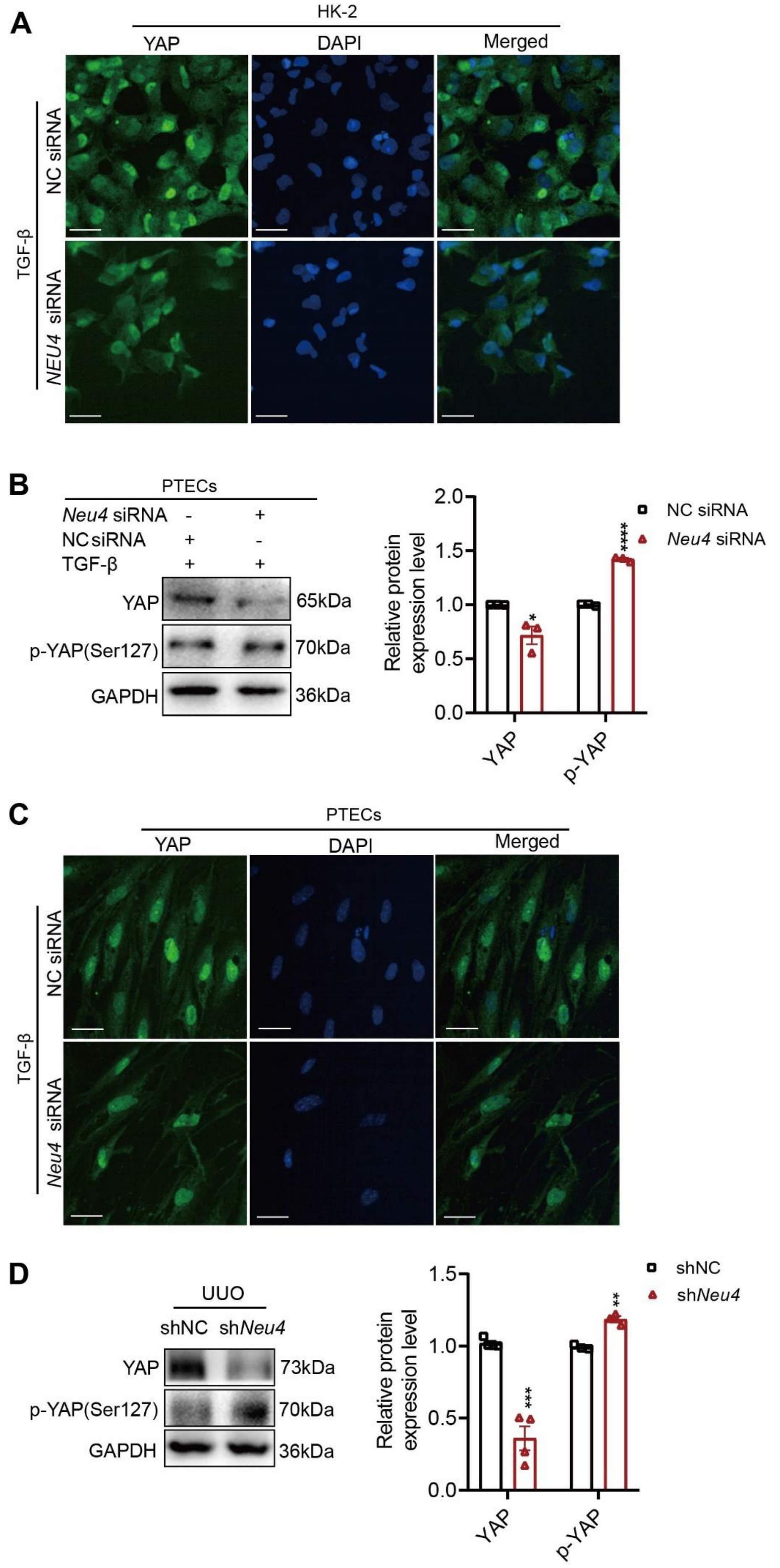
A
HK-2
YAP
DAPI
Merged
TGF-β
NC siRNA
NEU4 siRNA
B
PTECs
Neu4 siRNA - +
NC siRNA + -
TGF-β + +
YAP
65kDa
p-YAP(Ser127)
70kDa
GAPDH
36kDa
Relative protein expression level
NC siRNA
Neu4 siRNA
YAP
p-YAP
C
PTECs
YAP
DAPI
Merged
TGF-β
NC siRNA
Neu4 siRNA
D
UUO
shNC
shNeu4
YAP
73kDa
p-YAP(Ser127)
70kDa
GAPDH
36kDa
Relative protein expression level
shNC
shNeu4
YAP
p-YAP

Figure S5. NEU4 knockdown inhibits activation of Yes-associated protein (YAP). Related to Figure 6. (**A**) Immunofluorescent staining represents YAP in HK-2 cells treatment with TGF-β 24 h after transfection with *Neu4* siRNA. Scale bar, 100 μm, n = 3 samples. (**B**) Western blot (left panel) and quantification (right panel) of the protein expression of YAP and phosphorylation of YAP in PTECs. GAPDH served as loading control, n = 3 samples. (**C**) Immunofluorescent staining represents YAP in PTECs. Scale bar, 100 μm, n = 3 samples. (**D**) Western blot (left panel) and quantification (right panel) of the protein expression of YAP and phosphorylation of YAP in left kidneys from UUO mice with sh*Neu4*. GAPDH served as loading control, n = 3-4 mice. (**B** and **C**) PTECs treatment with TGF-β 24 h after transfection with *Neu4* siRNA. Error bars represent mean ± SEM. Comparisons between two groups were analyzed by using a two-tailed Student′s t test. *p<0.05, **p<0.01, ***p<0.001, ****p<0.0001 versus the NC siRNA or shNC group.

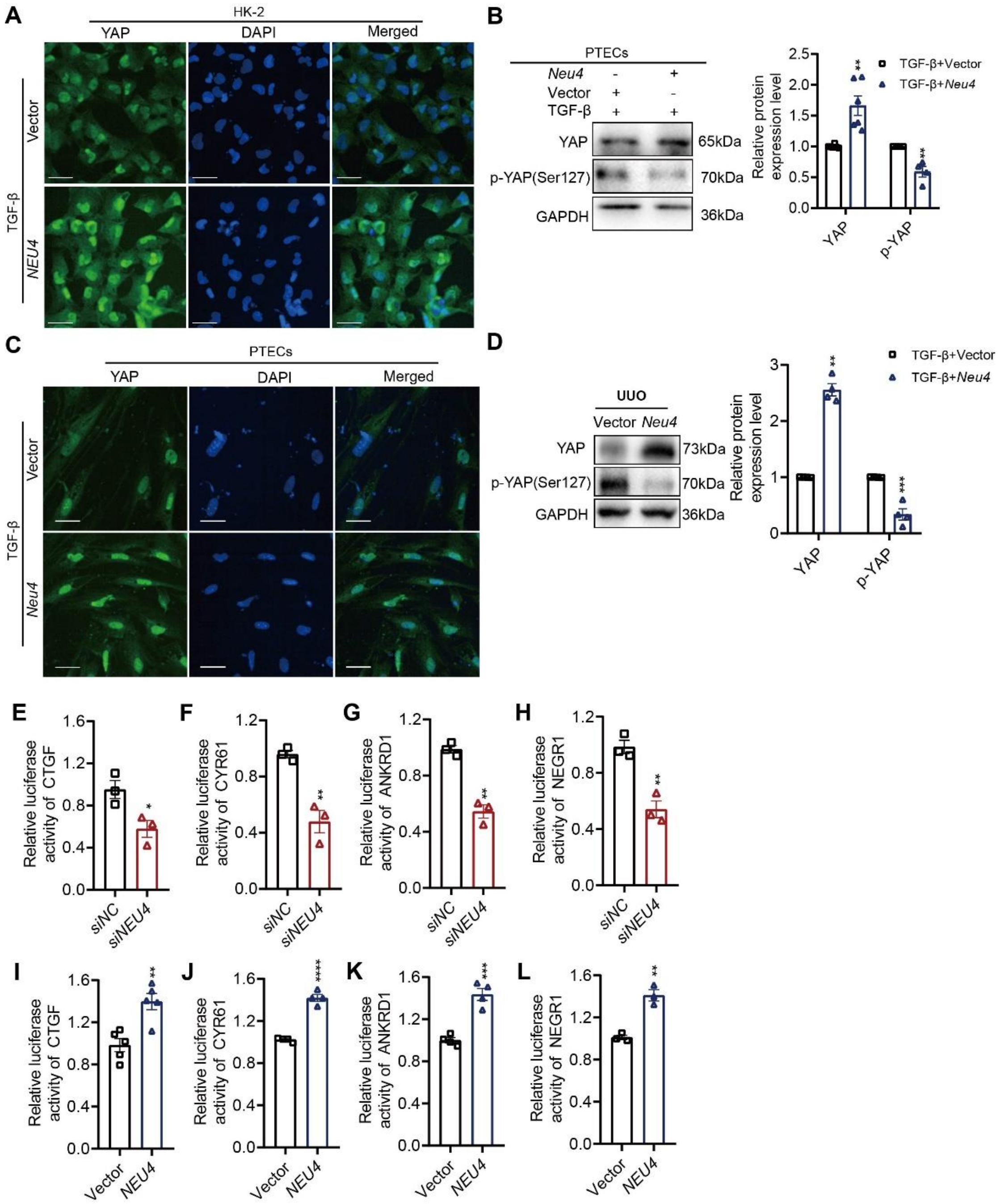

Figure S6. NEU4 overexpression promotes activation of YAP. (**A**) Immunofluorescent staining represents YAP in HK-2 cells treatment with TGF-β 24 h after transfection with *NEU4*-overexpression plasmids. Scale bar, 100 μm, n = 3 samples. (**B**) Western blot (left panel) and quantification (right panel) of YAP and phosphorylation of YAP in PTECs, GAPDH served as loading control, n = 4-6 samples. (**C**) Immunofluorescent staining represents YAP in PTECs. Scale bar, 100 μm, n = 3 samples. (**D**) Western blot (left panel) and quantification (right panel) of the protein

expression of YAP and phosphorylation of YAP in left kidneys from UUO mice with *Neu4*-overexpression plasmids. GAPDH served as loading control, $n = 4$ mice. (**B** and **D**) PTECs treatment with TGF-β 24 h after transfection with *Neu4*-overexpression plasmids. (**E-L**) 293T cells were co-transfected with *YAP* and *Luc-CTGF* or *Luc-CYR61* or *Luc-ANKRD1* or *Luc-NEGR1* and si*NEU4* (E-H) or *NEU4* plasmid (I-L) for 48 h. Luciferase activity was determined using the luciferase reporter system. $n = 3\text{-}5$ samples. Error bars represent mean ± SEM. Comparisons between two groups were analyzed by using a two-tailed Student's t test. *$p<0.05$, **$p<0.01$, ***$p<0.001$, ****$p<0.0001$ versus the Vector group.

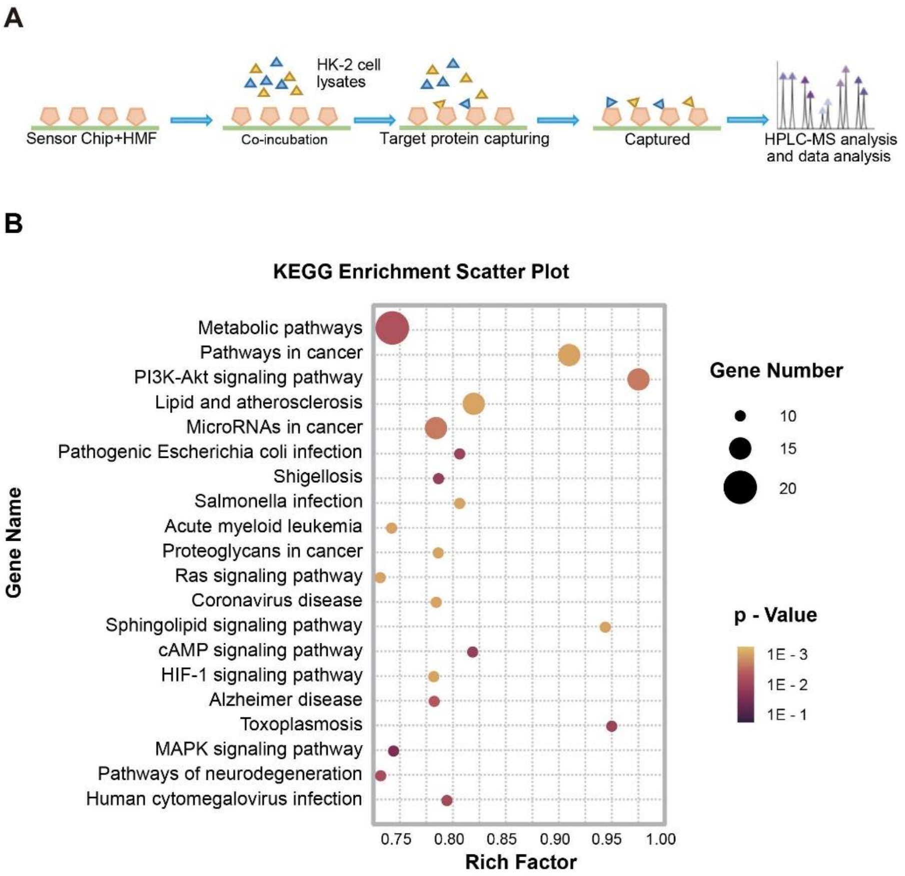

Figure S7. NEU4 served as a cellular target of anti-fibrosis small-molecule HMF. (**A**) Schematic representation of the HMF molecular capture protocol. (**B**) Genomes (KEGG) pathway analysis of the identified changed proteins in HK-2 cells 24 h after treatment with TGF-β.

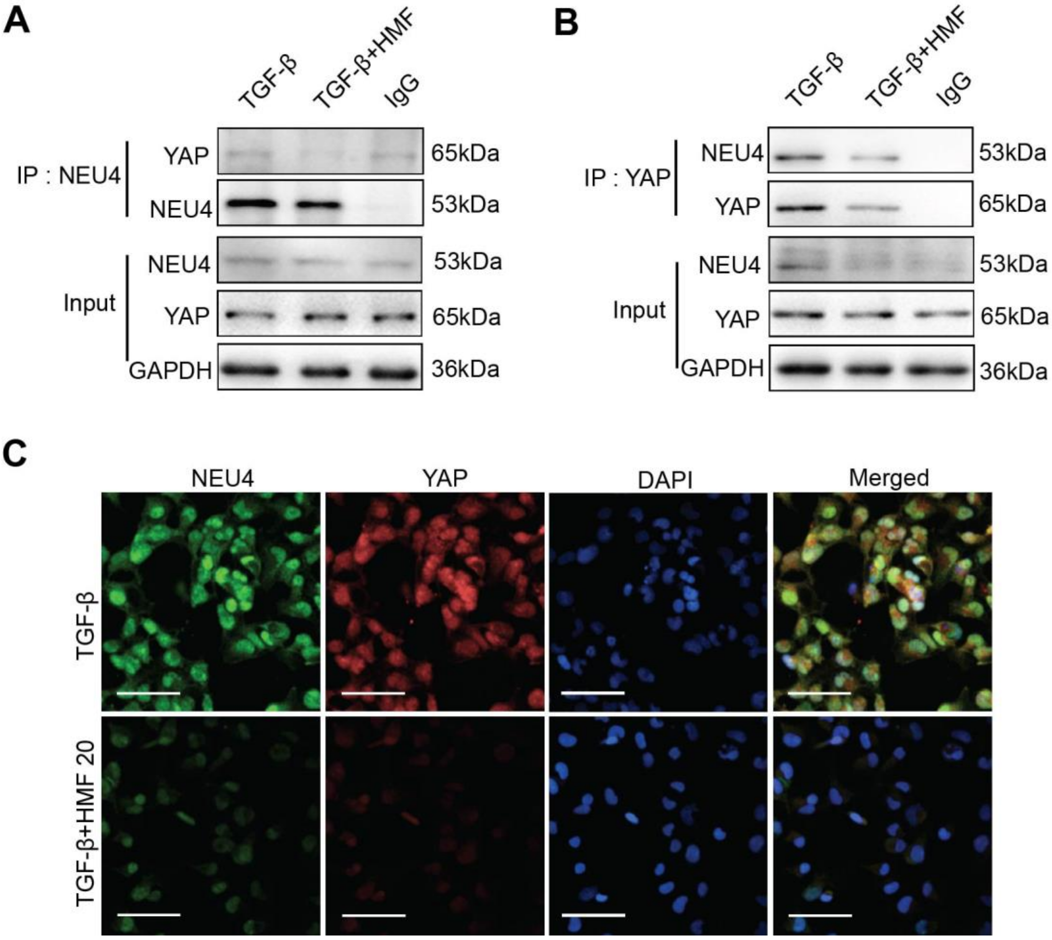

Figure S8. HMF inhibited the interaction between NEU4 and YAP. (**A** and **B**) Western blotting of CoIP of NEU4 and YAP in HK-2 cells treated with TGF-β and in the presence of DMSO or HMF. (**C**) Colocalization of NEU4 and YAP was analyzed by immunofluorescence in HK-2 cells stimulated with TGF-β and in the presence of DMSO or HMF 24 h. Scale bar, 100 μm, $n = 3$ samples.

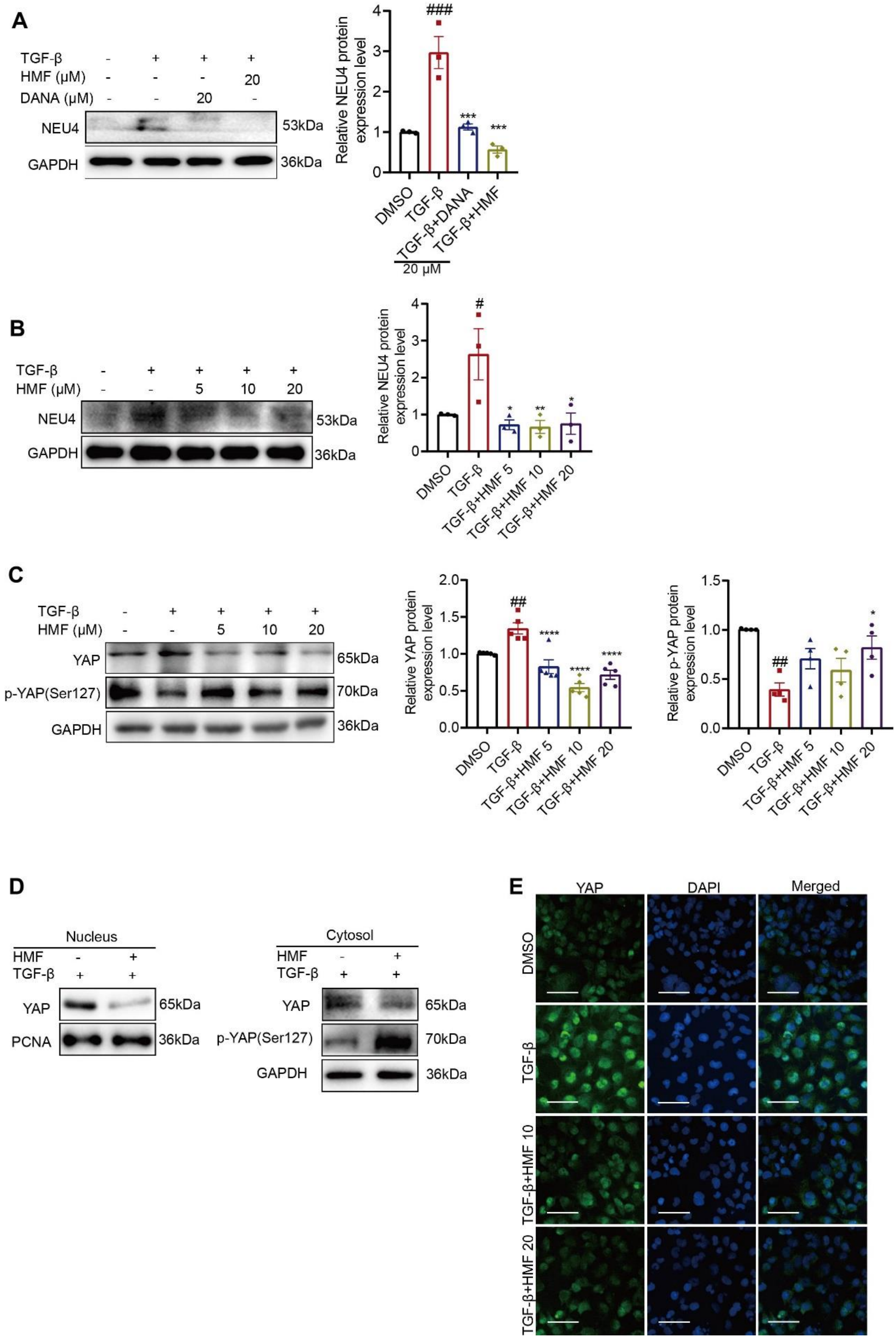

Figure S9. HMF inhibited NEU4 and YAP expression. (**A**) Western blot (left panel) and quantification (right panel) of the protein expression of NEU4 in HK-2 cells 24 h

after treatment with TGF-β and in the presence of DANA or HMF. GAPDH served as loading control, *n* = 3 samples. (**B** and **C**) Western blot (left panel) and quantification (right panel) of the protein expression of NEU4 (**B**), YAP and phosphorylation of YAP (**C**) in HK-2 cells 24 h after treatment with TGF-β and in the presence of DMSO or HMF. GAPDH served as loading control, *n* = 3-5 samples. (**D**) Western blot of the protein expression of YAP in nuclear and cytosol, phosphorylation of YAP in cytosol in HK-2 cells 24 h after treatment with TGF-β and in the presence of DMSO or HMF. (**E**) Immunofluorescent staining represents YAP expressions in HK-2 cells treatment with TGF-β and in the presence of DMSO or HMF 24 h. Scale bar, 100 μm, *n* = 3 samples. Error bars represent mean ± SEM. Comparisons those among three or more groups by using one-way analysis of variance (ANOVA) followed by Dunnett′s post hoc tests. $^{*}p<0.05$, $^{**}p<0.01$, $^{***}p<0.001$, $^{****}p<0.0001$ versus the TGF-β group, $^{\#}p<0.05$, $^{\#\#}p<0.01$, $^{\#\#\#}p<0.001$ versus the DMSO group.

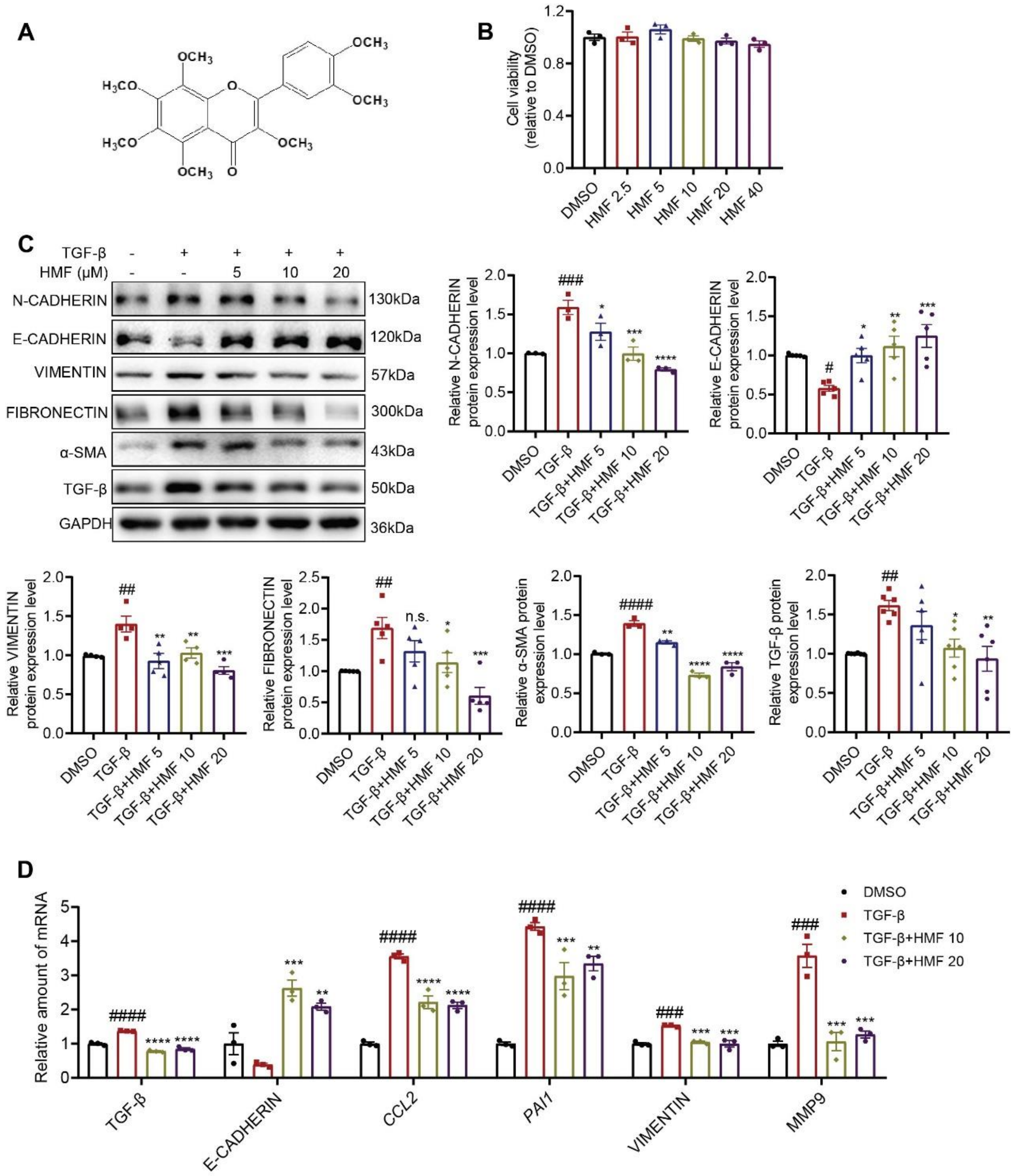

Figure S10. HMF inhibited TGF-β-induced EMT, and the production of proinflammatory cytokines in HK-2. (**A**) The structure of HMF. (**B**) Cell viability after treatment with increasing concentrations of HMF in HK-2 cells for 24 h. n = 3 biologically independent samples. (**C**) Western blot (left panel) and quantification (right panel) of the protein expression of N-CADHERIN, E-CADHERIN, VIMENTIN, FIBRONECTIN, α-SMA and TGF-β in HK-2 cells. GAPDH served as loading control. n = 3-6 biologically independent samples. (**D**) Relative EMT associated gene and extracellular matrix associated gene mRNA level in HK-2 cells. n = 3 biologically

independent samples. (**C** and **D**) HK-2 cells treated with TGF-β and in the presence of DMSO or HMF for 24 h. Error bars represent mean ± SEM. Comparisons those among three or more groups by using one-way analysis of variance (ANOVA) followed by Dunnett′s post hoc tests. $*p<0.05$, $**p<0.01$, $***p<0.001$, $****p<0.0001$ versus the TGF-β group, $^{\#}p<0.05$, $^{\#\#}p<0.001$, $^{\#\#\#}p<0.001$, $^{\#\#\#\#}p<0.0001$ versus the DMSO group.

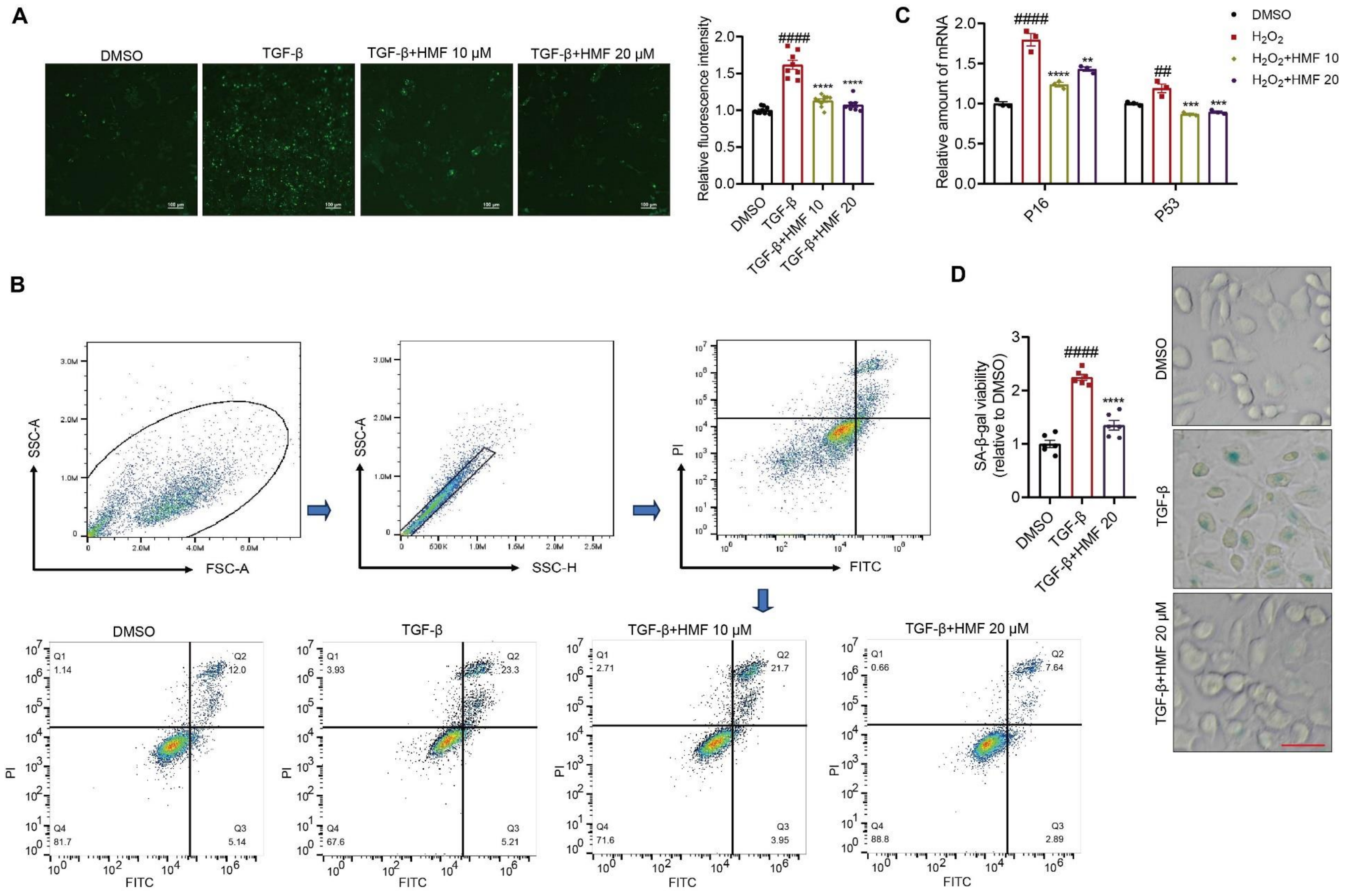
A
DMSO
TGF-β
TGF-β+HMF 10 μM
TGF-β+HMF 20 μM
Relative fluorescence intensity
####
****
****
DMSO
TGF-β
TGF-β+HMF 10
TGF-β+HMF 20
C
Relative amount of mRNA
DMSO
H2O2
H2O2+HMF 10
H2O2+HMF 20
P16
P53
B
SSC-A
FSC-A
SSC-H
PI
FITC
Q1
Q2
Q3
Q4
1.14
12.0
81.7
5.14
3.93
23.3
67.6
5.21
2.71
21.7
71.6
3.95
0.66
7.64
88.8
2.89
D
SA-β-gal viability (relative to DMSO)
TGF-β+HMF 20

Figure S11. HMF ameliorated TGF-β-induced apoptosis and cellular senescence in HK-2. (**A**) Measurement (left panel) and the quantification (right panel) of apoptosis by TUNEL staining in HK-2 cells. Scale bar, 100 μm. $n = 10$ samples. (**B**) Measurement of apoptosis by flow cytometric analysis in HK-2 cells. (**C**) Relative senescence associated gene mRNA level in HK-2 cells after treatment with H_2O_2 in the presence of DMSO or HMF, $n = 3$ biologically independent samples. (**D**) Measurement (right panel) and the quantification (left panel) of SA-β-gal activity by SA-β-gal staining in HK-2 cells. Scale bar, 100 μm. $n = 6$ samples. (A, B and D) HK-2 cells treated with TGF-β and in the presence of DMSO or HMF for 24 h. Error bars represent mean ± SEM. Comparisons those among three or more groups by using one-way analysis of variance (ANOVA) followed by Dunnett′s post hoc tests. $^{**}p<0.01$, $^{***}p<0.001$, $^{****}p<0.0001$ versus the TGF-β or H_2O_2 group, $^{\#\#}p<0.01$, $^{\#\#\#\#}p<0.0001$ versus the DMSO group.

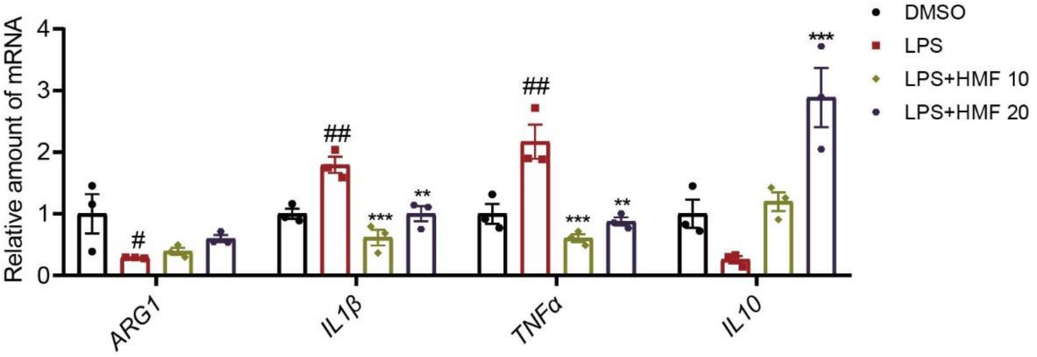

Figure S12. HMF inhibited LPS-induced inflammation in HK-2. Relative inflammation associated gene mRNA level in HK-2 cells 24 h after treatment with LPS in the presence of DMSO or HMF, *n* = 3 biologically independent samples. Error bars represent mean ± SEM. Comparisons those among three or more groups by using one-way analysis of variance (ANOVA) followed by Dunnett′s post hoc tests. $^{**}p<0.01$, $^{***}p<0.001$ versus the LPS group, $^{\#}p<0.05$, $^{\#\#}p<0.01$ versus the DMSO group.

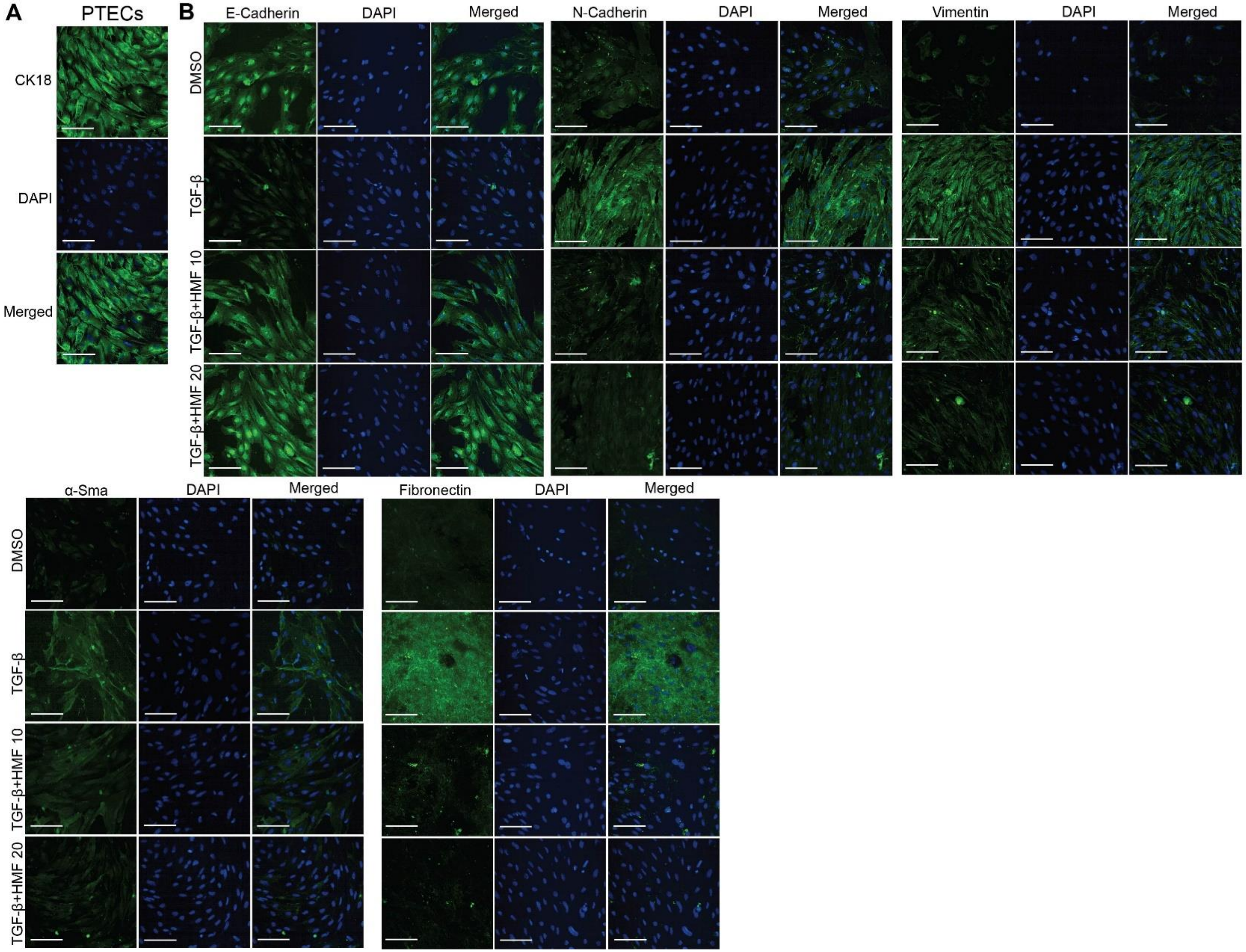
A
PTECs
CK18
DAPI
Merged
B
E-Cadherin
DAPI
Merged
N-Cadherin
DAPI
Merged
Vimentin
DAPI
Merged
DMSO
TGF-β
TGF-β+HMF 10
TGF-β+HMF 20
α-Sma
DAPI
Merged
Fibronectin
DAPI
Merged

Figure S13. HMF inhibited TGF-β-induced EMT in PTECs. (**A**) Immunofluorescent staining represents CK18 expressions in PTECs. Scale bar, 100 μm, n = 3 samples. (**B**) Immunofluorescent staining represents N-Cadherin, E-Cadherin, Vimentin, α-Sma and Fibronectin expressions in PTECs 24 h after treatment with TGF-β and in the presence of DMSO or HMF. Scale bar, 100 μm, n = 3 samples.

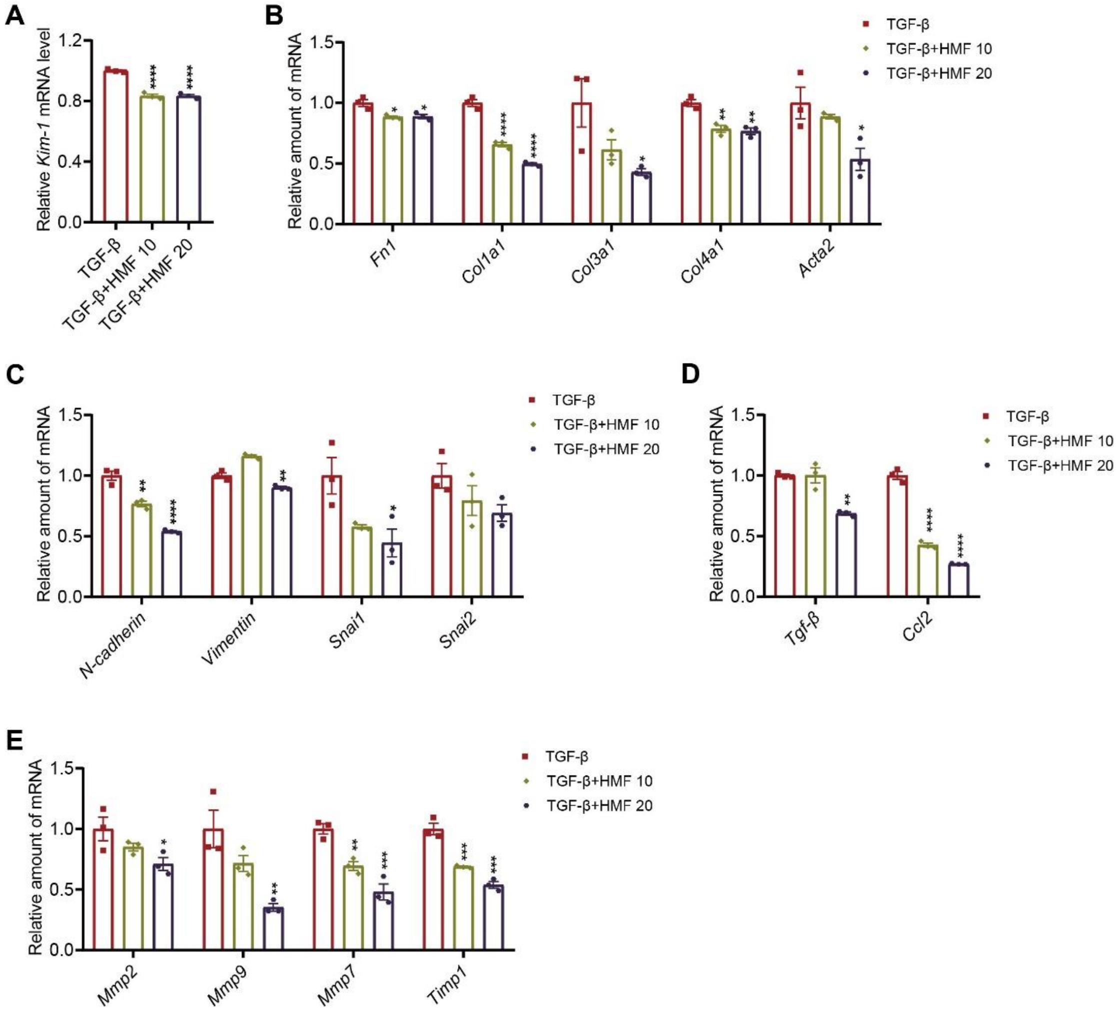

Figure S14. HMF inhibited TGF-β-induced EMT, and the production of proinflammatory cytokines in PTECs. (**A**-**E**) PTECs treated with TGF-β and in the presence of DMSO or HMF for 24 h. (**A**) *Kim-1* mRNA level in PTECs, $n = 3$ biologically independent samples. (**B**-**E**) Relative ECM associated gene (**B**), EMT associated gene (**C**), chemokine associated gene (**D**), and extracellular matrix associated gene (**E**) mRNA level in PTECs, $n = 3$ biologically independent samples. Error bars represent mean ± SEM. Comparisons those among three or more groups by using one-way analysis of variance (ANOVA) followed by Dunnett′s post hoc tests. *$p<0.05$, **$p<0.01$, ***$p<0.001$, ****$p<0.0001$ versus the TGF-β group.

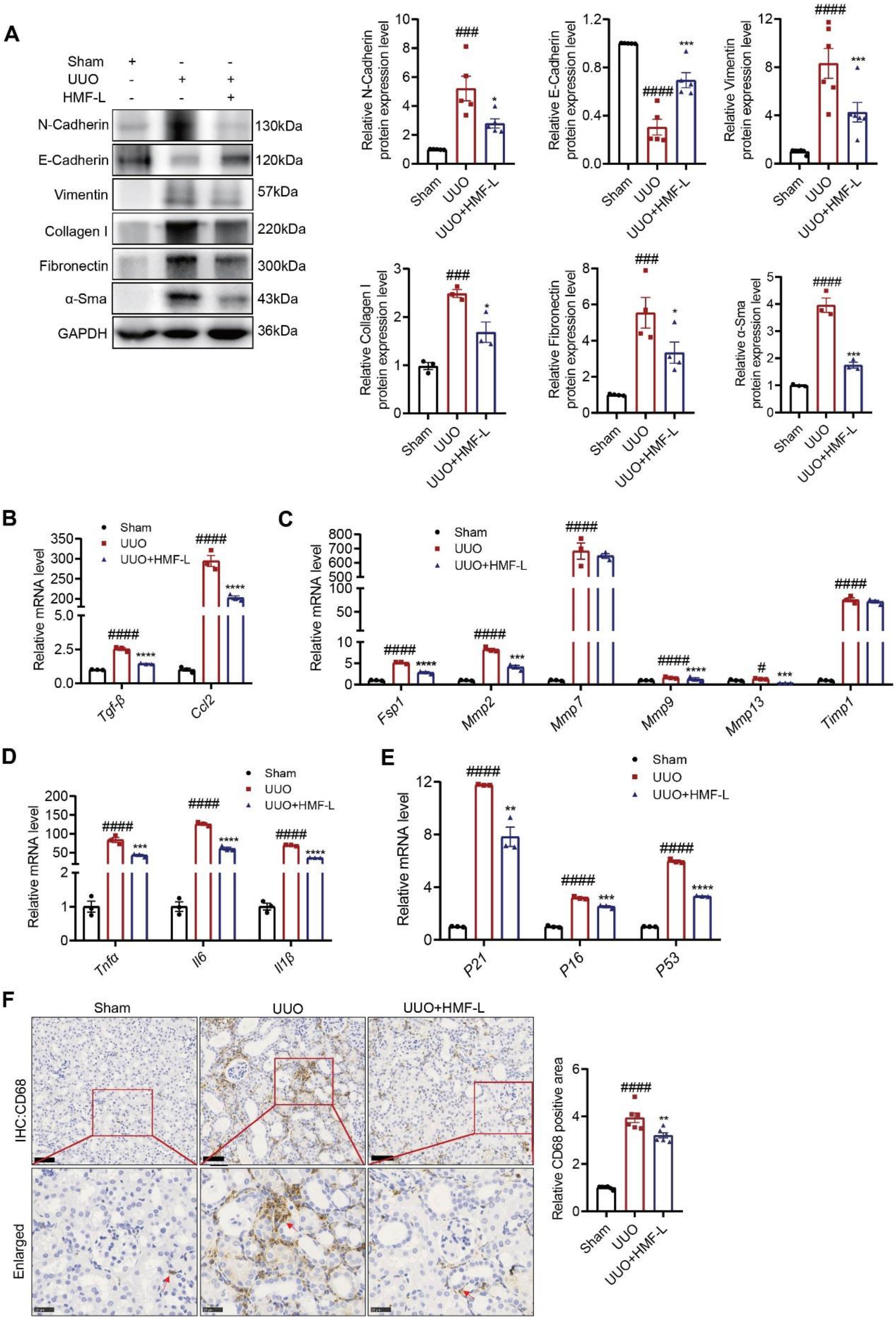

Figure S15. HMF improved UUO-induced renal fibrosis in mice. Vehicle, or HMF (50 or 100 mg/kg/day) was administrated to UUO mice by gastric irrigation once daily

for 10 days. UUO, unilateral ureteral obstruction. (**A**) Western blot (left panel) and quantification (right panel) of the protein expression of N-Cadherin, E-Cadherin, Vimentin, Collagen I, Fibronectin and α-Sma in left kidneys, GAPDH served as loading control, *n* = 3-6 mice. (**B**-**E**) Relative mRNA level of chemokine-associated genes (**B**), extracellular matrix-associated genes (**C**), inflammation associated genes (**D**) and senescence-associated genes (**E**) were determined by RT-qPCR from left kidneys. *n* = 3 biologically independent samples. (**F**) Immunohistochemistry staining analysis (left panel) and quantification (right panel) of CD68 expression in left kidney tissues, *n* = 6 mice. Scale bar, 50 μm. Error bars represent mean ± SEM. Comparisons those among three or more groups by using one-way analysis of variance (ANOVA) followed by Dunnett′s post hoc tests. $^{*}p<0.05$, $^{**}p<0.01$, $^{***}p<0.001$, $^{****}p<0.0001$ versus the UUO group, $^{\#\#\#}p<0.001$, $^{\#\#\#\#}p<0.0001$ versus the Sham group.

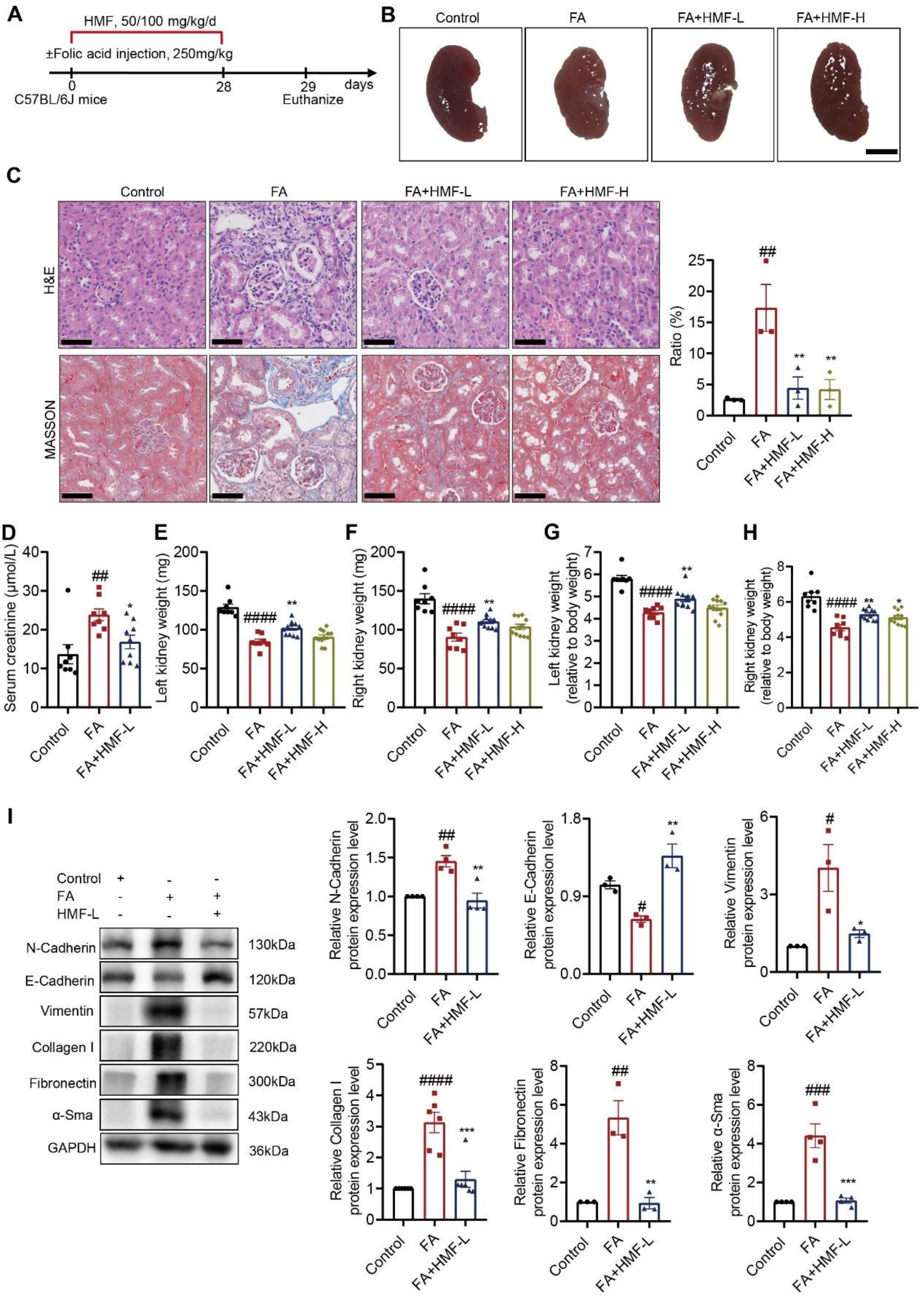

Figure S16. HMF ameliorated FA-induced renal fibrosis in mice. The mice were intraperitoneally injected with folic acid (250 mg/kg). Vehicle, or HMF (50 or 100 mg/kg/day) was administrated to folic acid mice by gastric irrigation once daily for 28 days. (**A**) Scheme of the experimental approach. (**B**) Representative picture of left

kidneys of mice with different treatments. (**C**) Representative photomicrographs of the H&E staining and Masson's trichrome staining from left kidneys, renal interstitial fibrosis scores based on Masson's trichrome staining (right panel), n = 3 mice. Scale bar, 50 μm. (**D**) Creatinine in serum, n = 8-11 mice. (**E** and **F**) The left (**E**) and right (**F**) renal weight. (**G** and **H**) The ratio of left (**G**) and right (**H**) renal weight to body weight (BW), n = 8-11 mice. (**I**) Western blot (left panel) and quantification (right panel) of the protein expression of N-Cadherin, E-Cadherin, Vimentin, Collagen I, Fibronectin and α-Sma in left kidneys, GAPDH served as loading control, n = 3-6 mice. Error bars represent mean ± SEM. Comparisons those among three or more groups by using one-way analysis of variance (ANOVA) followed by Dunnett′s post hoc tests. $*p<0.05$, $**p<0.01$, $***p<0.001$ versus the FA group, $^{\#}p<0.05$, $^{\#\#}p<0.01$, $^{\#\#\#}p<0.001$, $^{\#\#\#\#}p<0.0001$ versus the Control group.

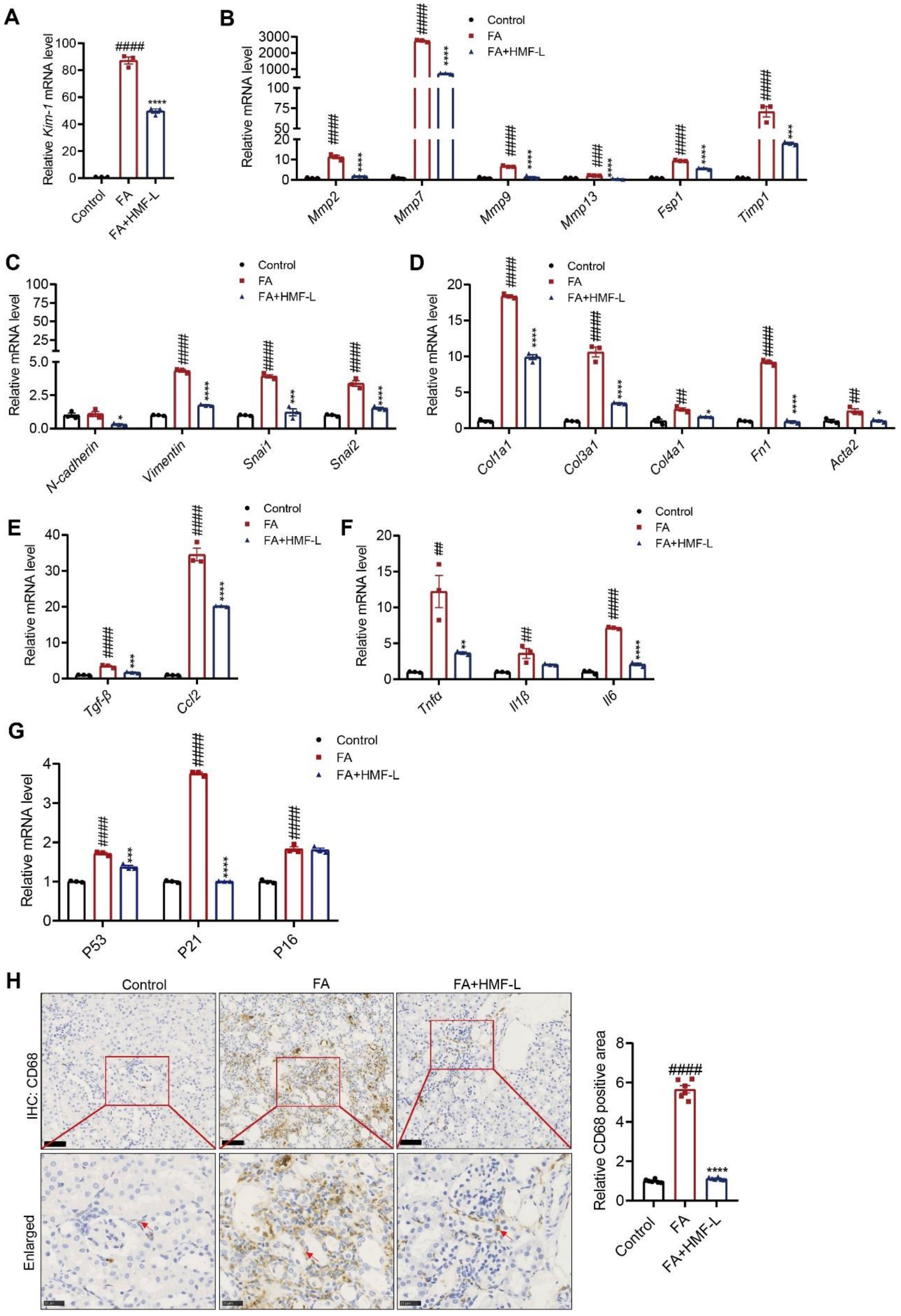

Figure S17. HMF inhibited FA-induced EMT, the production of proinflammatory cytokines, and macrophage infiltration in mice. The mice were intraperitoneally

injected with folic acid (250 mg/kg). Vehicle, or HMF (50 or 100 mg/kg/day) was administrated to folic acid mice by gastric irrigation once daily for 28 days. (**A**) *Kim-1* mRNA level in left kidneys, $n = 3$ mice. (**B**-**G**) Relative extracellular matrix associated gene (**B**), EMT associated gene (**C**), ECM associated gene (**D**), chemokine associated gene (**E**), inflammation associated gene (**F**) and senescence-associated genes (**G**) mRNA level in left kidneys, $n = 3$ mice. (**H**) Immunohistochemistry staining analysis (left panel) and quantification (right panel) of CD68 expression in kidney tissues, $n = 6$ mice. Scale bar, 50 μm. Error bars represent mean ± SEM. Comparisons those among three or more groups by using one-way analysis of variance (ANOVA) followed by Dunnett′s post hoc tests. $^{*}p<0.05$, $^{**}p<0.01$, $^{***}p<0.001$, $^{****}p<0.0001$ versus the FA group, $^{\#\#}p<0.01$, $^{\#\#\#}p<0.001$, $^{\#\#\#\#}p<0.0001$ versus the Control group.

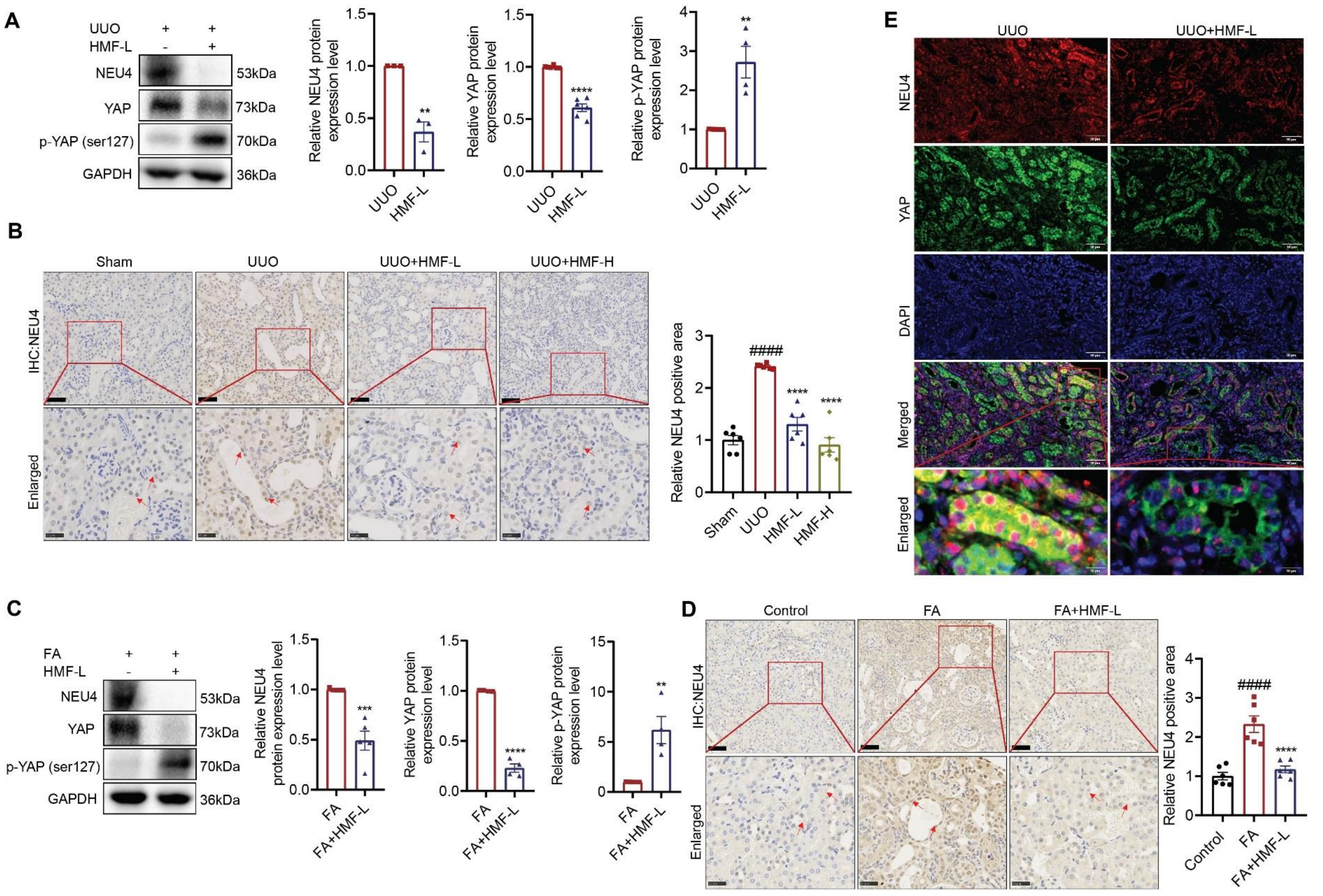
A
UUO + +
HMF-L - +
NEU4
YAP
p-YAP (ser127)
GAPDH
53kDa
73kDa
70kDa
36kDa
Relative NEU4 protein expression level
Relative YAP protein expression level
Relative p-YAP protein expression level
UUO
HMF-L
B
Sham
UUO
UUO+HMF-L
UUO+HMF-H
IHC:NEU4
Enlarged
Relative NEU4 positive area
Sham
UUO
HMF-L
HMF-H
C
FA + +
HMF-L - +
NEU4
YAP
p-YAP (ser127)
GAPDH
53kDa
73kDa
70kDa
36kDa
Relative NEU4 protein expression level
Relative YAP protein expression level
Relative p-YAP protein expression level
FA
FA+HMF-L
D
Control
FA
FA+HMF-L
IHC:NEU4
Enlarged
Relative NEU4 positive area
E
UUO
UUO+HMF-L
NEU4
YAP
DAPI
Merged
Enlarged

Figure S18. HMF inhibited the interaction between NEU4 with YAP, and activation of YAP in UUO or folic acid-induced mice. (**A**) Western blot (left panel) and quantification (right panel) of the protein expression of NEU4, YAP and phosphorylation of YAP in left kidneys. GAPDH served as loading control, n = 3-5 mice. (**B**) Immunohistochemistry staining analysis (left panel) and quantification (right panel) of NEU4 in left kidney tissues. n = 6 mice. Scale bar, 50 μm. (**C**) Western blot (left panel) and quantification (right panel) of the protein expression of NEU4, YAP and phosphorylation of YAP in kidneys. GAPDH served as loading control, n = 3-5 mice. (**D**) Immunohistochemistry staining analysis (left panel) and quantification (right panel) of NEU4 in kidney tissues. n = 6 mice. Scale bar, 50 μm. (**E**) Colocalization of NEU4 and YAP was analyzed by immunofluorescence in left kidneys. Scale bar, 50 μm. n = 6 mice. (**A**, **B** and **E**) Vehicle, or HMF (50 or 100 mg/kg/day) was administrated to UUO mice by gastric irrigation once daily for 10 days. (**C** and **D**) The mice were intraperitoneally injected with folic acid (250 mg/kg). Vehicle, or HMF (50 or 100 mg/kg/day) was administrated to folic acid mice by gastric irrigation once daily for 28 days. Error bars represent mean ± SEM. Comparisons between two groups were analyzed by using a two-tailed Student′s t test. Comparisons those among three or more groups by using one-way analysis of variance (ANOVA) followed by Dunnett′s post hoc tests. $^{**}p<0.01$, $^{***}p<0.001$, $^{****}p<0.0001$ versus the UUO or FA group, $^{\#\#\#\#}p<0.0001$ versus the Sham or Control group.

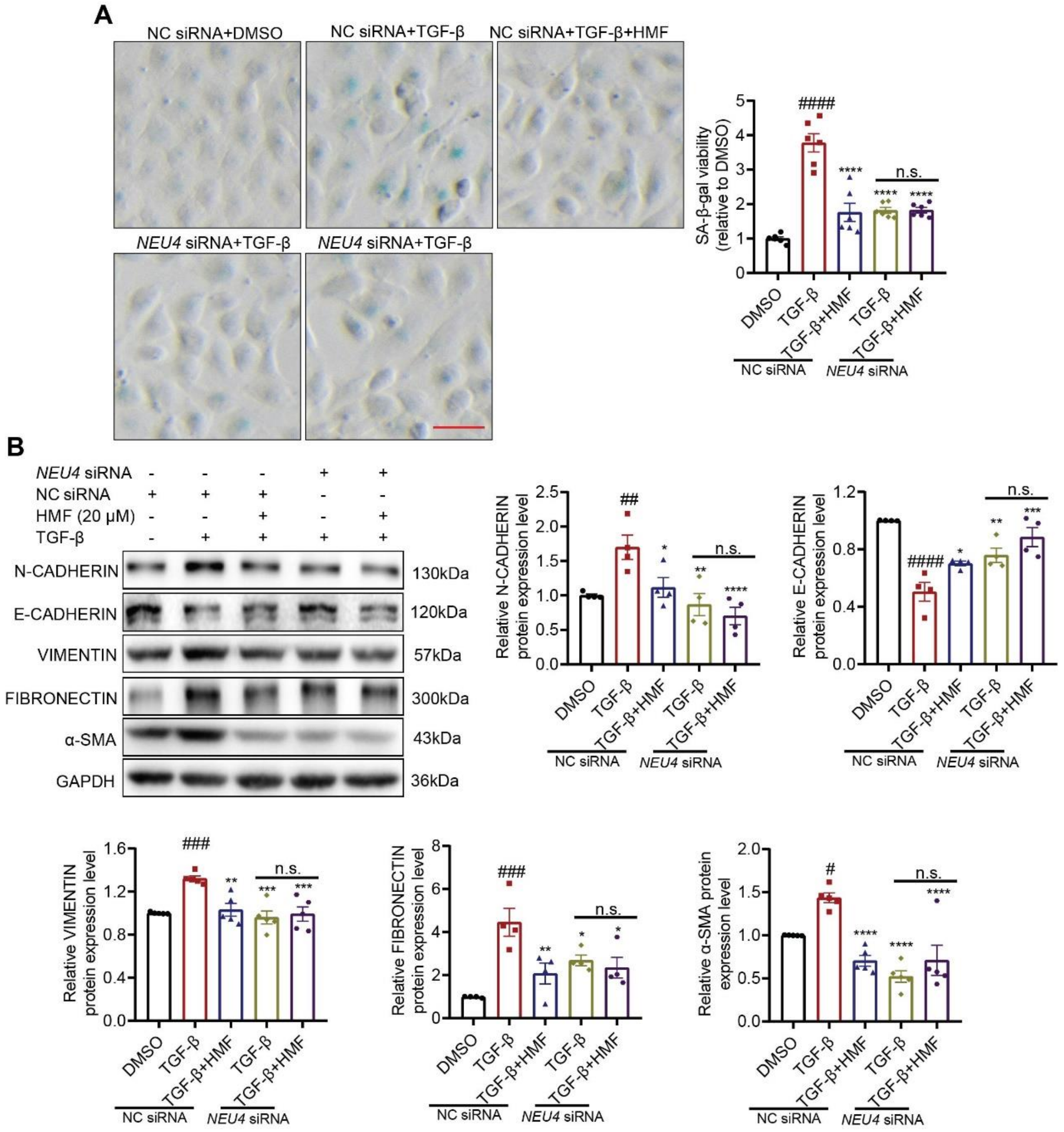

Figure S19. NEU4 knockdown abolished the anti-fibrotic effect of HMF in HK-2. HK-2 cells treatment with TGF-β and in the presence of DMSO or HMF 24 h after transfection with *NEU4* siRNA. (**A**) Measurement (left panel) and quantification (right panel) of SA-β-gal activity by SA-β-gal staining in HK-2 cells, n = 6 biologically independent samples. Scale bar, 100 μm. (**B**) Western blot (left panel) and quantification (right panel and bottom panel) of the protein expression of N-CADHERIN, E-CADHERIN, VIMENTIN, FIBRONECTIN and α-SMA in HK-2 cells. GAPDH served as loading control. n = 4-5 biologically independent samples. Error bars represent mean ± SEM. Comparisons those among three or more groups by using one-way analysis of variance (ANOVA) followed by Dunnett's post hoc tests. *p<0.05, **p<0.01,

$^{***}p<0.001$, $^{****}p<0.0001$ versus the NC siRNA+TGF-β group, $^{\#}p<0.05$, $^{\#\#}p<0.01$, $^{\#\#\#}p<0.001$, $^{\#\#\#\#}p<0.0001$ versus the NC siRNA+DMSO group. n.s.: no significance.

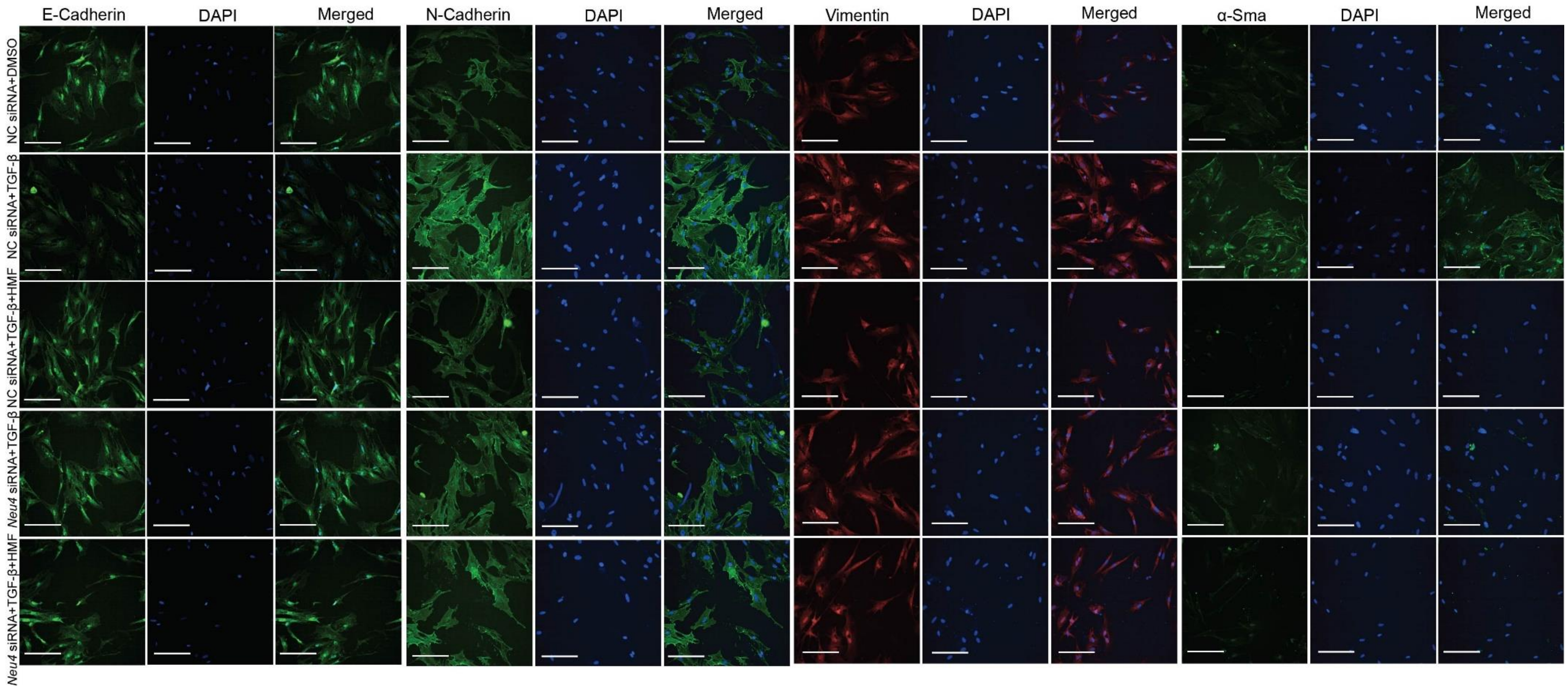

Figure S20. Knockdown of NEU4 abolished the anti-fibrotic effect of HMF in PTECs. Immunofluorescent staining represents N-Cadherin, E-Cadherin, Vimentin and α-Sma expressions in PTECs treatment with TGF-β and in the presence of DMSO or HMF 24 h after transfection with *NEU4* siRNA. $n = 3$ biologically independent samples. Scale bar, 100 μm.

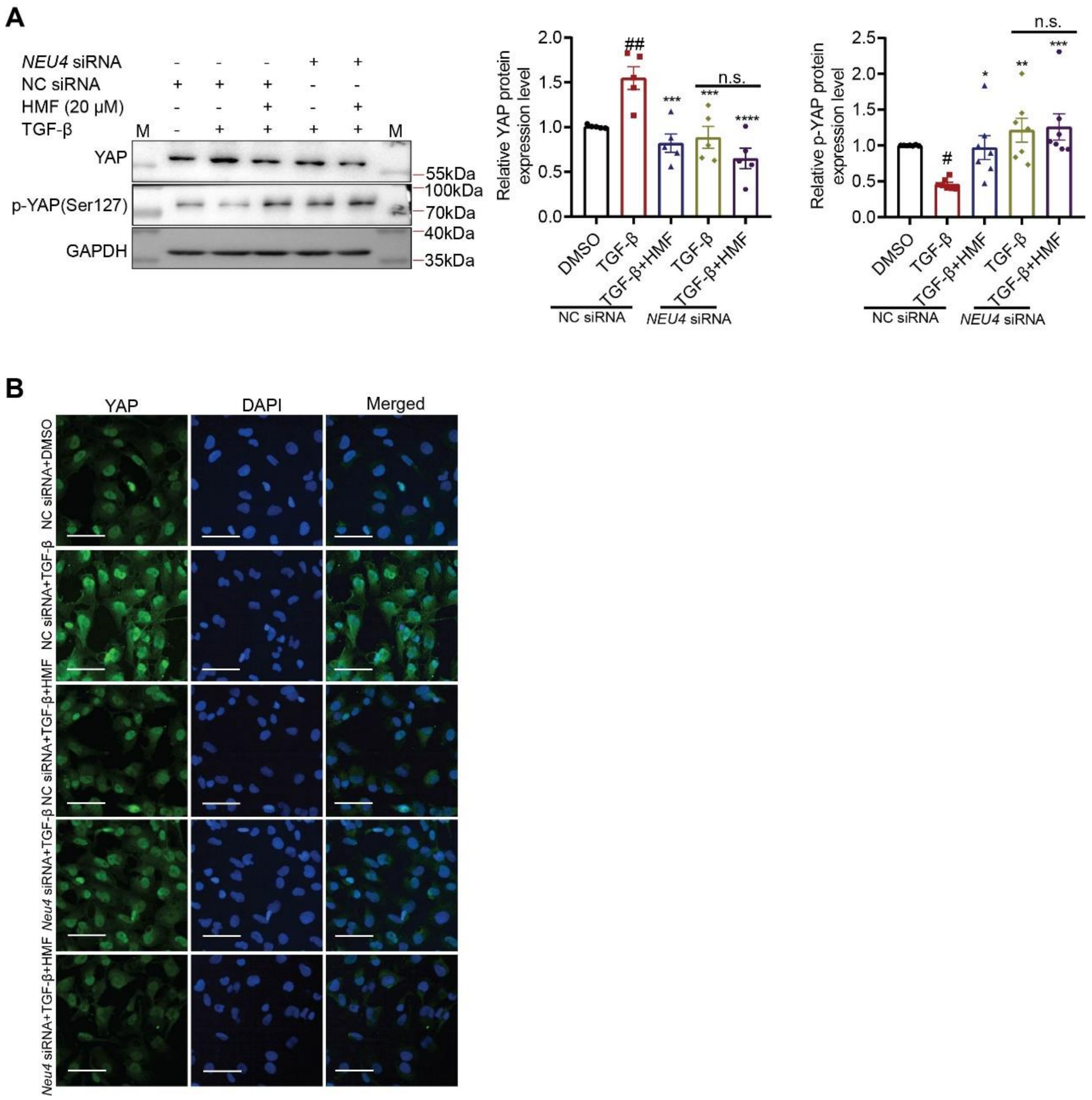

Figure S21. NEU4 knockdown abolished the downregulation of YAP by HMF. HK-2 cells treatment with TGF-β and in the presence of DMSO or HMF 24 h after transfection with *NEU4* siRNA. (**A**) Western blot (left panel) and quantification (right panel) of the protein expression of YAP and phosphorylation of YAP in HK-2 cells. GAPDH served as loading control. n = 5-7 biologically independent samples. M, Marker. (**B**) Immunofluorescent staining represents YAP expressions in HK-2 cells treatment with TGF-β and in the presence of DMSO or HMF 24 h after transfection with *NEU4* siRNA. Scale bar, 100 μm, n = 3 samples. Error bars represent mean ± SEM. Comparisons those among three or more groups by using one-way analysis of variance (ANOVA) followed by Dunnett′s post hoc tests. *p<0.05, **p<0.01, ***p<0.001, ****p<0.0001 versus the NC siRNA+TGF-β group, #p<0.05, ##p<0.001 versus the NC siRNA+DMSO group. n.s.: no significance.

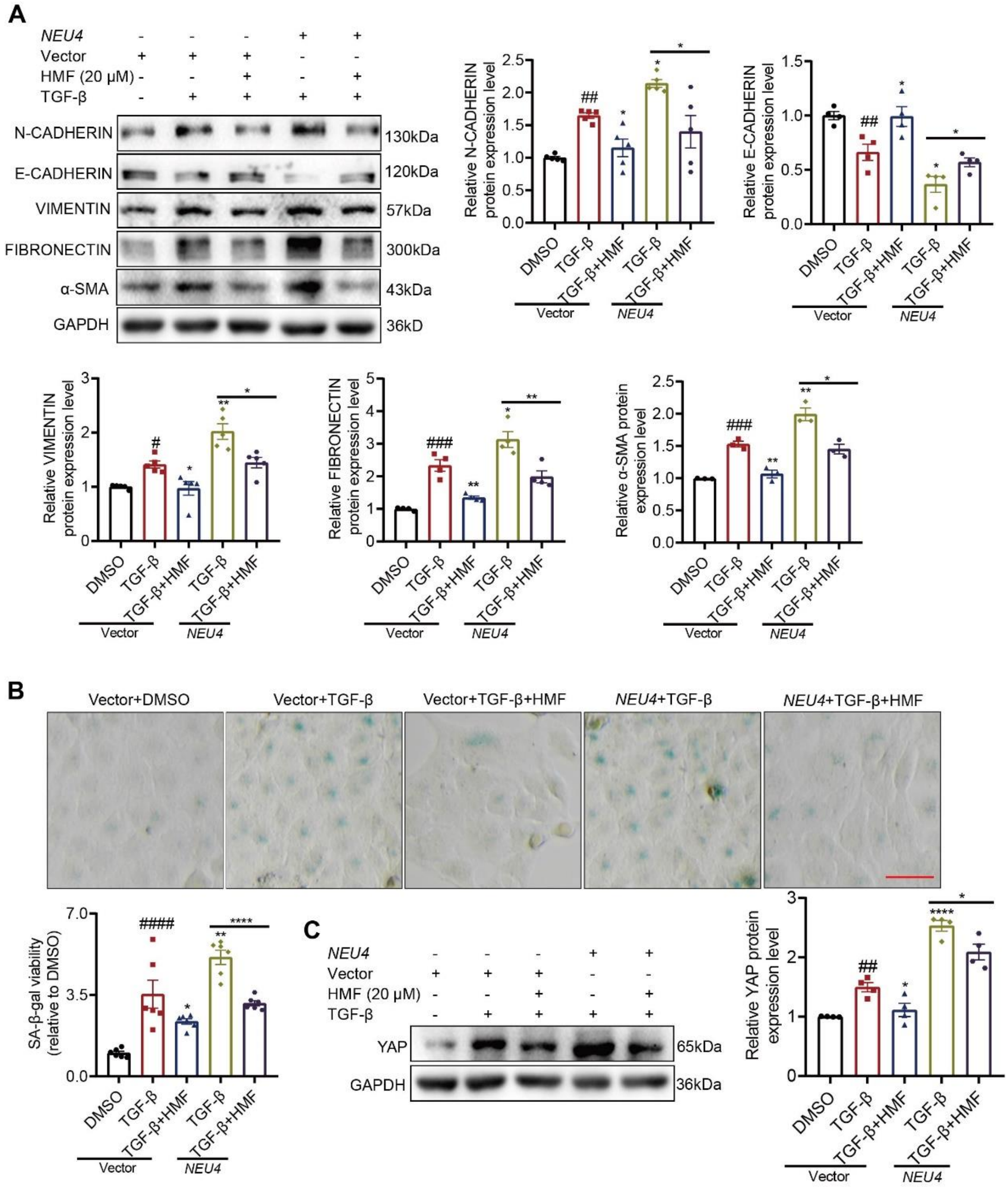

Figure S22. HMF ameliorated TGF-β-induced EMT and cellular senescence is dependent on NEU4 in HK-2. HK-2 cells treated with TGF-β and in the presence of DMSO or HMF 24 h after transfection with *NEU4*-overexpression plasmids. (**A**) Western blot (left panel) and quantification (right panel and bottom panel) of the protein expression of N-CADHERIN, E-CADHERIN, VIMENTIN, FIBRONECTIN and α-SMA in HK-2 cells. GAPDH served as loading control. *n* = 3-6 biologically independent samples. (**B**) Measurement (top panel) and the quantification (bottom panel) of SA-β-gal activity by SA-β-gal staining in HK-2 cells. *n* = 6 biologically

independent samples. Scale bar, 100 μm. (**C**) Western blot (left panel) and quantification (right panel) of the protein expression of YAP in HK-2 cells, GAPDH served as loading control, n = 4 biologically independent samples. Error bars represent mean ± SEM. Comparisons those among three or more groups by using one-way analysis of variance (ANOVA) followed by Dunnett′s post hoc tests. *p<0.05, **p<0.01, ****p<0.0001 versus the Vector+TGF-β or *NEU4*+TGF-β group, $^{\#}p$<0.05, $^{\#\#}p$<0.001, $^{\#\#\#}p$<0.01, $^{\#\#\#\#}p$<0.0001 versus the Vector+DMSO group.

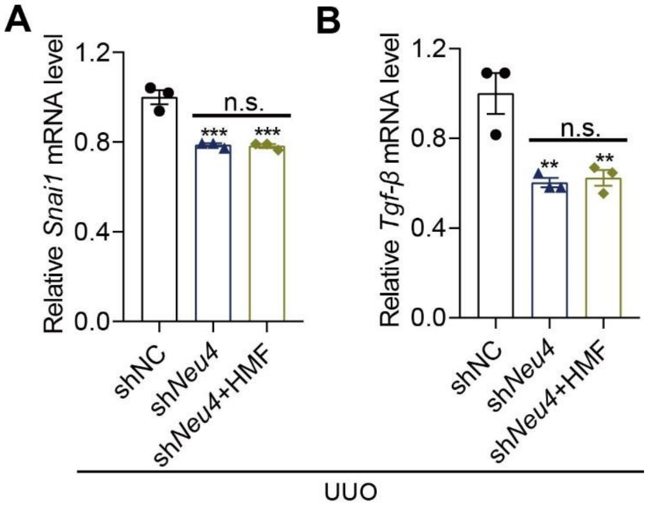

Figure S23. *Neu4* knockdown relieved *Snai1* and *Tgf-β* inhibition effect of HMF in UUO model. Six-week-old male C57BL/6J mice were injected with shNC or sh*Neu4* adenoviruses. Five weeks after injection, the mice were subjected to UUO surgery, then vehicle or HMF (50 mg/kg/day) was administrated to mice by gastric irrigation once daily for 10 days. (**A** and **B**) Relative EMT associated gene mRNA level in left kidneys, $n = 3$ mice. Error bars represent mean ± SEM. Comparisons those among three or more groups by using one-way analysis of variance (ANOVA) followed by Dunnett′s post hoc tests. **p<0.01, ***p<0.001 versus the shNC group. n.s.: no significance.

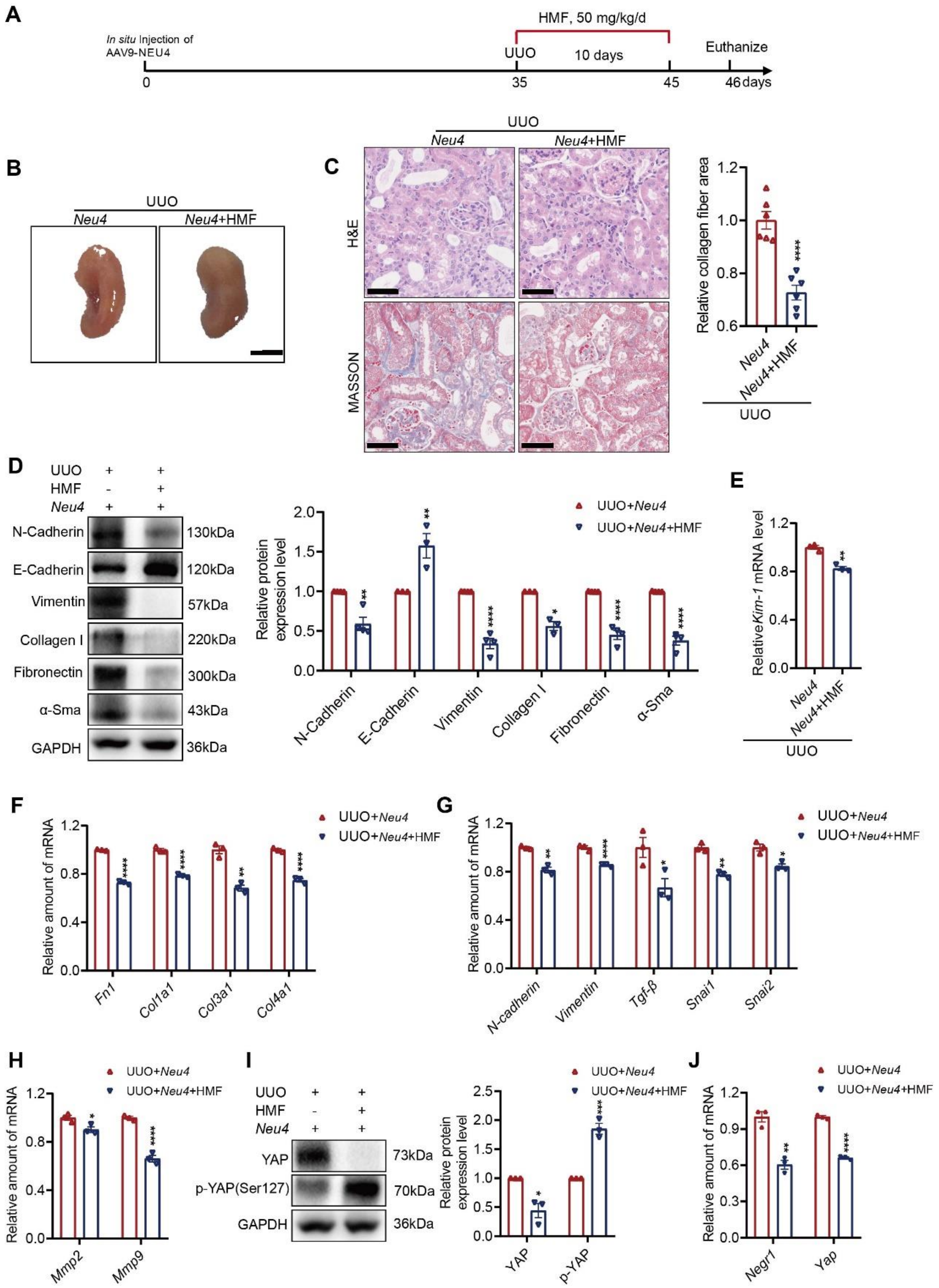

Figure S24. Relief of renal fibrosis by HMF was dependent on NEU4. Mice was *in situ* injected with AAV9 encoding GFP-*Neu4*. Five weeks after injection, the mice were subjected to UUO surgery, then vehicle or HMF (50 mg/kg/day) was administrated to mice by gastric irrigation once daily for 10 days. (**A**) The schematic of experimental

design. (**B**) Representative picture of left kidneys of UUO mice. Scale bar, 100 μm, *n* = 3 mice. (**C**) Representative photomicrographs of the H&E staining and Masson's trichrome staining from left kidneys of UUO mice (left panel), and renal interstitial fibrosis scores based on Masson's trichrome staining (right panel). *n* = 6 mice. H&E staining, scale bar, 50 μm. Masson's trichrome staining, scale bar, 100 μm. (**D**) Western blot (left panel) and quantification (right panel) of the protein expression of N-Cadherin, E-Cadherin, Vimentin, Collagen I, Fibronectin and α-Sma in kidneys, GAPDH served as loading control. *n* = 3-4 mice. (**E**) *Kim-1* gene mRNA level in left kidneys. *n* = 3 mice. (**F**-**H**) Relative ECM associated gene (**F**), EMT associated gene (**G**), extracellular matrix associated gene (**H**) mRNA level in left kidneys. *n* = 3 mice. (**I**) Western blot (left panel) and quantification (right panel) of the protein expression of YAP and phosphorylation of YAP in kidneys. GAPDH served as loading control, *n* = 3 mice. (**J**) Relative mRNA abundance of *Yap* target genes in left kidney tissue, *n* = 3 mice. Error bars represent mean ± SEM. Comparisons between two groups were analyzed by using a two-tailed Student's *t* test. *$p<0.05$, **$p<0.01$, ***$p<0.001$, ****$p<0.0001$ versus the *Neu4* group.
